# Heitt Mjölnir: a heated miniature triaxial apparatus for 4D synchrotron microtomography

**DOI:** 10.1107/S1600577523009876

**Published:** 2024-01-01

**Authors:** Damien Freitas, Ian B. Butler, Stephen C. Elphick, James Gilgannon, Roberto E. Rizzo, Oliver Plümper, John Wheeler, Christian M. Schlepütz, Federica Marone, Florian Fusseis

**Affiliations:** a School of Geosciences, University of Edinburgh, James Hutton Road, The King’s Buildings, Edinburgh EH9 3FE, United Kingdom; b University of Manchester, Diamond Light Source, Harwell Campus, Didcot OX11 0DE, United Kingdom; c Department of Earth Sciences, University of Florence, Via La Pira 4, 50121 Florence, Italy; d Department of Earth Sciences, Utrecht University, Budapestlaan 4, 3584 CD Utrecht, The Netherlands; e Department of Earth, Ocean and Ecological Sciences, University of Liverpool, 4 Brownlow Street, Liverpool L69 3GP, United Kingdom; f Swiss Light Source, Paul Scherrer Institute, Forschungsstrasse 111, 5232 Villigen PSI, Switzerland; University of Malaga, Spain

**Keywords:** synchrotron X-ray microtomography, experimental geosciences, rock deformation, fluid–rock interactions, *in situ* experiments

## Abstract

Third- and fourth-generation synchrotron light sources with high fluxes and beam energies enable the use of innovative X-ray translucent experimental apparatus. These experimental devices access geologically relevant conditions whilst enabling *in situ* characterization using the spatial and temporal resolutions accessible at imaging beamlines. Here, Heitt Mjölnir is introduced, a heated miniature triaxial rig based on the design of Mjölnir, but covering a wider temperature range and larger sample volume at similar pressure capacities. This device is designed to investigate coupled thermal, chemical, hydraulic and mechanical processes from grain to centimetre scales using cylindrical samples of 10 mm × 20 mm (diameter × length). Heitt Mjölnir can simultaneously reach confining (hydraulic) pressures of 30 MPa and 500 MPa of axial stress with independently controlled sample pore fluid pressure < 30 MPa. This internally heated apparatus operates to temperatures up to 573 K with a minimal vertical thermal gradient in the sample of <0.3 K mm^−1^. This new apparatus has been deployed in *operando* studies at the TOMCAT (Swiss Light Source), I12 JEEP (Diamond Light Source) and PSICHÉ (Synchrotron SOLEIL) beamlines for 4D X-ray microtomography with scan intervals of a few minutes. Heitt Mjölnir is portable and modular, allowing a wide range of 4D characterizations of low-grade metamorphism and deformational processes. It enables spatially and temporally resolved fluid–rock interaction studies at conditions of crustal reservoirs and is suitable for characterization of material properties in geothermal, carbonation or subsurface gas storage applications. Technical drawings and an operation guide are included in this publication.

## Abstract 2

Heitt Mjolnir, a heated miniature triaxial apparatus for 4D synchrotron microtomography, is described. This new device combines the capacities of a deformation rig and a thermal reactor with large sample volumes and is suitable for a large range of geo-energy applications.

## 1. Introduction

X-ray micro-computed tomography (µCT) is a powerful imaging technique for the three-dimensional (3D) characterization of samples using laboratory X-ray sources or synchrotron radiation facilities. It allows the direct, non-invasive observation and non-destructive analysis of a wide range of samples, yielding direct insights into their microstructure. The technique also allows investigation of processes through *in situ/operando* testing, where X-ray translucent experimental sample environments benefit from the high photon flux and energies available at X-ray sources to enable time-resolved tomography (4DµCT).

4DµCT studies enable a full visualization of transient processes at the grain scale in a controlled environment, complementing *ex situ* experiments and significantly advancing the capabilities of experimental geosciences. Due to their complexity, 4DµCT experiments are often significantly more challenging than conventional laboratory-based experiments, and the resulting 4D image data more complex to process. They are also more likely to be limited by time duration constraints (for example, a limited number of synchrotron shifts).

Confining the sample in a pressure vessel allows for the characterization and testing of material properties at conditions similar to those in the Earth’s crust. Applications in the geosciences are numerous, where elevated conditions of pressure and temperature are reached in the uppermost kilometres of the Earth and interact with a wide range of anthropogenic interfaces with the geosphere. ‘Traditional’ experimental devices are made to be stiff, and temperature- and pressure-resistant. They use high-strength materials, such as hardened steels and heavy metal carbides that render them opaque to the X-ray beams from modern sources, thus precluding direct imaging observations, even with advanced techniques such as synchrotron X-ray or neutron µCT. Grain-scale developments that influence the bulk behaviour of a sample can therefore only be assessed in *post mortem* studies. X-ray translucent *operando* environments, such as the one presented here, overcome these limitations. The recent upgrades toward the third and fourth generation of synchrotron light sources, as well as improvements in detector capacities (Mokso *et al.*, 2017 ▸), have significantly enhanced the quality and spatial–temporal resolution of X-ray images (Buurlage *et al.*, 2019 ▸; Guignot *et al.*, 2020 ▸; Yu *et al.*, 2016 ▸). Synchrotron sources are particularly suited for *in situ/operando* experiments because their (nearly) parallel beam geometry allows greater freedom in experiment design, with large source–sample–detector distances (cone beam laboratory-based CT scanners often require small source–sample distances). The partially coherent beam can also provide propagation-based phase contrast as a mechanism to enhance contrast sensitivity (edge enhancement) and yield higher imaging quality in given circumstances. A sufficiently high X-ray flux at higher energies has opened up the possibility of using thicker-walled pressure vessels and/or higher-atomic-number materials while achieving spatial resolutions down to a few micrometres voxel edge length (Gualda *et al.*, 2010 ▸; Cnudde & Boone, 2013 ▸). Moreover, these improvements have also introduced the ability to explore the temporal dimension because the resolution of 4DµCT now available at synchrotron light sources (seconds to minutes) is compatible with the duration of many grain-scale transformations of geological materials (Marone *et al.*, 2020 ▸).

These recent advances in imaging capabilities have led to the development of new instrumentation for the direct and time-resolved observations of dynamic geological processes (*e.g.* Noiriel & Renard, 2022 ▸). The primary challenge for characterizing such processes in 4D lies in the capacity limitations of bespoke testing devices in relation to their operating pressure and temperature ranges as well as their size. The design of these tools usually represents a compromise between X-ray transparency (translating into spatial and temporal resolution) and the properties of the materials used to make the pressure vessel (which determines the maximum operating pressures and temperatures). The need for X-ray transparency at high mechanical loads has been achieved through the development of miniature rock deformation apparatus which work on smaller samples than conventional laboratory apparatus. These smaller samples allow the application of reduced forces to reach high pressures, which can be accommodated by the X-ray translucent materials of the cell. Recent contributions have provided a large set of triaxial testing devices for operando laboratory or synchrotron X-ray µCT experiments (Viggiani *et al.*, 2004 ▸; Lenoir *et al.*, 2007 ▸; Tisato *et al.*, 2014 ▸; Renard *et al.*, 2016 ▸; Glatz *et al.*, 2018 ▸; Voltolini *et al.*, 2019 ▸; Butler *et al.*, 2020 ▸; Cartwright-Taylor *et al.*, 2022 ▸; Noiriel & Renard, 2022 ▸).

While the development of X-ray translucent rock physics apparatus is still ongoing, the current capabilities of *operando* apparatus remain largely below their off-line, laboratory-based equivalents, especially in terms of pressures and temperature. Offline devices have gradually incorporated indirect *in situ* testing modules to examine specific variables such as acoustic emissions, electrical conductivities (*e.g.* Lockner *et al.*, 1991 ▸; Brantut *et al.*, 2011 ▸) and fluid permeability (*e.g.* Leclère *et al.*, 2016 ▸), alongside advanced mechanical tests (*e.g.* Rutter *et al.*, 2009 ▸; Llana-Fúnez *et al.*, 2012 ▸). Although these developments can be integrated in novel X-ray translucent *in situ* imaging apparatus (Cartwright-Taylor *et al.*, 2022 ▸), their lighter designs and generally smaller dimensions often limit their capabilities in multimodal experiments. Efforts have been made towards more versatile designs (*e.g.* Fusseis *et al*, 2014 ▸; Butler *et al.*, 2020 ▸), but most apparatus remain specific to particular research questions. For instance, many X-ray translucent mechanical testing devices lack temperature control (Viggiani *et al.*, 2004 ▸; Lenoir *et al.*, 2007 ▸; Tisato *et al.*, 2014 ▸; Cartwright-Taylor *et al.*, 2022 ▸) or offer only a limited temperature range (Butler *et al.*, 2020 ▸; Renard *et al.*, 2016 ▸). Moreover, many newly developed rigs lack comprehensive documentation such as blueprints, calibrations (pressure/load, machine stiffness, thermal gradients) and modification guidelines, hindering widespread adoption in the user community. Consequently, 4DµCT studies on crustal processes remain scarce and limited to specialist research groups.

This paper details the design, construction and operation of the Heitt Mjölnir rig. The device is a versatile apparatus that combines the capacities of a triaxial rig and a thermal reactor that enables *operando* 4DµCT characterization.

Heitt Mjölnir is a device named after the Norse god Thor’s hammer, and ‘Heitt’, the Icelandic word for ‘hot’. It draws inspiration from the design principles of the Mjölnir rig (Butler *et al.*, 2020 ▸) while extending its capabilities and upholding the core ideas of modularity, portability and replicability for the imaging beamline user community. Migration of the heating system from an external to an internal location is the core of Heitt Mjölnir’s development. The geometry of the surrounding parts optimizes heating efficiency and ensures uniform sample heat distribution. This rig represents a significant advancement by combining the best temperature performance of the existing heated X-ray translucent triaxial rigs (Renard *et al.*, 2016 ▸; Voltolini *et al.*, 2019 ▸; Glatz *et al.*, 2018 ▸) with the high-pressure capacities of the non-heated versions. Heitt Mjölnir was designed to use large cylindrical samples (10 mm diameter and 20 mm length). It operates at temperatures of up to 573 K, while maintaining maximum pressure capacities of 30 MPa confining and pore fluid pressures and a differential stress of 500 MPa. This device has been designed to be X-ray translucent to synchrotron beams at intermediate energies (filtered white/monochromatic beams with peak energy above 50 keV) available at current third-generation synchrotron radiation facilities. With the capability to obtain high-quality tomography volumes in just a few minutes, Heitt Mjölnir operates at an imaging frequency compatible with many grain-scale processes in geomaterials.

This paper aims to provide readers with all the elements to understand, replicate and use Heitt Mjölnir. It offers a comprehensive step-by-step guide, from a list of readily available engineering materials to detailed blueprints and machining steps. Extensive calibration and testing protocols, provided in the supporting information, are included along with guidelines for operating the rig at synchrotron radiation facility beamlines. Heitt Mjölnir does not require any dedicated software or specific infrastructure. It uses a non-toxic liquid/hydraulic pressure medium, ensuring compliance with safety requirements for most imaging beamlines. We stress that anyone aiming to replicate Heitt Mjölnir must ensure that they follow the statutory safety requirements of the country and institution where they are based and where the replicate cell will be used to ensure that the cell is appropriately assessed, tested and approved for the planned experimental applications.

Heitt Mjölnir constitutes a design that overcomes major technical challenges to enable high pressure and temperature testing while acquiring synchrotron 4DµCT data. It is particularly adapted to geosciences applications that explore thermal, mechanical, hydraulic and chemical processes, bridging the gap between grain-scale developments and the resulting macroscale responses. Combined, Mjolnir (Butler *et al.*, 2020 ▸), Heitt Mjölnir and their future derivatives form an experimental platform to study crustal rock deformation, metamorphic processes and fluid–rock interactions in subsurface geoenergy application storage and exploitation.

## 2. Design and construction

Heitt Mjölnir’s design follows the Hoek cell geometry, widely used in rock triaxial deformation experiments (Hoek & Franklin, 1968 ▸). Radial confining pressure (σ_2_ = σ_3_ = *P*_c_) is controlled by a fluid in a sealed metal pressure vessel. Differential stress is generated via axial solid compression applied to the sample (σ_1_). The differential stress (Δσ) on the sample is defined as a function of the axial stress and confining pressure (Δσ = σ_1_ − *P*_c_). The effective differential stress on the sample is defined as: Δσ_eff_ = σ_1_ − *P*_c_ − *P*_f_, with *P*_f_ being the pore fluid pressure in the sample.

**Figure 1:**
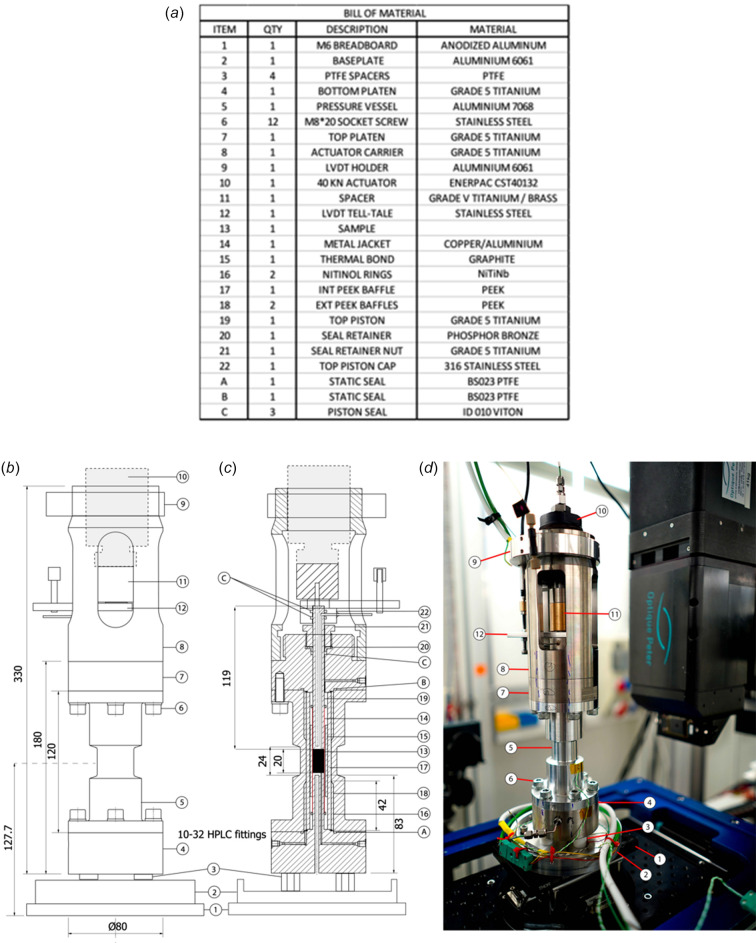
Bill of materials (*a*), outer and inner dimensions (*b*, *c*) and sectional drawing (*c*) of Heitt Mjölnir, and a photograph of the apparatus deployed at the TOMCAT beamline of the Swiss Light Source synchrotron at PSI, Switzerland (*d*). Numbers and letters labelled on parts (*b*), (*c*) and (*d*) refer to items on the bill of material (*a*). CAD drawings for each component are provided in the supporting information. All dimensions are in mm.

The rig, fully assembled, weighs about 6 kg, and stands 330 mm tall (Figs. 1 ▸ and 2 ▸) with a distance between the bottom of the base plate to the sample centre of 127.7 mm. The parts outside the X-ray beam path are made of grade 5 titanium. Components within the beam path are made from materials with greater X-ray transparency including 7068 aluminium alloy, graphite and polyether ether ketone (PEEK), except for the thin (<200 µm wall) copper jacket surrounding the sample (see below and Section S2 of the supporting information). O-ring seals are Viton or polytetra­fluoro­ethyl­ene (PTFE). All fluid connections to the cell use 10/32 high-performance liquid chromatography (HPLC) connectors. A bill of materials and a cross-section drawing of the apparatus are shown in Fig. 1 ▸ and in the supporting information (Section S1, Tables S1 and S2). Technical drawings of all components are listed in Section S1 of the supporting information and provided in separate files. Notes on construction detailing the design choices and operation mode of the rig are given in Section S2 of the supporting information. In the following section, the different parts of the device are described from the core (sample) outwards to the external parts.

In the centre of the rig, the cylindrical sample is held between the two vertical pistons and is sleeved in a thermally annealed copper jacket (see Section S2 of the supporting information, and Fig. 3 ▸) of 200 µm wall thickness. This jacket is sealed on each end against the pistons via a set of UniLok NiTiNb heat-shrinkable metal rings (Intrinsic Devices). A high thermal conductivity graphite sleeve (grade 6507) is slid over the jacket. Both the jacket and the graphite sleeve thermally bond the pistons and sample to provide a homogeneous heat distribution in the sample zone (see Section S3 and Fig. S2 of the supporting information). We chose copper as the jacket material because of the high temperatures reached in the sample zone, the need for heat conduction performance and pressure transmission on the sample, while making a reliable seal against the confining fluid (ductility). A detailed discussion on the constraints and alternative materials can be found in Section S2.1 of the supporting information.

The pistons are made of grade 5 titanium, selected for its high tensile strength and temperature resistance (yield strength at 0.2% elongation of ∼850 MPa at 298 K and ∼730 MPa at 623 K). The top piston is made from a 10 mm centreless ground bar of titanium. The bottom piston is part of the bottom platen of the cell. This bottom platen is connected to the pressure vessel via six tensile grade 12.9 M8 socket screws. It is sealed using a BS023 PTFE O-ring. Both bottom and top pistons were drilled with a 3.2 mm-diameter hole to pass the 1/8" cartridge heating element and a 1 mm-diameter hole for pore fluid access (see the CAD-drawing in Section S1 and details in Section S2 of the supporting information).

The pressure vessel is a critical element of this apparatus. It must balance strength and stiffness against a minimal X-ray attenuation. In a heated device, the pressure vessel must retain its physical properties over the largest temperature range possible and remain unreactive to the confining fluids. To achieve this, we selected aluminium 7068 alloy, which is lightweight (moderate X-ray attenuation) yet has high tensile strength. This alloy offers a good yield strength (700 MPa at 0.2% elongation and 297 K) and retains most of its mechanical properties up to 473 K. Above that, the alloy strength decreases between 473 and 523 K, requiring careful thermal management of the cell temperature (see below). Alloy 7068 also offers good resistance to corrosion if used with silicone oil. Estimates of the radial, axial and hoop stress on the inner surface of the vessel (where hoop stress is maximal) indicate a total (axial + radial + hoop) stress of 258 MPa, at a confining pressure of 30 MPa and axial load of 40 kN (510 MPa on a 10 mm-diameter sample). This is significantly less than the alloy yield strength at temperatures above 373 K (about 590 MPa). The present configuration offers a safety factor of at least 2.3 (ratio of maximum stress over strength of the pressure vessel). In practice, the pressure vessel was designed for repeated and safe use at confining below pressures of 30 MPa and axial load of 40 kN, at maximum sample temperatures of 573 K (corresponding to a pressure vessel temperature of <373 K). Heitt Mjölnir has been designed for synchrotron beam times and we consider prolonged runs to last a maximum of one week. Before all experimental run series, the pressure vessel should be hydraulically tested beyond its operating loads in conformity with local pressure safety requirements. Given that the strength of the pressure vessel will degrade over time due to temperature and pressure cycling, this step is an obligatory safety requirement (see guidelines in Section 2.10 of the supporting information).

As noted previously, the pressure vessel must be protected from the very high temperatures reached in the sample area. Therefore, a set of PEEK baffles limit the volume of confining fluid, its advective motion and minimize radial conductive heat transfer thanks to the low thermal conductivity of PEEK (0.25 W m^−1^ K^−1^) and its high melting point (616 K) (Fig. 1 ▸, and Figs. S1 and S2 of the supporting information). This baffling combined with an external air-cooling stream onto the pressure vessel proved sufficient to keep its outside surface temperature below 373 K (see supporting Section S2 for details). As a safety indicator, we recommend always using the cell with a thermocouple fixed on the outer surface of the pressure vessel to monitor its efficient cooling below 373 K (see supporting Section S4).

The uppermost section of the rig generates and delivers the axial load to the sample and includes access for heating elements and pore fluid. The top end of the pressure vessel is bolted to the top platen and sealed in the same way as the bottom platen. The platen has a central hole to fit the top piston and a groove to fit the dynamic ‘piston’ seal. The dynamic seal uses a single EM010 (10 mm inner diameter and 2 mm cross section) O-ring in Viton/FKM without a back-up ring. The uppermost section of the apparatus is held in place by the actuator carrier. This large titanium carrier is threaded directly onto the top platen. It has four windows, which allow access for wires and components located above the top piston. Its role is to hold the hydraulic actuator, which delivers axial load to the top piston.

The top piston is capped by the top piston cap, which facilitates access of heater power cables and thermocouple wires, as well as pressurized pore fluid through the top piston (Figs. 1 ▸ and 2 ▸, and the supporting CAD drawings). The pore fluid is sealed by two O-rings, creating a circumferential pressure seal, around the piston’s end. The connection to the pore fluid bore of the top piston is made via a 1 mm hole drilled at right angles to the long piston axis. A 1/16" stainless steel tube was brazed into the cap and fitted with a 1/16" Swagelok union. On top of the top piston cap, a tell-tale is inserted to work with a linear variable differential transformer (LVDT, also linear variable displacement transducer) to measure the piston displacement and calculate the sample strain. The LVDT is clamped in an external aluminium ring fixed at the top of the actuator carrier. On top of the top piston cap, the connection with the actuator is made using a slotted spacer, which prevents mechanical loading of the thermocouple and heater cables.

The axial load delivered to the sample via the top piston is generated by an Enerpac CST 40132 single acting actuator cylinder threaded into the actuator carrier. The maximum stroke is 13 mm and the maximum delivered load is 39.2 kN. A calibration of actuator load versus applied ram pressure is given in Section S5 of the supporting information and Fig. S3. A stiffness calibration, necessary for deformation studies, is given in Section S6 of the supporting information and Fig. S4.

**Figure 2:**
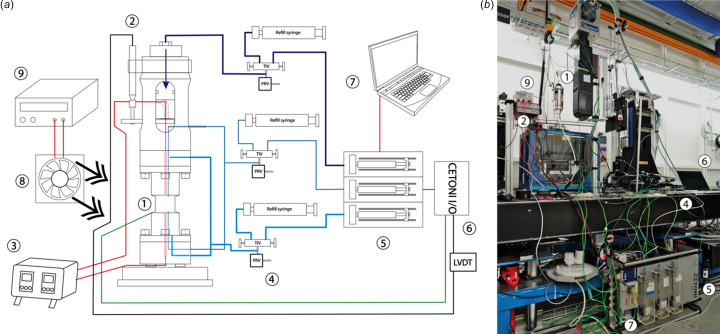
(*a*) Cartoon schematic of the layout and connection between Heitt Mjölnir and the high-pressure syringes, LVDT (black), power cables (red), thermocouples (green) and fluid (blue) connections for routine operation. (1) Type-K thermocouple(s) fixed on the pressure vessel. (2) Linear variable displacement transducer. (3) Temperature controller with two channels for top and bottom cartridge heaters (red shaded areas inside the pressure vessel). (4) Three fluid manifolds composed of Top Industrie valves (TIV) to disconnect the cell from the pump and refill (refill syringe) without dropping the cell pressure. Each fluid line must be equipped with a proportional pressure relief valve (PRV). Connections to PEEK tubing are via Swagelok 1/16" bulkhead unions. (5) Three Cetoni Nemesys high-pressure syringe pumps provide independent fluid delivery for the confining pressure (sky blue), pore fluid pressure (blue) and axial load via the hydraulic actuator (purple). (6) CETONI I/O module. (7) Laptop PC with *QmixElements* software to control the high-pressure pumps (5) and log experimental data (7). Solid lines indicate external cable or tubing while dotted lines indicate their continuation inside the cell. (8) Intel cooling fan for the pressure vessel and its associated 12 V DC power supply (9). (*b*) Heitt Mjölnir in position and set up for operation on the rotary table of the TOMCAT beamline at the Swiss Light Source. Letters correspond to those in (*a*). The Heitt Mjölnir rig is fixed on the rotary table at approximately 1400 mm height above floor level.

**Figure 3:**
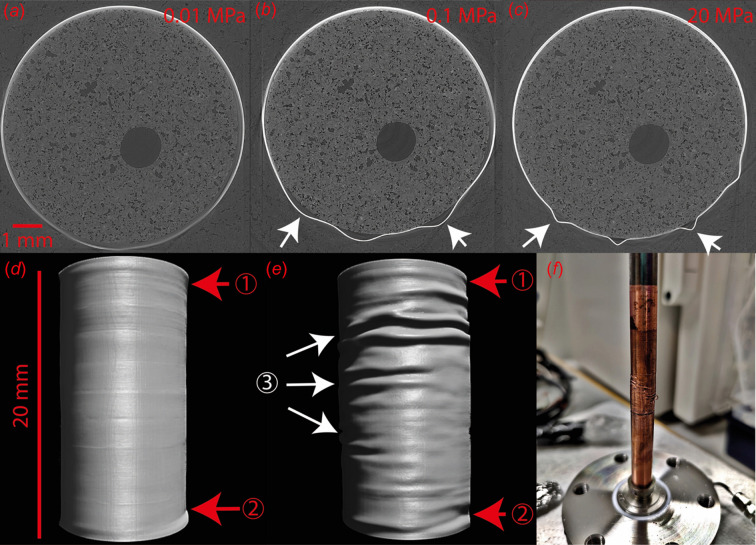
Copper jacket deformation during high pressure and temperature runs. Panels (*a*), (*b*) and (*c*) are 2D slices acquired at the I12 JEEP beamline of Diamond Light Source during a basalt carbonation experiment at confining pressures of 0.1, 1 and 20 MPa, respectively. Panels (*d*) and (*e*) are 3D renderings of the copper jacket surface during a gypsum dehydration and deformation experiment at the TOMCAT beamline of the Swiss Light Source. The axial shortening was 1.18 mm in (*e*) (6% of strain). Arrows (1) and (2) indicate the position of the sample/piston interfaces. Wrinkles and ridges (3) are due to the shortening of the sample under differential load during dehydration. (*f*) Photograph of the jacket after the experiments.

The fluid pressure for the actuator axial load, the confining and the pore fluid pressure are all delivered by high-pressure Cetoni Nemesys HP-syringe pump modules (see detail in Fig. 2 ▸ and Section 3). These are remotely controlled by using *QmixElements* software which offers control on the flow rate and PID control of the pump pressure. Each pump is connected to a manifold (using Swagelok and Top Industrie unions and valves) which facilitates refilling the pump without disconnecting the cell. Flexible fluid lines of 1/16" PEEK tubing enable cell rotation. All commercial fittings and tubing used have pressure specifications higher than the targeted experimental range for this apparatus.

In Heitt Mjölnir, the heating system is internal and located within the cell pistons, using Watlow Firerod cartridge heaters (1/8" diameter) with 75 or 100 W maximum power capacity. The heaters use Watlow EZ-ZONE controllers driving Watlow DIN-A-MITE solid-state power controllers. The two cartridge heaters have their own internal type-K thermocouple. Sample temperature cannot be measured directly in the current design, but it has been calibrated using modified top and bottom pistons allowing temperature profiling inside the pressure vessel and sample (see Sections S1 and S4 and Fig. S2 of the supporting information).

### 2.1. Modification of the basic design

Heitt Mjölnir’s components can be modified, exchanged and replaced to cater for bespoke research questions. Operations beyond the limits and use of materials that differ from those recommended here require safety testing with respect to loads, strengths and temperature management. This cell has been designed to use thermally resistant liquid pressure media only (water is not a suitable confining pressure medium above 373 K). The maximum operating temperature is fixed at 573 K in the sample for the duration of a week, as tests show that several components start to degrade at higher temperatures. Care must be taken to limit circulation of the confining fluid in the cell, because heating the pressure vessel above 393 K has the potential to impair its mechanical properties. An efficient cooling system must be used to cool the pressure vessel. The cell is designed to operate with an actuator in place and applying a minimal load to ensure sample and pistons remain in close contact. The use of relevant pressure relief valves (and their test prior to operation) on confining and pore fluid pressure lines is mandatory for user safety.

## 3. Operation of the cell and data acquisition

### 3.1. Sample preparation

Sample preparation is crucial. Cylindrical samples must have a diameter of 10 mm and a length of 20 ± 2 mm (see supporting Section S1). The upper piston movement allows some length variation; sample diameter is fixed by the inside diameter of the copper jacket, and its tolerance.

Sample cylinders are prepared using a water-flushed diamond core drill. Depending on the material, cores drilled to larger diameters can be turned down/ground on a lathe. The cutting and polishing of two flat and parallel surfaces on the cylinder ends are important. One can use a diamond impregnated disc mounted on a lathe tool-post or polish the sample on fine-grained sandpaper using a custom-made sample holder on a flat surface (supporting Section S1).

### 3.2. Cell assembly and experiment preparation

The assembly of Heitt Mjölnir follows a strict protocol. Full guidance is included in Section S3 with detailed photographs for each step.

The cell is assembled from the bottom up, starting from the bottom platen, the baseplate and breadboard. The copper jacket is slid down the bottom piston, then the sample and separate top piston are inserted in the jacket successively. The copper jacket is sealed against the piston using UniLok heat-shrinkable memory metal rings overlapping the top and bottom ends of the jacket. The graphite thermal bonding sleeve must be inserted over the copper jacket before the second UniLok ring is located on the top piston. During positioning of the copper jacket and UniLok seals an oleophobic surface treatment is applied to the pistons and jacket to aid the sealing between the piston and jacket (Section S2.2). Once this sample pack is assembled and sealed, the remaining parts of the cell, bottom to top, are successively slid over it.

The cell is fixed on an aluminium baseplate, which has several functions: (1) it provides a link to the M6 optical breadboard which itself is used to attach the rig to the beamline rotary table, (2) it thermally isolates the cell from the rotary table through PTFE spacers, and (3) it permits installing strain relief of the pressure tubes, power and signal cables. Finally, the baseplate is designed to protect the rotary table during connection and disconnection of the fluid-filled PEEK tubing.

Heater connections are made with wires rated to 10 A at 230 V. The heater-cable and cable to power control are both connected using three-pin cable-mounted IP68-rated connectors with twist locks. All pressurized fluid tube connections from the cell to the pumps are made with a 1/16" PEEK HPLC tube with a bore of 0.01". The connection between the PEEK and the cell body is made with short 1/16" stainless steel tubes (with a bore of 0.01") which permit a tighter bend radius than PEEK tubes and facilitate strain relief for the cell connections. Both top and bottom paths of the cables and capillaries must clear the X-ray beam path to avoid imaging artefacts. Additionally, all fluid and electric connections must be strain-relieved to allow 360° cell rotation without wearing out cables or pulling on fluid connections during the, typically, hundreds of rotations during an experiment. The heater cables and fluid tubing coming from the bottom of the cell are relieved via cable ties fixed to the baseplate and the M6 breadboard. On the top side, wires and tubes are secured to the actuator carrier and suspended on a gantry positioned above the cell (see Figs. 1 ▸ and 2 ▸).

For the experiments, depending on the pressure and temperature conditions targeted, different confining fluids can be used. For moderate temperatures (<373 K) deionized water is a suitable confining fluid. It leaves the cell components easy to clean, does not clog the tubing and is not haza­rdous. For any operation involving high temperatures (>373 K), silicone oil is preferred. Caltherm S1050, Syltherm 800 and Caltherm 400 have all been found to be suitable.

The assembly of the cell and location on the beamline takes experienced users less than 2 h. However, curing of the oleophobic coating applied to the pistons, where the copper jacket seals to them, can take several hours. At room temperature, the curing process takes 5 h but can be speeded up with a hot-air dryer. Multiple sets of bottom platen and top pistons allow the preparation of sample packs in advance, ready to be assembled.

### 3.3. Cell operation

Operating the rig requires real-time data acquisition, which is enabled by a Cetoni I/O module logging the LVDT and thermocouples into *QmixElements* software on a single laptop (and log files) (Fig. S6). Data from Cetoni Nemesys HP pump modules are also simultaneously logged by the *QmixElements* software.

To load the cell to the desired conditions, the confining pressure, axial actuator pressure, pore fluid pressure and cell temperature are successively increased to the target values. The confining axial actuator and pore fluid pressures are increased stepwise manually or using a PID control loop. This allows different axial loading strategies: (*a*) constant stress (creep or hydro­static) experiments, with stress values monitored and kept constant during the run, (*b*) the application of a specific loading or strain rate or (*c*) stepwise loading to a series of constant stress states. Continuous strain rates as low as 1.5 × 10^−7^ can be reached with the Cetoni Nemesys high-pressure pumps. The bulk sample strain can be determined directly from LVDT data (with prior calibration) or can be determined *post mortem* by measuring distances from radiographic or tomographic images (*e.g.* distance between pistons).

If the actuator is used to lock the top piston in place, without application of hydraulic pressure to the actuator, Heitt Mjölnir can be used for static fluid–rock interaction experiments, as in a thermal reactor, and fluid flow experiments. This configuration restricts axial displacement, and the axial load remains constant or may change due to volume changes related to chemical reactions in the sample. On the other hand, the axial actuator can be set to end-load the sample to match the confining pressure.

Finally, the pressure and injection rate of the fluid (in the pore fluid channel) in contact with the sample can be controlled. Slight adaptation of the pump system (inlet and outlet pumps, tee unions, manifolds, *etc*.) allows control of the residence time of fluids in a porous sample, sampling the fluid after its interaction with the sample or use several fluids (liquids and gas) for multiphase fluid flow experiments in different stress states.

## 4. Calibration of the apparatus: mechanical testing (pressure, stiffness), heat distribution and temperature gradients

Heitt Mjölnir was tested in offline experiments using both the prototype and the final versions to better understand its mechanical behaviour, heat distribution and fluid transfer in the cell. These tests demonstrated the stability of the apparatus for a duration of at least a week at maximum temperature.

The actuator fluid pressure versus resultant axial load and resulting sample pressure were calibrated using a 50 kN load cell (see Section S5 and Fig. S3 for details). We found the relationship to be linear above axial loads of 0.24 kN (1 MPa of actuator fluid pressure) up to 40 kN (resulting in sample axial pressures >500 MPa). We further quantified the stiffness of the apparatus using uniaxial and triaxial compression of a grade 5 titanium dummy sample (see Section S6 and Fig. S4). The stiffness value is 7.1 GPa and 7.3 GPa in uniaxial and triaxial tests, respectively. Heitt Mjölnir behaves in a repetitive manner and only has a small hysteresis during unloading. The equations to retrieve sample strain from the apparent strain, necessary for rock deformation studies, are given in Section S6. Both load and stiffness calibrations given in this manuscript used the recommended sample dimensions (10.0 mm diameter by 20.0 mm length).

Additional offline testing measured the temperature distribution inside and outside the rig. The temperature gradient within the sample was measured to be below 0.5 K mm^−1^ (Section S4 and Fig. S2). A setpoint of 623 K for the heating cartridges yielded a sample temperature of 584 K in the sample centre with less than 373 K on the outside surface of the air-cooled pressure vessel. The temperature of 623 K at the piston hot spots corresponds to the maximum operating temperature capability for silicone oils and defines the effective maximum temperature limit of the device.

A discussion on accuracy, precision and errors of thermal and mechanical parameters inferred from the rig is provided in Section S8.

## 5. Examples of deployment and data acquisition

### 5.1. Setup and deployment at synchrotron imaging beamlines

**Figure 4:**
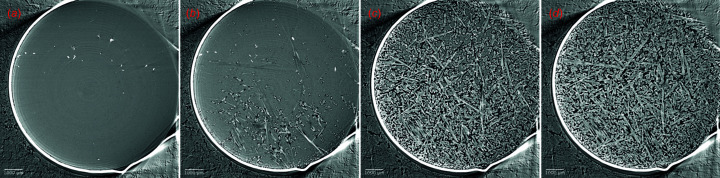
Time evolution of calcium sulfate reactants and products through a gradual temperature increase during a triaxial deformation experiment at the TOMCAT beamline (SLS). 2D slices show the progressive transformation of gypsum (dark grey) (*a*) to gypsum + bassanite (light grey) (*b*), bassanite (*c*) and anhydrite (light grey) (*d*). The dark moats surrounding bassanite and anhydrite needles are fluid-filled porosity. The bright unreactive phase is celestite. Tomography volumes were acquired with a sample–detector distance of 2500 mm. The scale bar at the bottom left of the image is 1 mm long.

Heitt Mjölnir was first deployed on the TOMCAT beamline X02DA of the Swiss Light Source (SLS), Villigen, Switzerland, in June 2021. It was deployed again at the same beamline several times in 2021 and 2022. The apparatus has also been deployed at beamline I-12 Joint Engineering, Environmental and Processing (JEEP) at Diamond Light Source (DLS), Didcot, UK, in 2022, and at Synchrotron SOLEIL’s (CNRS–CEA Paris-Saclay) PSICHÉ (Pression Structure Imagerie par Contraste à Haute Énergie) beamline, Saint-Aubin, France, in June 2023. Examples of data acquired during these beamline visits are presented in Figs. 4 ▸, 5 ▸ and 6 ▸. They highlight the capabilities of the device, its versatility and flexibility for deployment at various beamlines, as well as sufficient transparency at these tomography beamlines powered by a third-generation synchrotron source. In the following section, we provide information about the beam type and setups to ease and facilitate the setup of Heitt Mjölnir at these imaging beamlines. These instructions are useful for beamline staff to establish conditions under which high-quality images can be obtained (see Figs. 4 ▸, 5 ▸ and 6 ▸ for comparison of results at different facilities).

**Figure 5:**
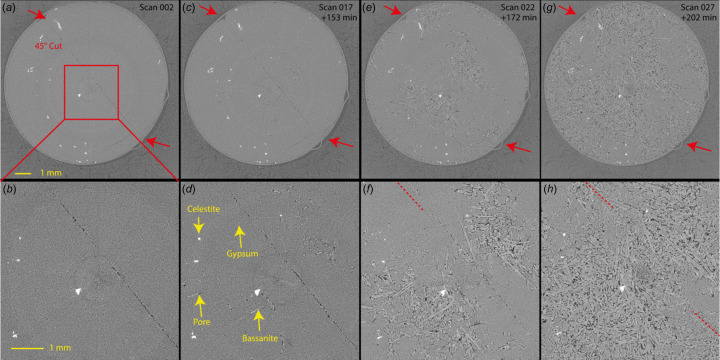
Time evolution of a pre-cut (45°) gypsum sample during its partial dehydration while under triaxial conditions at the TOMCAT beamline (SLS). Couples (*a*, *b*), (*c*, *d*), (*e*, *f*) and (*g*, *h*) show the gradual dehydration with a view at the sample scale and local scale on the top [(*a*), (*c*), (*e*) and (*g*)] and bottom [(*b*), (*d*), (*f*) and (*h*)] rows, respectively. The scan interval is 6 min [30 min between (*c*), (*e*) and (*g*)]. The position of the cut plane at 45° is highlighted by the red arrows and dashed lines when poorly visible. Phases present are gypsum (dark grey), bassanite (light grey), pores (black) and celestite (white) and are indicated by the yellow arrows. Tomography volumes were acquired with a sample–detector distance of 500 mm.

**Figure 6:**
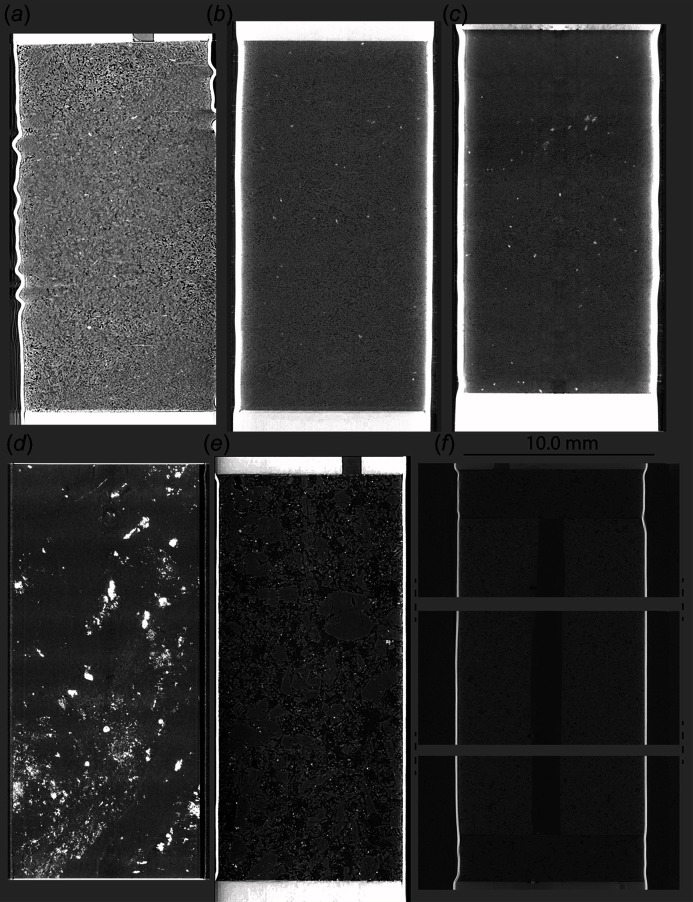
Vertical cross sections of diverse types of samples and experiments acquired at the TOMCAT, I12 JEEP and PSICHÉ beamlines. (*a*) Dehydrated gypsum sample into anhydrite (TOMCAT). (*b*) Gypsum dehydration into bassanite (PSICHÉ). (*c*) Bassanite rehydration into gypsum (PSICHÉ). (*d*) Serpentinite from Shiraga belt in Japan (TOMCAT). (*e*) Picrite from Faroe Islands (TOMCAT). (*f*) Porous basalt from Iceland (DLS). Space was left in between the subvolumes because they do not perfectly overlap. Tomography volumes at the TOMCAT beamline were acquired with a sample–detector distance of 190 mm (*d*) and 2500 mm (*a*, *e*).

Time-resolved µCT data at TOMCAT were acquired using filtered white-beam radiation. The SLS storage ring has an electron beam energy of 2.4 GeV and a beam current of 400 mA. The white X-ray beam is provided by a 2.9 T super-bend magnet and was filtered with 20 mm of glassy carbon, 350 µm of molybdenum and 12 mm of borosilicate glass filters. We used a 150 µm LuAG:Ce scintillator from Crytur, Czech Republic. This heavily hardened X-ray beam had a peak energy of 59 keV. Transmission through the cell was about 11% of the incident radiation with the filters in place (including the gypsum sample and a copper jacket with a wall thickness of 70 µm). The extensive simulation of the beam energy spectrum and X-ray flux for the beamline and Heitt Mjolnir is given in Section S7 and Fig. S5. While most of the flux from the incident beam, predominantly at lower energies, is absorbed by the filters, the amount of transmitted radiation at higher energies was sufficient to achieve scanning frequencies of a few minutes, compatible with the geological processes investigated in our experiments. The GigaFRoST camera system (Mokso *et al.*, 2017 ▸) was used in conjunction with a high-numerical-aperture macroscope with a four-fold magnification (Bührer *et al.*, 2019 ▸). The native pixel size of this detection system is 2.75 µm. Different sample–detector distances were investigated from 190 mm [Fig. 6 ▸(*d*)] to 500 mm (Fig. 5 ▸) and 2500 mm [Figs. 4 ▸, 6 ▸(*a*) and 6 ▸(*e*)], where best imaging quality was obtained for phase retrieval using the algorithm based on the work of Paganin *et al.* (2002 ▸). With a source–sample distance of about 25 m at the beamline and a propagation distance of 2500 mm, the resulting effective voxel edge size is 2.5 µm in the collected images.

Scan durations depend upon the mode of data acquisition. Fast scans were acquired using a 180° cell rotation. These scans were performed with 2000 projections, an exposure time of 28 ms and yielded a temporal resolution of about 1 scan per minute. The effective field of view at TOMCAT is 2.0 mm high (limited by the extent of the X-ray beam) and 5.0 mm wide. Given the size of the sample, we found this configuration was suitable for region-of-interest scans but limited the amount of contextual information acquired on the sample such as bulk strain, fracture extent or sample-scale fluid pathways. For these reasons, when high temporal resolution is not needed, a 360° cell rotation with an off-centred rotation axis was preferred. The duration of each scan was 2 min with 4000 projections and an exposure time of 28 ms. These scans provided the ability to acquire the full 10 mm diameter of the sample with a vertical field of view of 2.0 mm. A series of 11 overlapping vertical translations was necessary to provide a full sample volume tomography. Consequently, the combined high-quality imaging of the full sample required about 25 min (2 min per 360° scan, vertical translations, and flat and dark fields). The data volume acquired was 132 GB for a full sample volume (12 GB per scan), requiring careful preliminary considerations for data processing, management and storage.

At I12 JEEP, the cell was mounted in the I12 Experimental Hutch 1 with sample table configuration 1. The DLS storage ring has an electron beam with an energy of 3 GeV and a beam current of 300 mA. Polychromatic X-rays are provided by a 4.2 T superconducting wiggler and subsequently monochromated to 70 keV, not using any additional filters, producing a slightly divergent beam on the sample. Their high-resolution camera with optical module 3 was used to produce a reconstructed voxel size of 3.24 µm. 2000 projections of 11 ms exposure were acquired over a 360° rotation, resulting in scan durations of about 4 min (including camera readout). The sample–detector distance for these scans was 500 mm.

At PSICHÉ, Heitt Mjölnir was deployed in the first experimental hutch (optics hutch) and using the polychromatic imaging mode (fast or high-energy filtered white-beam tomography) of the beamline. The SOLEIL storage ring has an X-ray beam energy of 2.75 GeV and an electron beam current of 450 mA. The sample was irradiated with a white X-ray beam coming from a 2.1 T multipole wiggler and was filtered with 1.5 mm aluminium, 5.6 mm copper and 0.6 mm lead filters for an average energy of 90 keV. We used a 250 µm LuAG:Ce scintillator and a Hasselblad tandem 2.1× magnification lens. The beam energy was tested at 67 and 90 keV and transmission was estimated to be above 8% at 67 keV with the filters in place (with gypsum samples and a copper jacket wall thickness of 200 µm). The sample–detector distance on this setup was found optimal at 300 mm, where longer propagation distances introduced blurring due to beam divergence. The detector used was a Hamamatsu ORCA Flash 4.0 and the effective voxel edge length was 2.84 µm. Scans were acquired using 5700 projections with an exposure time of 15 ms and realized with a 360° cell rotation with the rotation axis offset to extend the horizontal field of view. The beam size was 5.8 mm × 2.9 mm, and a single reconstructed volume comprised 10.9 mm × 10.9 mm × 2.9 mm. A total of seven to eight overlapping blocks were necessary to image the full sample. The combined imaging of the full sample required about 18 min (2 min per 360° scan, vertical translations, and flat and dark fields). Single scans represent a total of 32 GB per reconstructed volume (16 bits) and 255 GB for a full sample volume.

### 5.2. Examples of 4D experiments with 4D microtomography

Various types of experiments were performed during multiple deployments at the three beamlines. In this section, we describe the different experimental procedures for each experiment type to illustrate the possibilities offered by this rig.

#### 5.2.1. Mechanical tests

Heitt Mjölnir can conduct various types of mechanical tests. Cold compression and mechanical failure experiments were performed on serpentinite and gypsum samples at the TOMCAT beamline. In these runs, the sample was first loaded to a confining pressure between 15 and 25 MPa. Differential stress was incrementally applied through a staircase ramp with small pressure increments on the actuator. For each series of vertical scans, the axial load and confining pressure were kept constant until the failure of the rock samples. These particular tests, which do not require heating, can be performed using a silicone/polymer jacket rather than the copper jacket discussed elsewhere.

Mechanical tests can be conducted under high-temperature conditions, as illustrated in Figs. 4 ▸ and 5 ▸. For instance, dehydration tests on intact and pre-cut gypsum samples (with a 45° angle) were carried out under a constant differential stress. In these experiments, the samples were initially subjected to a confining pressure, followed by a monitored, constant differential stress. The pore fluid pressure was then increased and maintained throughout the experiment. Concurrently, the temperature was incrementally raised until it reached the reaction target. A staircase ramp of differential stress at a specific temperature could also be applied. During experiments, tomography data sets can be collected at regular intervals or specific time steps, offering flexibility in data acquisition frequency while maintaining control over pressure and temperature.

#### 5.2.2. Metamorphic reactions

Heitt Mjölnir was primarily developed to investigate thermo-hydro-chemo-mechanical (THCM) feedbacks during fluid–rock interactions and low-grade metamorphic reactions. Such reactions are extremely common in various fluid-rich environments such as the seafloor, in subduction zones or faults and thrusts zones within the continental crust. With its heating capacity, Heitt Mjölnir enables the study of mineral reactions occurring at the conditions of the zeolite, lower greenschist and hornfels facies. The low temperature gradient within the sample (Fig. S2) facilitates achieving homogeneous reactions and minimizing temperature effects. Additionally, Heitt Mjölnir can be used as a reactor to study reactions under isostatic pressures or with differentially stressed states.

Examples of dehydration reactions of gypsum (CaSO_4_·2H_2_O) samples into bassanite (CaSO_4_·0.5H_2_O) and anhydrite (CaSO_4_) are provided in Figs. 4 ▸, 5 ▸ and 6. These samples were prepared from a polycrystalline block of Volterra alabaster. The loading procedure followed a similar approach to rock deformation experiments, first involving the application of confining pressure up to 20 MPa, and then followed by the application of differential stress (from hydro­static to >30 MPa) and pore fluid pressure (5 MPa). The temperature was initially increased to 398 K to observe the gypsum-to-bassanite dehydration, and subsequently raised above 498 K to observe the complete dehydration of bassanite into anhydrite (Fig. 4 ▸).

Recently, experiments have been expanded into rehydration reactions in the gypsum–bassanite–anhydrite system (Fig. 6 ▸). After initial dehydration steps, the temperature is decreased to reverse the reaction. At this stage the fluid chemistry, temperature or stress conditions can be changed to study their impact on the reaction. Examples of such experiments, displaying pore healing and closure, conducted at the PSICHÉ beamline are provided in Fig. 6 ▸.

#### 5.2.3. Fluid–rock interaction and fluid flow experiments

Heitt Mjölnir allows the study of fluid–rock interactions in 4D and is ideal for operation at conditions relevant to subsurface resources and notably geothermal fields, where temperature often exceeds 473 K at shallow depths.

The control of pore fluid pressure allows mechanical testing of reservoir rock at various effective pressures and enables imaging of hydro­fractures at the grain scale. Different fluids can be introduced into the sample to explore chemical interactions between the fluid and the porous host rock. These interactions encompass such processes as dissolution, precipitation and mineral replacement reactions. The device’s design permits independent control of the pore fluids in the top and bottom pistons. This feature can be used with different pump geometries to study single and multiphase fluid flow in the samples or quantify permeability with pressure difference or sinusoidal fluid pressure waveforms. To ensure uniform fluid access to the sample interface, porous frits can be used at the sample/piston interfaces [see Fig. 6 ▸(*f*)].

Two examples of fluid–rock interaction experiments using Heitt Mjölnir as a flow cell reactor are given in Fig. 6 ▸. This experiment studied basalt carbonation, simulating conditions relevant to geological carbon storage. A cylindrical sample of Icelandic basalt was used with a central borehole of 1.5 mm diameter to facilitate continuous flow through the sample. The injected fluid consisted of a solution of sodium bicarbonate in deionized water. The sample was first compressed with 20 MPa confining pressure and a pore fluid pressure of 10 MPa. The temperature was gradually increased to 523 K while maintaining a solution flow rate of 10 µl min^−1^. A similar experiment was carried out using an intact cylinder of low-porosity porous picrite from the Faroe Islands.

## 6. Conclusions

In this paper, we present the design concept, technical specifications and operating procedures for Heitt Mjölnir, an internally heated triaxial apparatus for 4D synchrotron microtomography. This internally heated rig works with a sample of 10 mm diameter by 20 mm length with a confining and pore fluid pressure range of <30 MPa and 500 MPa axial load, at sample temperatures up to 573 K. The design of the rig ensures low thermal gradients within the sample while maintaining a good transparency to X-ray beams. This manuscript provides detailed guidelines for assembly and operation of the apparatus at imaging beamlines together with calibrations and estimates of device precision.

The rig, when combined with the appropriate imaging arrangements, produces high-quality data with spatial and temporal resolutions suitable for imaging a wide range of dynamic processes at both grain and sample scales.

It enables mechanical testing under diverse conditions relevant to the subsurface. It allows the exploration of physico-chemical-hydraulic feedbacks during metamorphic reactions, at conditions up to the top of greenschist and hornfels facies. The design also enables studies in the areas of mineral resources and subsurface storage, including radioactive waste repositories and CO_2_ sequestration.

## 7. Related literature

The following reference, not cited in the main body of the paper, have been cited in the supporting information: Klementiev & Chernikov (2014 ▸).

## Supplementary Material

Sections S1 to S8, Tables S1 and S3, Figures S1 to S6. DOI: 10.1107/S1600577523009876/vl5015sup1.pdf

Flux calculation files (HTML and Jupyter notebook). DOI: 10.1107/S1600577523009876/vl5015sup2.zip

Merged part drawings in PDF. DOI: 10.1107/S1600577523009876/vl5015sup3.pdf

CAD and 3D models for Heitt Mjolnir drawings. DOI: 10.1107/S1600577523009876/vl5015sup4.zip
