# Supplementary material for: Heitt Mjölnir: a heated miniature triaxial apparatus for 4D synchrotron microtomography
Source: J Synchrotron Radiat. 2024 Jan 1;31(Pt 1):150–61. doi: 10.1107/S1600577523009876 (PMC10833432; doi:10.1107/S1600577523009876)
Supplement: Supplementary file 1 [file s-31-00150-sup1.pdf]

JOURNAL OF SYNCHROTRON RADIATION

Volume 31 (2024)

Supporting information for article:

Heitt Mjölnir: A heated miniature triaxial apparatus for 4D synchrotron microtomography

Damien Freitas, Ian B. Butler, Stephen C. Elphick, James Gilgannon, Roberto E. Rizzo, Oliver Plümper, John Wheeler, Christian M. Schlepütz, Federica Marone and Florian Fusseis

Dimensions and variable values in the publication/supplementary information are given with SI units, except for some cases, where imperial units were used when the components supplied by the manufacturer had imperial dimensions (e.g., parts, tubing, heating cartridges etc.).

S1. Technical drawings for Heitt Mjölnir

Technical drawings are provided as pdf files and Autodesk Inventor CAD format with the extensions .dwg and .ipt.

The list of drawings is given below, their numbers refer to their reference on the bill of material given in the Fig. 1 of the manuscript. Drawings of commercially available components are not supplied, except where those components have been modified. Numbers 23 to 27 (included) correspond to additional parts designed to help assemble the cell and prepare the samples (see supporting information S2). They are not mandatory to operate the cell.

Notes on specific parts like “breaking sharp edges” and putting a “bevel or radius on other edges” should be carefully considered because these are critical to the functionality of the kit. This prevents wear on wires and ease parts manipulation during assembly, operation, and disassembly.

Table S1 List of technical drawings.

1) Thorlabs M6 Breadboard (MB1015/M)
2) Baseplate
3) PTFE Spacers
4) Bottom Platen
5) Pressure Vessel
6) M8 x 20 Hex screw **(no drawing, drawn in 4 and 7)**
7) Top platen
8) Actuator Carrier
9) LVDT Holder
10) Enerpac CST40132 40 kN Actuator **(no drawing)**
11) Spacer
12) LVDT Tell-tale
13) Sample **(no drawing)**
14) Metal Jacket
15) Thermal Bond (graphite sleeve)

16) UniLok ® Rings (AHM 10460.193-0386P) **(no drawing)**
17) Internal PEEK Baffle
18) External PEEK Baffle
19) Top Piston
20) Seal Retainer
21) Seal Retainer Nut
22) Top piston cap
23) 10 kN Actuator Adapter
24) Piston Guide
25) Unilok Ring Installer
26) Sample polishing guide
27) Graphite shaver
28) Actuator piston cap
29) Assembled cell

S2. Details on the manufacture and construction notes:

In this section, we provide essential additional information related to the construction and operation of Heitt Mjölnir, which goes beyond that provided in the main text of the paper.

S2.1. Sample pack design

The dimensions of the sample pack and its design are a function of the pressure and temperature conditions targeted for the rig. The internal heating system required to reach high temperatures imposes important design requirements. The diameter of the *pistons* and the *sample* were selected to be 10 mm, in order to insert a ⅛" diameter cartridge heater and a 1 mm pore fluid hole in the *pistons*. Smaller sample/piston diameters are not possible because they would make the titanium wall thickness between the two holes too thin and would not meet strength requirements to transmit the axial load to the sample.

The manufacturing of the *pistons* represents a technical challenge and we turned to specialist workshops for these jobs. Drilling in grade 5 titanium is difficult due to the work hardening of the material during machining. The drilling lengths (>100 mm) for the 3.2 and 1 mm diameter bores in the *top piston*, and the tight tolerances associated, demand specific machining techniques which may not be available in a normal mechanical workshop. The 3.2 mm bore was machined by gun-drilling techniques for a precise diameter and flat-end finish. Gun drilling provides the high-quality finish required for thermal coupling between the heater and piston. The 1 mm diameter hole was realised with spark erosion (Electrical Discharge Machining, referred below as EDM). The EDM machining step

created a blind hole starting from the piston tip which was later drilled from the side to connect with the *top piston cap* (see technical drawings S1 and Fig. 1). Spark erosion produces a rough finish and is not suitable for the manufacture of the heater hole because the rough finish leads to inefficient thermal coupling between the heating cartridge and the *piston*. For the *bottom piston*, the 3.2 mm hole was drilled in a normal mechanical workshop (shorter drilling depth), but the 1 mm hole also had to be machined with EDM techniques.

Metal jackets need to be tight on the 10 mm diameter *samples* and *pistons*, because the presence of a thin air/confining fluid layer in between the parts can dramatically decrease the efficiency of the thermal bond. Great care must be taken with the fit between the *jacket* and the *pistons* and between the *graphite sleeve* and the *jacket* (tolerances indicated on the technical drawings S1). A snug fit of the *graphite sleeve* over the *jacket* is necessary to avoid any vertical displacement of the sleeve after assembly.

Sealing reliably the jacket on the pistons is a crucial task. The jacket needs to: 1) be thin, 2) seal the sample area from confining fluid, and 3) generate a modest X-ray attenuation. Different materials were tested during the design process: 1) Commercially available copper tubes. These are ready made, standardised, but require bright annealing prior to their use. Their minimum thickness is 0.15 to 0.2 mm (wall) and a source of additional X-ray attenuation (Fig. S5). 2) Manufactured copper tubes, machined from Cu bars (grade C101). Minimum thickness of 70 micrometres (wall) have been obtained but they remained difficult to machine, expensive, and very difficult to make repeatedly. 3) Aluminium alloy tubes. Machined as per Cu, some alloys (SigmaChip A6-T9) were found easier to machine. However, they were found being too hard to make a reliable seal and are also more reactive than Cu to chemical solutions. 4) Silicone tubing, which is commercially available at a low price. Such tubes are easy to use and reliable. Their thermal conduction is poor (favouring large thermal gradients in the sample) and have limited temperature resistance (473 K maximum). Metal seals were favoured for Heitt Mjölnir due to their greater temperature resistance compared to elastomeric polymers. In particular, jackets made from commercially available copper tubes, which had reproducible behaviour and high success rate. Silicone sleeves remain useful for all mechanical tests and calibrations of the apparatus made at room temperature.

The sample cylinder must have its top and bottom surfaces perfectly flat and parallel (see main text). Parts must be tested against each other prior to assembly, as minor adjustments to sample dimensions might be needed.

Finally, the surface of the pistons and the inner surface of the *metal jackets* must be cleaned and polished to ensure a perfect mechanical seal. The inner surfaces were manually polished using a Dremel rotary tool and polishing pads with diamond suspension (with particle size ranging from 12.7 microns to 1 micron) until a mirror polish was observed. The *pistons* were also polished on a lathe using pads impregnated with diamond suspension.

S2.2. Installation and removal of the UniLok® rings

UniLok® rings are single-use metal heat shrinks. Their installation must be done with great care as they can't be moved nor adjusted when shrunk. For their installation, we have machined a "*ring installer*" (drawing S1 N°25) aid to help precise location of the ring (see installation guide below). The rings are shrunk (full clamping force is achieved by heating to 438 K) with a hot air gun set at a temperature of 673 K. Thermochromic paint on the ring indicates when the required shrinkage temperature has been reached.

We found that applying a layer of a combined hydrophobic and oleophobic treatment (Cytonix MicroCure DTO 40; https://cytonix.com/products/microcure-dto-40) which was thermally resistant on the surface of the *piston* and *jacket*-ends was effective to preserve seals during pressurisation and deformation. We apply the coating with a cross hatch pattern on the *jacket-ends* when the sample pack (bottom piston, sample, top piston, and graphite sleeve) is installed and after *Unilok ® rings* were clamped on the *jackets* ends.

To remove the rings after an experiment, the cell must be disassembled in the reverse order (see section S2). The sample pack needs to be immersed in liquid nitrogen for about a minute. The ring can be removed from the jacket manually or with a bearing puller once cold. Appropriate safety procedures must be set in place related to the use of liquid nitrogen and manipulation of cold rig components during this operation.

S2.3. Pressure vessel geometry and its thermal baffling

The *pressure vessel* has been designed to manage the temperature inside the cell efficiently while retaining maximum strength. We selected a tapered geometry where the *pressure vessel* is larger at its base and its top and thinner at its centre. The thinnest part corresponds to the X-ray window where the sample is located. All changes of diameter of the *pressure vessel* wall are made with a smooth radius of 2 mm to reduce the focus of stress at these locations. This geometry allows having the thinnest possible *pressure vessel* wall with 5 mm thickness in sample zone (31 mm OD-21 mm ID), which limits the X-ray beam absorption. The larger diameter wall sections have a thicker wall that brings rigidity to the vessel and more space inside the vessel for the thermal baffling.

Inside the *pressure vessel*, a long cylindrical *internal PEEK baffle* is inserted. This baffle is continuous from vessel edges to the sample and is the only insulation baffle in the central volume of the *pressure vessel*. Its role is crucial as it protects the *pressure vessel* wall from the high temperatures in the sample area. PEEK was selected for its high temperature resistance, modest thermal conductivity, and high transparency to X-rays. The large diameter sections of the *pressure vessel* allow the use of a secondary stage of *PEEK baffles (external)* which protects the *pressure vessel* from the hotspots located in the pistons. These baffles are in direct contact with the *pressure vessel* wall, in addition to their supplementary thermal insulation, they also limit the volume of confining fluid in the vessel. These

baffles have a bevel on the inside lip of the baffle to facilitate oil path from the fluid ports (in top and bottom platen) to the internal chamber.

S2.4. Confining fluids

Several confining fluids were tested for the operation of Heitt Mjölnir. We found that temperature resistant silicone oils: Caltherm® S1050, Syltherm® 800, and Caltherm® 400 worked over the whole P-T range of the rig. These substances are identified as non-hazardous and have a very low toxicity making them suitable for handling and deployment at various facilities. As indicated in the manuscript, deionized water can be used for low temperature application but is not suitable for experiments above 373 K.

Care must be taken when working with oils, as they are difficult to clean. Inner parts of the cell, syringes and HPLC tubing, and connectors must be cleaned regularly. We found that flushing the whole fluid delivery circuit several times with isopropanol and acetone provided a satisfactory removal of residual oil and prevented clogging of the tubing.

S2.5. Dynamic and pore fluid pressure seals

To seal the hot confining fluid, Heitt Mjölnir uses different solutions depending on the nature of the contacts between the parts and their relative function. While BS032 PTFE O-rings are used for the static seals between the *pressure vessel* and the *platens*, the *top piston*, which can move during the experiment, requires an elastomeric seal. The confining fluid is sealed on the uppermost part of the *top platen* with an EM010 (10 mm inner diameter and 2 mm cross section) O-ring in Viton/FKM compressed circumferentially against the *top piston* and without a back-up ring. To accommodate axial displacement, the seal is maintained in position by a phosphor bronze *seal retainer*, which acts as a linear guide for the *top piston*. The *seal retainer* is driven precisely to its emplacement by a *threaded nut* in grade 5 titanium, which is installed on top of the *top platen*. The machining of the O-ring groove, *seal retainer and threaded nut* must give a sliding fit to the piston and allow its smooth displacement when the nut is in position but unlocked by a ¼ of a turn.

The pore fluid pressure line is sealed and exits the *top piston* upper end via the *top piston cap*. This part vertically connects the *top piston* with the *actuator* and the 1 mm diameter hole to the pore fluid line on its side. It allows the power and thermocouple cables from the cartridge heater to exit the piston from the 3.2 mm gun-drilled hole. This part is made of stainless steel and has a complex internal cavity to incorporate two EM010x2 Viton/FKM/FPM O-rings. The two O-rings are located above and below the side exit of the 1 mm hole and seal circumferentially around the piston surface. On the side of the part, the fluid line is connected with a 1/16” stainless steel tube brazed in the stainless-steel body and terminated with a Swagelok fitting. The top section of the *top piston cap* lid has been drilled to pass the top cartridge heater wires and thermocouples. Note the importance of tapers both on the *piston*’s

end and *top piston cap* exit, to prevent damage of the O-ring seals as well as the heater and thermocouple wires.

S2.6. Actuators and low loads experiments

In this manuscript, we use Heitt Mjölnir with a 40 kN Enerpac CST40132 hydraulic actuator. This actuator generates axial pressures up to 510 MPa on the 10 mm diameter sample. These axial loads are adapted to rock deformation studies but may not be suitable for experiments at low loads or requiring finer control on the applied load. For such applications, we designed an *adaptor* part (see technical drawings S1 No. 23) in order to use a 10 kN Enerpac CST10132 actuator with the same actuator carrier. Calibrations for both actuators are given in supplementary Section S5 and Fig. S3.

S2.7. Valves

Heitt Mjölnir is connected to the high-pressure Cetoni Nemesys syringes and pumps via a set of 1/16” PEEK capillary tubing and Swagelok connectors. These two units are linked to a set of manifolds and valves (Fig. 2) for 1) connecting or isolating the cell from the pumps, 2) refill the high-pressure syringes without disconnecting the rig and 3) maintain safety and prevent high pressure build up in each line. For the two first tasks, a set of Top Industrie valves (910.50.10 11/19 rated for 100 MPa) were mounted on each line. For safety reasons, we have added stainless steel high pressure proportional pressure relief valves (PRV) from Swagelok (SS-4R3A). These PRV can be set to different maximal pressures via interchanging their spring kit and precise calibration prior to the experiment. They prevent pressure build-up above a level defined by the experimental requirement and can be set to different opening pressure on each high-pressure line (for example: PRVs can be set open at 25 MPa on the confining pressure, 10 MPa on pore fluid pressure and 5 MPa on actuator ram pressure lines using different springs).

S2.8. Linear variable differential transformer (LVDT)

The LVDT used with Heitt Mjölnir is a RDP GT1000 (RDP Electronics Ltd) with spring return and is operated with an RDP E309 Transducer Indicator which powers the LVDT and conditions the LVDT output for retransmission to the Cetoni I/O device as a 0-5 V signal.

S2.9. Power supply for cartridge heaters

Two ⅛” diameter Watlow Firerod cartridge heaters are powered by a custom power supply and controller unit. This unit is composed of two identical channels (one for each cartridge) and was assembled in an Enclosure for Customizable Electronics (EC2530CR) from Thorlabs. Inside the enclosure, each line was fitted with a Watlow PM6C1FJ-AAAABAA Controller and a DIN-A-MITE Power Controller DB10-24F0-0000. The power cables to the cartridges are connected to the enclosure

via Panel Mount IEC Connector sockets. The assembled unit must have a fuse, be grounded and PAT (Portable appliance testing, see hse.gov.uk for guidance example) by competent authorities prior to use.

The controller unit must be located inside the control room to allow user control while the experiment is running. It requires long power and thermocouple extension cables (10 m) to go from the experimental hutch to the control room. To ease the rig installation and assembly, the heating cartridges were soldered to 1 metre cable extensions and connected to the longer cord to the controller unit with miniature circular waterproof connectors (rated IP68, RS144-0620 and 144-0616). A similar strategy was used for the type K thermocouple monitoring each cartridge temperature, with a connector placed between the extension cables. Both connectors, cords and extension cables must be rated over the current and voltages delivered by the controller unit. We recommend the controller unit to be plugged in a dedicated channel of the control room (with its own fuse) to avoid any disconnection of critical beamline equipment in case of a short circuit, heater failure etc. Such a short circuit could occur due to cable strip and damage during the multiple rotations of the device if not properly adjusted/strain relieved.

S2.10. Pressure vessel testing, vessel lifetime and failure modes

Pressure vessels should be regularly hydraulically tested beyond their operating loads to ensure the users and staff safety during deployments. The apparatus must be inspected and tested in conformity with local pressure safety requirements which vary depending on the country (national HSE guidance) and institution regulation (university, laboratory, synchrotron facilities etc.).

Fatigue during repetitive use and pressure cycling will lead to a decreased performance of the *pressure vessel* through time. The decrease of strength with time is a complex function of the duration spent at pressure and temperature, and providing a number of indicative cycles for a vessel lifetime is not possible. Repeated tests and inspections are however an efficient method to detect any alteration prior to the ultimate failure stage.

We recommend testing the pressure vessel prior to the run series, in a safe environment. Conventionally, pressure tests are conducted hydraulically using an incompressible fluid to remove or substantially reduce the possibility of stored elastic energy which would present a danger during rupture. After these tests the vessel dimensions must still fall within the tolerances indicated in the technical drawings. This testing operation can be made off-line with safety devices (knowing the rig is operated at extreme conditions) such as plexiglass shields to protect operators from any failure.

The weakest point of the device is the X-ray transparent pressure vessel, and we recommend keeping a track of the original dimensions of the parts and the number of experiments each vessel has performed with their respective conditions (pressures, temperature, and durations). As stated in the manuscript, we recommend always using the cell with a thermocouple fixed on the outer surface of the *pressure vessel* to monitor its efficient cooling and ensure the vessel is kept below 373 K (ensuring maximum aluminium strength). The failure mode of the *pressure vessel*, if tested with a limited volume

pump driven at low speed (manual pump, high pressure syringes, etc.), will involve yielding, followed by rupture if loading is continued (ductile material). This deformation may lead to the alteration of the flat surfaces at the vessel base/top and induce leaks at the *BS023 O-rings* emplacements. If not leaking, ductile fracturing (subsequent to extensive plastic deformation) is expected. We assume these fractures to form in the vessel centre (where the aluminium is the thinnest) to lead to the leak of the confining fluid, but the shape of the vessel might result in stress concentration in other places. Given the volumes in place, the limited stored energy by the confining fluid (liquid, no gas), the failure of the vessel is not expected to produce anything more than a few drops of confining liquid through opening fissures of the vessel. We also stress that the use of PRV's also prevents any risk of pressure build-up, again limiting the energy release in a failure event.

Table S2 Tabulation of materials.

Material	Grade/Temper	Supplier	Parts
Aluminium	7068 T6	Smiths Metal Centres LTD	Pressure vessel
Aluminium	AW6082-T6511; BS EN755 (H30TF)	Smiths Metal Centres LTD	Baseplate and LVDT holder
Titanium	Grade 5 (AMS4928 & 3TA11) Centreless ground bar H9	Titek UK LTD	Top pistons
Titanium	Grade 5 (ASTMB348)	Titek UK LTD	Platen and actuator carrier
Phosphor bronze		RS components	Retainer
Stainless steel	316/304	Smiths Metal Centres LTD	Top piston cap
UniLok rings	AHM1046-0193-0386	Intrinsic Devices Incorporated	Jacket seals
Copper	CW004A-D; BS EN13601 (C101, HDC101; BS 1432 + 1433) Copper	Smiths Metal Centres LTD	Metal jacket
Graphite	6507	Ceramic Substrates & Components Limited	Thermal bonding sleeve
Polyether Ether Ketone (PEEK)	VICTREX PEEK 450G	Smiths Metal Centres LTD	Baffles
Hydro and oleophobic treatment	MicroCure DTO 40	Cytonix	Seals
PTFE O-ring	PTFE023	Supaseal (UK) LTD	O-rings
FKM/FPM/VITON O-ring	EM010X02V75	Supaseal (UK) LTD	O-rings

S3. Instruction for assembly

Assembling Heitt Mjolnir is a straightforward bottom-up operation. Each operation must be handled with care, particularly for sample jackets, and in the given order. The different steps are illustrated in Fig. S1, and the numbers of the parts are indicated as given on the bill of material given in the Fig. 1 of the manuscript.

1) The first stage requires the *bottom platen* of the cell (4), the bottom cartridge heater, the *aluminium baseplate* (2), *PTFE spacers* (3), and their bolts.
2) Insert the cartridge heater into the *bottom platen* and ensure it is pushed to the end of the hole. Wires must be pulled with great care as the thermocouple wires are very fragile.
3) Insert the bolts and the *spacers* (3) in the *baseplate* (2) and fix the *baseplate* to the *bottom platen* making sure the heater and thermocouple wires are not compressed by the *spacers* (3).
4) Using a polished 9.85 mm diameter *metal guide* (24), slide the *copper jacket* (14) over the *bottom piston* (4). The *jacket* should sit about 20 mm above the base of the *piston* (centre of the cell). Note that both *pistons* (4 & 19) and *jackets* (14) must be polished and carefully cleaned to avoid any imperfections in the contacts.
5) Remove the *metal guide* (24) keeping the *jacket* (14) on the *bottom platen* (4) and insert the sample (13). At this stage, if the sample or the *jacket* are tight on each other and have difficulties to slide, a Vernier height gauge can be used to gauge the height of the sample and piston stack to make sure there is no space left between the sample and the *pistons*.
6) Slide the *top piston* (19) gently into the *jacket* (14). Make sure it is in contact with the sample's top surface. The *top piston* orientation must be selected before insertion, as the fluid port hole located at its top must exit through one of the windows of the *actuator carrier* (8).
7) A first layer of CYTONIX Microcure DTO40 can be applied at the interface between the *pistons* and the *jacket*. Application is made with a thin brush making a cross hatched pattern. The oleophobic coating dries at ambient conditions in about 5h, which can be reduced to tens of minutes if dried in an oven or using a hot air gun. Dried Cytonix is not sticky to the touch and produces a fine, white, powder if scratched with a nail.
8) Once the Cytonix layer has cured, a *UniLok® ring* seal (16) must be installed on the interface between the jacket and the bottom piston. A *ring installer* (design provided in technical file N°25) can be used to hold the ring in its target position. It should be held in a position covering the jacket-piston interface (approximately centred on the contact). The ring is shrunk using a hot air gun, blowing homogeneously across the ring's surface until fully shrunk (at a temperature of about 438 K). Once shrunk, the whole cell must be cooled down before the next operation.
9) Once the bottom seal has been fixed and cooled down, the *graphite sleeve* (15) must be slid over the sample jacket and located at the sample centre. The *sleeve* must be machined to be

tight on the *metal jacket* (14) and hold itself in place. If too tight, a *graphite shaver* (27) can be used to ream the sleeves inside diameter. If too loose, a PEEK or metal spacer can be inserted between the seal and *graphite sleeve* to prevent the sleeve sliding downwards.

10) The *UniLok® ring* seal at the top interface can be now installed, shrunk, and cooled down as detailed in the previous step (8).
11) A second layer of CYTONIX must be applied on the nitinol ring-jacket-piston interface. Application is made as for step 7), making sure the interface is well covered by the surface treatment.
12) Fit a *PTFE BS023* O-ring (A) on the dedicated groove on the *bottom platen* (4).
13) Place the internal/central long *PEEK baffle* (17) on the *bottom platen* (4).
14) Take the *pressure vessel* (5) with one *external PEEK baffle* (18) slid in its bottom. Place both over the *bottom platen* and place the top *external PEEK baffle* (18) in the *pressure vessel* (5). The *pressure vessel* can be attached on the *bottom platen* using the 6 M8 x 20 socket screws (6). The socket screws have to be tightened gradually and evenly in a star pattern. As highlighted on the picture 8 of Fig. S1, 1 mm thick washers can be used to protect the *pressure vessel* (5) surface when tightening the screws and compressing the O-ring.
15) At this stage, the cell can be prefilled with silicone oil. Given that the internal volume of oil is about 6 mL, this can be a rather long operation if using the high-pressure syringe pumps (volume of the Cetoni syringes is usually 3 mL). Make sure the confining pressure HPLC fluid port of the bottom is closed and fill the *pressure vessel* (5) from the top with a syringe. Wait a few minutes and gently agitate the *pressure vessel* to get rid of the air bubbles that could be stuck at the different interfaces within the cell.
16) Take the *top platen* (7) and fit a *PTFE BS023 O-ring* (B) into the dedicated groove. Slide gently the *top platen* over the *top piston* (19) and put it in contact with the *pressure vessel* (5). The top platen can now be attached using the 6 M8 x 20 screws (6) as on step 14.
17) Push an *EM010x2 (10*2) FKM/FPM/Viton O-ring* (C) over the *top piston* (19) and slide it into the dedicated *top platen* (7) groove. It is followed by the *seal retainer* (20) and the *seal retainer nut* (21). Tighten the *seal retainer nut* (21) to the limit of its travel and loosen fractionally (¼ of a turn) to ensure free axial movement of the *top piston*.
18) Take the *actuator carrier* (8) and fix the *LVDT holder* (9) on its uppermost part so that the LVDT can be held over one of its windows. This operation only must be done once, and the aluminium holder can be left on the actuator carrier for the next experimental runs.
19) Thread the *actuator carrier* (8) onto the *top platen* (7). Make sure both threads have been previously wiped with anti-seize copper grease to avoid any issue to dismantle them at the end of the run.
20) Insert two *EM010x2 O-rings* (C) into the grooves in the *top piston cap* (22). Press this part gently over the *top piston* (19). Once seals are emplaced both over the *piston*, a clear “click”

sound can be heard when the top of the piston encounters the end of the cavity of the *top piston cap* (22). Make sure that the 3.2 mm through hole of this part is aligned with the 3.2 mm bore of the *top piston*.

21) Fix the *LVDT tell-tale* (12) on the top of the *top piston cap* part (22). Make sure it exits the *actuator carrier* (8) on the side where the *LVDT* will be located (9).
22) Insert the top cartridge heater in the 3.2 mm hole in the top piston. Great care must be taken in this operation as the hole is long and cartridge's wires have to be fitted into the drilled hole too in order for the heater to reach the bottom of the bore.
23) Place the *spacer* (11) above the *top piston cap* (22) and *LVDT tell-tale* (12) while making sure power and thermocouple wires exit via the slot in the spacer and are not trapped by the spacer.
24) Screw the *ENERPAC CST 40132 actuator* (10) into the *actuator carrier* (8). Tighten the *actuator* gently against the *spacer* to prevent any vertical movement during the application of the confining pressure. Overtightening of the actuator risks breaking the sample or chipping the sample's edges.
25) Mount on rotary stage with the *M6 breadboard* (1). Connect all the fluid lines via HPLC and Swagelok fittings. Insert the LVDT in the *holder* (12) and compress it against the *tell-tale* (9) (calibration is required prior the run, see supplementary section S5). Do not over-tighten the screws on the LVDT as any compression of the metal case can affect the sensor's operation. Connect the heaters and thermocouples to the power supply and I/O Cetoni modules. Make sure all of these are strain relieved and cable tied against the rig, baseplate etc., to avoid any tension on fluid connectors during rotation, prevent electrical cable abrasion or rupture of a fluid line.
26) The experiment is ready to go. Make sure all the system is electrically earthed and appropriate protection is taken for users and staff.

S4. Temperature gradients within the apparatus and the sample, and their calibration.

The current configuration of *Heitt Mjölnir*, does not allow a thermocouple to be located in or near the sample. Indeed, it is difficult to fit the cartridge heater and a fluid inlet in a single 10 mm OD piston, while maintaining its strength. The fluid paths in the *top* and *bottom pistons* have 90° bends for fluid connections to HPLC tubing and do not allow the thermocouple to be slid in. Similarly, a thermocouple cannot be inserted in the sample through the copper jacket as it would break the seal.

Modified versions of *Heitt Mjölnir* allow the insertion of thermocouples, through the *bottom platen* into the confining pressure space and through to the *top piston* into the sample for the thermal characterisation of the cell. These modifications are not compatible with the design presented herein as they limit the full pressure range of operation of the cell, decrease its reliability and require specific safety procedures. For thermal profiling, a pyrophyllite cylindrical sample was chosen for its low thermal conductivity (typically worse than most experimental samples) and good machinability. The pyrophyllite was fired to 623 K to prevent dehydration and water loss during heating. A 1 mm hole was

drilled in the sample to permit thermocouple access. Once aligned with the top piston's 1 mm hole, a 0.5 mm OD type K thermocouple was inserted and used for the temperature profiling. Once at maximum pressure and temperature conditions, the thermocouple was incrementally withdrawn to enable measurement of a thermal profile.

Various conditions and materials were tested to find the geometries that minimise the thermal gradient observed in the sample. These experiments lead to the choice of the materials and dimensions for the thermal bonding sleeve, baffles, and the piston geometry. The modifications required for the profiling (using longer *graphite sleeve*, heating elements further away from the sample, no *copper jacket*, thermal bounding sleeve and the sample) are a worst-case scenario. The thermal gradient provided in this section is an overestimate and there is a more favourable thermal coupling in the final design.

The temperature distribution inside the sample was homogeneous, the minimum temperature was located in the sample centre and raised slightly toward the sample-piston interface. The temperature variation in the sample led to a measured gradient of about 1.8 K/cm. While increasing the cartridge temperature, the temperature response in the sample centre was linear up to the maximum temperature setpoint. The offset between cartridge temperature set on the controller and the measured sample temperature increases as the temperature is raised (see Fig. S2). This offset reached ≈50 K at 573 K in the sample. This difference is due to imperfect thermal contacts at interfaces between cartridges, pistons, jacket bounding sleeve and the sample. The two main sources of heat loss are the vertical transfers along the pistons in addition to the radial cooling via the *pressure vessel*.

The different tests highlighted that the *PEEK thermal baffling* is very efficient. When the silicone oil temperature reaches about 533-545 K in the central area (Fig. S2 C) and near the hotspots in the top and bottom shoulders (for a cartridge temperature of 573 K), the temperature on the aluminium outer surface never exceeds 373 K. Temperature was monitored on different areas of the *pressure vessel* with an array of 8 type K thermocouples fixed at regular intervals from *top platen*, *pressure vessel's* shoulders and central zone to *bottom platen* (Figs. S2 C and D). We observed the temperature to be homogeneous along the array (less than 10 K difference). The temperature was slightly higher near the *top platen* and bottom shoulder of the *pressure vessel* while it was colder in its central area. The emplacement of the heaters can explain this observation, with heating elements hotspots being located on the same vertical plane of shoulders and platens (Fig. S2 A to D). To ensure the pressure vessel retained its strengths, once at high pressures and temperatures, we found that a fan (computer fan) cooler blowing on the *pressure vessel* (approx. 15 cm distance) is sufficient to keep the external temperature below 353 K, even when the cartridge temperature reached 673 K (temperature limit tests). Additionally, we consider the apparatus to be safer during operation in a well-ventilated, cool area (such as beamline). As a safety indicator, we always use the cell with a thermocouple fixed on the outer surface of the *pressure vessel* to monitor its efficient cooling.

We found the temperature observed inside and outside the *pressure vessel* to be very reproducible, both in the offline and synchrotron runs. We tested the apparatus to find its limits, up to 623 K in the sample for about 673 K in the cartridge. Under these conditions, the cell was found to be stable for a duration of about 6 days. However, degradation of the *PEEK baffle* and associated evolution of an unknown gas at these temperatures confirmed the limit of this apparatus in the configuration described in this paper to be 573 K in the sample (623 K in the cartridge).

S5. Load Calibration and stress estimations for Enerpac CST10132 and 40132 actuators (10 and 40 kN)

Heitt Mjölnir has been designed for experiments with applied hydrostatic or deviatoric loading, hence the use of a large 40 kN ENERPAC CST40132 *actuator* which is able to apply an axial load >500 MPa on the sample. However, some experiments may require a lower range of loads and/or a better precision in its application. For these reasons, we also have designed an adaptor to screw on the M48 thread of the *actuator carrier*, which can replace the 40 kN actuator and allows the use of a 10 kN Enerpac CST 10132 actuator.

The actuator is a single acting hydraulic ram with a spring return. The spring resistance must be overcome to drive the piston downward and apply axial load to the sample. With the effective internal area of the actuator provided from the manufacturers, loads delivered by the applied hydraulic pressure can be calculated. Our experience indicates that actuators can deviate from these estimates in practice, and therefore we have calibrated our actuators against a load cell to ensure the applied load is known. The comparison between calculated and measured load for 40 and 10 kN actuators is given in Fig. S3. Our calibration setup consisted of inserting a load cell in *Heit Mjölnir*, which was fully assembled with a dummy titanium sample (diameter of 9.96±0.01mm and length of 19.97±0.01mm). The load cell was inserted on the *spacer* and was loaded directly by the *actuator*. We used 50 kN and 20 kN button style load cells (series LCM304) from Omega Engineering for calibration of the 40 and 10 kN actuators, respectively.

Our observations show that the load applied is constant at a constant fluid pressure for the permissible pressure range for the actuators (0-35 MPa), even at very low pressures where pressure sensors exhibit noisier values (2% at 1 MPa). The spring compression effect was identified starting from 0.05 to 0.09 MPa (see Fig. S3). In that 0.04-0.1 MPa range, the load evolution is non-linear because of the spring compression within the actuator. From 0.1 MPa to the maximum operating pressure (>35 MPa) the force delivered is a linear function of the actuator fluid pressure. For these reasons, we selected two to three fits, one polynomial fit at low fluid pressure (0-1 MPa) and one to two linear (addition of one fit for 1 to 1.9 MPa range, to better reproduce measured loads of the 40 kN actuator) fits above 1 MPa (fits for each region are provided on Fig. S3). In addition, we observed a small hysteresis of the applied load at similar pressure on the depressurization path with values measured slightly superior to

the values measured at the same pressures on the pressurisation path. We attribute this difference to the accuracy of our Cetoni pump system pressure sensors. For the calibrations, given in Fig. S3, we selected the pressurisation curves as they replicate experimental protocols. While loads delivered by the hydraulic actuator are constant at a given pump pressure, the resulting pressure of the sample is a function of its diameter, stress estimations given in the Fig. S3 are examples for our titanium sample (diameter = 9.96 mm).

S6. Stiffness calibration of the apparatus

Heitt Mjölnir's design is constrained by the X-ray transparency required to perform operando studies and by consequence, the rig is less stiff than equivalent, full-size laboratory rock deformation apparatus. Therefore, it is important to measure and quantify how this device behaves during experiments at high stresses. This is mandatory to interpret the axial variations measurements from the LVDT and subtract deformation of the rig to infer the real strain of the sample.

Offline tests measured the apparent Young's modulus and estimated the stiffness of the cell. The results are provided in Fig. S4. Prior to the experiment itself, we calibrated the LVDT in its position during experiments, (i.e compressed against the tell-tale) and by using feeler gauges to mimic axial displacements. Given the sensitivity of LVDT, we recommend calibrating the apparatus prior to each series of experiments. The calibration of the LVDT shows a linear relation with displacement for ranges compatible (Fig. S4) with sample deformation (around 1 mm, $\varepsilon \approx 5\%$).

For the stiffness measurement, the cell was assembled with a grade 5 cylindrical titanium sample (diameter of 9.96±0.01mm and length of 19.97±0.01mm). The sample was inserted into a silicone jacket sealed with wire loops at the top and bottom of the sample (as on Mjölnir, Butler et al., 2020) because these tests were realised at room temperature. Two stiffness calibration runs were performed, first without confining pressure (no confining fluid in the cell) in a uniaxial compression (left column in Fig. S4) and then with a confining pressure of 20 MPa, corresponding typical triaxial conditions for the apparatus (right column in Fig. S4). The triaxial run was performed to check if the pressurisation of the rig and the loading of its wall, contacts, threads affect the stiffness of the cell. Once confining pressure has been set, the axial load was gradually increased on the titanium sample. The fluid pressure on the actuator was increased at a constant rate of 15 µl/min after a quick increase in the first 0.08 MPa to compress the actuator spring (see calibration curves above). Once at the maximum axial stress, chosen well below the yield strength of the sample (the test is carried in the sample's elastic regime), the load was maintained for a few minutes to stabilise and taken down to room pressure at the same rate. After the experiments the sample length and diameter were checked and did not show any variation greater than 0.01 mm indicating the sample was not permanently deformed.

The LVDT signal can be converted to a strain value using the calibration realised prior the run and the stress applied vertically on the sample is inferred from the load cell calibration described above.

The stress-strain curve highlights the combined behaviour of the machine and the sample during compression and decompression. We can observe a slight deviation on the decompression curve highlighting a small hysteresis (machine not being fully elastic) which can be quantified at low stresses. As most experiments are performed with a compression/loading path, only the loading part of the curve was selected and fitted for apparent stiffness estimation. We observed that the loading path of the cell is characterised by a first part at very low loads (below 1 MPa pressure on actuator, corresponding to <4 MPa axial load), where the apparatus seems to deform significantly (strains up to 0.16%) while the load applied is almost null. The second part is located above 4 MPa axial stress, where the device behaves linearly with the increasing axial load. The low load behaviour observed is due the cell response to the load, loading of contacts between parts, locking the threads and the bolts. The actuator spring loading also corresponds to the 0-1 MPa range, where significant deviation to theoretical predicted delivered force was observed (see Fig. S4). As rock deformation studies require axial stresses >>50 MPa, the non-linear behaviour at very low loads is unlikely to have a significant impact for such studies. Fitting the loading curve above 4 MPa of axial load allow estimating the apparent Young's modulus (E_{app}), defined as:

$$E_{app}(GPa) = \frac{\sigma(GPa)}{\varepsilon_{measured}} \quad (S1)$$

With σ (GPa) corresponding to the sample stress and $\varepsilon_{measured}$ to the total measured axial strain. This equation is valid if the sample and pistons have the same diameter (e.g close to 10.0 mm±tolerance), and stress being equal in the vertical column of the rig. If sample diameter differs from the piston's one, Eq. S1 should be written using applied forces on respective areas instead of stress. The apparent Young's modulus is resulting from contributions of the sample and the apparatus as the measured strain is a combination of elastic responses of the rig and the sample. The machine stiffness (E_{mach}), without sample's contribution, can be calculated from the apparent and sample Young's moduli:

$$E_{mach}(GPa) = \frac{1}{\left(\frac{1}{E_{app}(GPa)} - \frac{1}{E_{sample}(GPa)}\right)} \quad (S2)$$

Using a grade 5 titanium Young's modulus of 114 GPa (Matweb; https://www.matweb.com/search/DataSheet.aspx?MatGUID=10d463eb3d3d4ff48fc57e0ad103743) and apparent Young's modulus of 7.1 GPa for uniaxial and 7.3 GPa for triaxial tests, machine stiffness of 7.6 and 7.8 GPa are inferred, respectively. From these values the axial length variation from an unknown sample can be inferred from LVDT measurements via subtracting the effect of the machine on the signal.

$$\varepsilon_{sample} = \varepsilon_{measured} - \varepsilon_{mach} \quad (S3)$$

$$\varepsilon_{sample} = \varepsilon_{measured} - \frac{\sigma(GPa)}{E_{mach}(GPa)} \quad (S4)$$

The correction of the rig deformation (Eq. S3) is however not fully accurate as indicated by the fact that the theoretical sample strain of the dummy titanium sample cannot be fully recovered (see Figs. S4 F) and G). Indeed, such an equation does not account for the small strain shift due to the rig behaviour at low loads. Hence, for this apparatus, the equation must be adapted to subtract these effects. The shift value is obtained by fitting affine equation (S5) on the stress-stress plot and calculating the value of strain at a stress value equal to 0 MPa. The correction equation is the following, E_{app} (GPa) and *b* (the intercept of the affine equation) being constants:

$$\sigma\ (GPa) = E_{app}(GPa) * \ \varepsilon_{measured} + b\ (S5)$$

$$\varepsilon_{sample} = \varepsilon_{measured} - \frac{\sigma(GPa)}{E_{mach}(GPa)} - \frac{-b}{E_{app}(GPa)}\ (S6)$$

The correction factor has been found to be $\frac{-b}{E_{app}(GPa)}$ = 0.0014 and 0.0018 for uniaxial and triaxial calibrations, respectively. In the former equations, the axial stress (σ) should be interpreted as the total axial stress on the sample if the strain relative to sample length at ambient pressure needs to be obtained. If the strain relative to the sample length after application of confining pressure is wanted, differential stress (σ_1- σ_3) should be used instead.

The results of the calculation of the sample axial strain at a given stress are given in Figs. S4 F) and G). The first observation is that, for a stiff sample, the correction needed is significant due to the greater deformation of the rig. The final calculated strain shows a sigmoid curve centred on the theoretical grade 5 titanium strain curve. This correction curve is not valid for very low stress (<4 MPa due to the correction and fitting steps detailed above). Its non-linear behaviour is relative to the slight nonlinearity of the stress strain curve compared to its fitted curve (Figs. S4 D) and E). This effect is due to the magnitude of the correction and grade 5 titanium being extremely stiff (very small strain). Rock samples will have a Young’s modulus much closer to the device (5-40 MPa) and deviation between theoretical and calculated stresses will be significantly reduced.

These results highlight that the stiffness of the rig is limited compared to offline equivalents, this due to the X-ray transparent *pressure vessel*. Consequently, *Heitt Mjölnir* will not provide the finest resolution regarding bulk sample strain from LVDT measurements. However, this can be complemented with X-ray image analyses of the full sample at different stress steps, which can estimate both local and bulk strain with a resolution of a few micrometres.

Finally, experiments performed at constant stress, such as the gypsum dehydration runs displayed in the deployment section of the manuscript, do not suffer from the low stiffness of the apparatus. All of the axial variations observed during reactions are relative to the “initial” sample length, selected at a given confining and differential stress (see Fig. S6). We also advise using full sample tomography data to estimate absolute strain from room pressure dimensions for better precision.

S7. X-ray flux transmission and absorption modelling

The deployment of X-ray-translucent experimental devices at synchrotron light sources requires a good knowledge of the X-ray beam characteristics, the beamline setup and the rig's component materials to optimise both the incoming synchrotron radiation, the detection system as well as the design of the rig for the best possible imaging performance. This includes either the choice of monochromatic X-rays at the right energies or the proper choice of filters and/or mirrors to tailor the white beam X-ray beam spectrum. Furthermore, the choice of scintillator material and thickness to detect the X-ray beam, as well as the materials used in the rig fabrication are considered. To facilitate the deployment of Heitt Mjölnir to the new imaging beamlines, we provide a python Jupyter notebook as part of the supplementary materials of this article (Flux_calculation_HeittMjolnir.ipynb). It is aimed at simulating the X-ray beam spectrum and the X-ray absorption/transmission characteristics of the rig and sample to predict their effect on the imaging setup. The notebook uses the TOMCAT beamline (SLS) settings used during those experiments as an example for the calculations (see the static version of the notebook: Flux_calculation_HeittMjolnir.html) but can easily be edited to adapt the simulation to another beamline or rig design. Using this tool, it should be possible, on the one hand, to obtain some guidance on how to prepare the beamline in terms of optics and photon delivery/detection, and to estimate, on the other hand, the attainable absorption contrast and achievable measurement speeds prior to the actual experiments.

The flux calculations for the bending magnet source are performed using the xrt (K. Klementiev and R. Chernikov, 2014) python library. Material transmission and absorption are calculated using the XrayDB [https://doi.org/10.5281/zenodo.7574458] library. The calculations are separated into three steps. (i) the calculation of the "raw" bending magnet source spectrum, (ii) the material definitions and their absorption/transmission calculation, and (iii) combining the information from the two previous steps to simulate the effect of the materials on the spectrum and obtaining quantitative flux measurements.

Fig. S5 A) shows the energy-dependent transmission curves for the materials used in the different components of the experiment, including the beamline itself (X-ray windows and air gaps), the filters used to shape the spectrum, the Heitt Mjölnir rig, and the gypsum sample to be investigated. For each component, the contributions of its individual constituent materials are plotted as well as the combined transmission of all materials making up the component.

In Fig. S5 B), the effect of the different components on the beam spectrum is shown. Due to the huge impact on the flux values, linear and logarithmic graphical representations are given. The left-hand panel emphasises the relevant flux range to best observe the effects of the rig and sample on the

beam on a linear flux density scale, while the right panel shows the same curves on a logarithmic scale, providing a bit more context with regards to the full bending magnet and beamline spectra. The green curve labelled “Filters”, representing the flux incident upon the Heitt Mjölnir rig after filtering the beam. The solid green line represents the average flux density within the region of interest, while the dashed green line corresponds to the part of the transmitted flux that is absorbed by the scintillator and hence contributes to the imaging. The shaded area around the green curve delimits the maximum variation of the flux density throughout the vertical field of view due to the energy-dependent flux distribution around the orbital plane of the synchrotron and the beam hardening effects of the absorbing materials. Shown in red is the flux after passing the empty Heitt Mjölnir rig, and the purple curve includes the gypsum sample inside the rig. The calculation highlights that only a small fraction of the beamline flux can be used for the experiment because Heitt Mjölnir acts as a strong filter itself, requiring hardened beams to be reasonably transparent.

It should be noted that the calculations are certainly not exact and do contain several approximations and simplifications. For example, all materials are assumed to be slabs of constant thickness along the beam. For the cylindrical rig itself, this is not true in practice, as the effective material thickness is a function of the distance of the beam passing from the symmetry axis of the rig. As such, the simulations here are valid for the X-ray path through the centre of the rig. Also, for example, the choice of energy range to use in the simulation obviously affects the integrated intensity values. Wherever possible, such approximations or assumptions are mentioned in the notebook to alert a user to the effect of these approximations or assumptions on the calculation results. As such, the numbers reported by the notebook should be treated as approximations and estimates, not absolute values. Nevertheless, they model the actual behaviour in the experiments quite accurately, as a direct validation against measured data in Fig. S5 C) shows.

Some key simulated numbers from the notebook predict a detectable transmission of the rig of ca. 22.1% in its centre and an overall transmission of about 11.5% with the rig and gypsum sample combined. These numbers match very well with the measured transmission of the rig and sample combined of 11.1% (see Fig. S5 C). The total power deposited by the synchrotron into the rig is approximately 20 mW, while the sample receives about 2 mW.

S8. Accuracy, precision: errors estimation on parameters controlled by Heitt Mjölnir

This section provides the determination of the accuracy, precision and the errors associated with the different variables controlled, measured, or derived from Heitt Mjölnir. These estimations rely heavily on the setup chosen, such as the high-pressure pumps, and are provided as guideline estimates. This guide helps the design of the experiments, evaluating the possibilities and anticipating the limitations of the characterisations performed with this device.

All errors (σ) given in the section are reported to be one standard deviation (confidence interval of 68.2%). In the estimation and error propagation given below the covariance terms between parameters have been neglected. A sum up of errors on parameters inferred from calibration runs and an example experiment (Figs. 4 and S6) are provided in the table S3.

S8.1. Pressures

The pressure control for confining, pore fluid pressure and axial differential stress in Heitt Mjölnir is hydraulic and relies on the precision of the high-pressure syringes and pumps directly connected to the device.

S8.1.1. Confining, pore fluid and actuator fluid pressures

For Cetoni high-pressure modules, the pressure is monitored via an alumina sensor (0.5-4.5V output). The accuracy error provided by the supplier for such a sensor is 1.0% f.s (full scale), with 0.6 MPa at 60 MPa. However, we found that these values are very conservative and calibration of our pumps against a Wika master gauge demonstrated a much better accuracy. It confirms good linearity of the sensor from 5 MPa onwards and excellent correlation at high pressures (measured relation from 0-30 MPa: Master gauge pressure (MPa) = 1.0084 x Pump gauge (MPa) -1.66 with $R^2 = 1$). The offset of the pressure sensor was found to be 0.1 MPa at 5 MPa and decreasing to 0.04 MPa at 30 MPa.

In addition, our tests showed reliability, repeatability and more importantly a linear behaviour on the whole pressure range. These accuracy considerations are estimates of the sensor bias and can be addressed by such calibration. For variation during a single experiment, measurements being relative, the bias is not considered. We estimated the random errors on the logged data by using the standard deviation during test experiments maintained at various pressures for a long duration. The random error observed was nearly independent of the set pressure with a standard deviation of less than ±0.02 MPa.

S8.1.2. Differential stress

To retrieve the differential stress on the sample, the actuators have been calibrated with the procedure described in the supplementary section S5 and Fig. S3. We first calibrate the actuator against a load cell. The error given for the load cell (50 kN) used for this calibration is very limited with ±0.076 V (e.g 0.7%) on the output signal (0-22 V). The calibration was performed with a high precision Keithley voltmeter, having an offset of less than 0.03 mV, and errors on values read well below 1%. For simplicity (errors being well below 1%), we assume the supplier calibration of the load cell to be absolute and only consider the load at hydraulic pressures above 1 MPa on the actuator (as behaviour is nonlinear below this pressure, see supplementary text S5 and Fig. S3 for discussion). The calibration curve of the vertical force applied by the actuator in function of the hydraulic pressure applied is given

in Fig. S5. The errors on fitting parameters were found similar when using the inverse of variance as weights in the linear regression.

$$F_{ram}\ (kN) = \beta * P_{ram}(MPa)/10\ (S7)$$

$$\sigma_{F_{ram}} = \sqrt{\left(\frac{\partial F_{ram}}{\partial P_{ram}}\right)^2 \sigma_{P_{ram}}{}^2 + \left(\frac{\partial F_{ram}}{\partial \alpha}\right)^2 \sigma_\alpha{}^2 + \left(\frac{\partial F_{ram}}{\partial \beta}\right)^2 \sigma_\beta{}^2}$$

$$= \sqrt{\beta^2 \sigma_{P_{ram}}{}^2 + \sigma_\alpha{}^2 + P_{ram}{}^2 \sigma_\beta{}^2}\ (S8)$$

The fitted slope was 0.1158 ±0.0006 (β±σβ) and intercept -0.929 ±0.093 (α±σα). The maximum error on loads (σ_{Fram}) inferred from the calibration is ±0.09 kN at 31.5 kN (0.29%), showing the quality of the regression and the overall good precision of the system.

The axial stress on the sample is estimated from the load applied from actuator and the sample surface.

$$P_{axial}(Pa) = \frac{F_{ram}(N)}{S(m^2)}\ (S9)$$

$$\sigma_{P_{axial}} = \sqrt{\left(\frac{\partial P_{axial}}{\partial F_{ram}}\right)^2 \sigma_{F_{ram}}{}^2 + \left(\frac{\partial P_{axial}}{\partial S}\right)^2 \sigma_S{}^2}$$

$$= \sqrt{\left(\frac{1}{S}\right)^2 \sigma_{F_{ram}}{}^2 + \left(\frac{-F_{ram}}{S^2}\right)^2 \sigma_S{}^2}\ (S10)$$

Samples diameters are measured with digital callipers prior to the experiments and the P-T range accessible with the rig does not allow a significant elastic compression and thermal expansion. The error on the sample diameter (≃10 mm) is ±0.02 mm and the surface is (7.85 ±0.03) x 10^5 m^2 (0.4% error). The propagated errors on the axial stress yield to ±2 MPa at 402 MPa (≃0.5%). The error can be derived for the differential stress similarly:

$$\Delta S = P_{axial} - P_c\ (S11)$$

$$\sigma_{\Delta S} = \sqrt{\sigma_{P_c}{}^2 + \sigma_{P_{axial}}{}^2}\ (S12)$$

S8.2. Stiffness and sample length correction

Stiffness is obtained from the stress/strain curve given in the supplementary section S6 and Fig. S4. To monitor the piston axial strain, a LVDT is used and calibrated as described in the supplementary section S5 and Fig. S4. The LVDT is a high precision tool, but its implementation on the triaxial device can be challenging in electromagnetically noisy environments such as beamlines. The error on the LVDT signal is almost negligible (0.001V onto a signal up to 10 V). The output signal is connected to our Cetoni Nemesys pumps and a Beckhoff 0-10 V 3162 analog input with an error of 0.05% F.S. Most

of the uncertainty is from the calibration steps where feeler gauges are inserted between the LVDT pin and its *tell-tale* to simulate axial displacement. The error on the thickness of the gauge is estimated from the thickness of the gauge T (in micrometre), with: $\sigma_T = 3 + t/80$, an error of 15.5 micrometres on one millimetre gauge. The calibration of the LVDT signal with the axial displacement (*ad*) is linear and fitted with:

$$LVDT\ signal\ (V) = \alpha * ad + \beta\ (S13)$$

$$\frac{LVDT\ signal\ (V) - \beta}{\alpha} = ad\ (S14)$$

$$\sigma_{ad} = \sqrt{\left(\frac{\partial ad}{\partial LVDT}\right)^2 \sigma_{LVDT}{}^2 + \left(\frac{\partial ad}{\partial \alpha}\right)^2 \sigma_\alpha{}^2 + \left(\frac{\partial ad}{\partial \beta}\right)^2 \sigma_\beta{}^2}\ (S15)$$

We computed the error on the slope to be (on the example given in this paper, the calibration is slightly different at each session) 2.409 ±0.02 and intercept 0.0060 ±0.0009. Using Eqs. S14 and S15, errors on the displacement inferred from LVDT signals are usually about 0.5% (table S3). Our experience demonstrated that LVDT calibration varies slightly in terms of slope and regression, but each calibration is always linear ($R^2 > 0.99$). The smallest detectable strain with our system was determined to be in the order of 30 µm, similar to our smaller calibration gauge.

From LVDT measurements, the signal relative to the measurement at t = 0 is calculated. Its error is twice the read error by the Beckhoff reader (e.g 0.1%). The displacement in mm is then retrieved using the calibration step described above and errors propagated similarly. The axial strain is calculated using the axial displacement and the sample height (h, error of 0.02 mm with digital calliper, which can be minimised if using µCT data).

$$LVDT_{relative} = LVDT_{T=t} - LVDT_{t=0}\ (S16)$$

$$\sigma_{LVDT_{relative}} = 2\sigma_{LVDT}\ \ (S17)$$

$$LVDT_{mm} = \frac{LVDT_{T=t} - \beta}{\alpha} - \frac{LVDT_{T=0} - \beta}{\alpha} = \frac{LVDT_{relative}}{\alpha}\ (S18)$$

$$\sigma_{LVDT_{mm}} = \sqrt{\left(\frac{\partial LVDT_{mm}}{\partial LVDT_{relative}}\right)^2 \sigma_{LVDT_{relative}}{}^2 + \left(\frac{\partial LVDT_{mm}}{\partial \alpha}\right)^2 \sigma_a{}^2}$$
$$= \sqrt{\left(\frac{1}{\alpha}\right)^2 \sigma_{LVDT_{relative}}{}^2 + \left(\frac{-LVDT_{relative}}{\alpha^2}\right)^2 \sigma_a{}^2}\ (S19)$$

The total measured axial strain ($\varepsilon_{measured}$) can be inferred from the LVDT signal:

$$\varepsilon_{measured} = \frac{LVDT_{mm}}{sample\ height\ (h)}\ (S20)$$

$$\sigma_{\varepsilon_{measured}} = \sqrt{\left(\frac{\partial \varepsilon_{measured}}{\partial LVDT_{mm}}\right)^2 \sigma_{LVDT_{mm}}{}^2 + \left(\frac{\partial \varepsilon_{measured}}{\partial h}\right)^2 \sigma_h{}^2}$$
$$= \sqrt{\left(\frac{1}{h}\right)^2 \sigma_{LVDT_{mm}}{}^2 + \left(\frac{-LVDT_{mm}}{h^2}\right)^2 \sigma_h{}^2}\ (S21)$$

During our stiffness calibration, the maximal strain measured was 0.0418 ±0.0002 (0.5%). The apparent Young's modulus is fitted from the linear relation displayed in the strain/stress curves:

$$\varepsilon_{measured} = P_{ram} * \beta + \alpha\ with\ \beta = E_{app}\ (S22), see\ also\ S1$$

Due to the good quality of the fit and quasi-perfect linearity of stress/strain curve above 1 MPa on the actuator, the error on apparent Young's modulus is minimal: 7.1343 ±0.0071 GPa (0.1%). Machine stiffness and errors are derived from Eq. S2 assuming 5 GPa of error for grade 5 titanium Young's modulus.

$$\sigma_{k_{mach}} = \sqrt{\left(\frac{\partial k_{mach}}{\partial E_{app}}\right)^2 \sigma_{E_{app}}{}^2 + \left(\frac{\partial k_{mach}}{\partial E_{sample}}\right)^2 \sigma_{E_{sample}}{}^2}$$
$$= \sqrt{\left(\frac{E_{sample}}{\left(E_{app} - E_{sample}\right)^2}\right)^2 \sigma_{E_{app}}{}^2 + \left(\frac{E_{app}}{\left(E_{app} - E_{sample}\right)^2}\right)^2 \sigma_{E_{sample}}{}^2}\ (S23)$$

Errors on machine stiffness are an order of magnitude higher with 7.611 ±0.024 GPa, e.g 0.3%. Sample strains inferred with the device will have to be corrected using the Eq. S6 provided above.

$$\sigma_{\varepsilon_{sample}} = \sqrt{\begin{array}{c}\left(\frac{\partial \varepsilon_{sample}}{\partial \varepsilon_{measured}}\right)^2 \sigma_{\varepsilon_{measured}}{}^2 + \left(\frac{\partial \varepsilon_{sample}}{\partial P_{axial}}\right)^2 \sigma_{P_{axial}}{}^2 + \left(\frac{\partial \varepsilon_{sample}}{\partial E_{app}}\right)^2 \sigma_{E_{app}}{}^2 \\ + \left(\frac{\partial \varepsilon_{sample}}{\partial k_{mach}}\right)^2 \sigma_{k_{mach}}{}^2 + \left(\frac{\partial \varepsilon_{sample}}{\partial \alpha}\right)^2 \sigma_\alpha{}^2 + \left(\frac{\partial \varepsilon_{sample}}{\partial E_{app}}\right)^2 \sigma_{E_{app}}{}^2\end{array}}\ (S24)$$

Error propagation using the Eq. S24 yields a retrieved sample of strain of 0.0039 ± 0.00028 (0.7%) for our example gypsum dehydration reaction presented in Figs. 4 and S6. For this run, the rig accounted for about 15% of the total observed strain (table S3.2.), highlighting the importance of the calibration steps. For very stiff samples, the correction expected would be larger because the Young's modulus of the sample and the rig are similar. For rock samples, we expect this error to be a few percents.

S8.3. Temperature

Temperature control, measurements and calibration have been performed with type K thermocouples. They were built into our cartridge heaters, fixed inside the pressure vessel and the sample, and fixed on the pressure vessel for calibrations given in the supplementary material section S4. Type K thermocouples have intrinsic errors of 2.2 K. The reproducibility of the temperature measurements, both in the cartridge heaters and the sensors outside and inside the cell, was very good with less than 2.5 K difference observed for repeated runs at similar target conditions. However, the errors on the thermocouple value may not completely represent the reality of the uncertainty in the temperature repartition in the device. Indeed, the quality of the contacts between the thermocouple and parts is difficult to ensure once in the device, this effect cannot be quantified at this stage.

We propagated the errors on the sample temperature calibration using them as weights (inverse of variance) during the linear fitting. Slope value was estimated to be 0.8794±0.0074 and intercept 3.6±1.0. The error was propagated using the following equation:

$$T_S = \alpha T_C + \beta \ (S25)$$

$$\sigma_{T_S} = \sqrt{\left(\frac{\partial T_S}{\partial T_C}\right)^2 {\sigma_{T_C}}^2 + \left(\frac{\partial T_S}{\partial \alpha}\right)^2 {\sigma_\alpha}^2 + \left(\frac{\partial T_S}{\partial \beta}\right)^2 {\sigma_\beta}^2} \ (S26)$$

The computed error was between 2.8 to 4 K from 323 to 573 K in the sample (6.1 to 1.57% of the value) A simple Standard Error of the Regression (SER) would only provide an error of 1.5 K on the estimated sample temperatures.

For thermal gradients estimation, the temperature was profiled (see supplementary section S4 and Fig. S2) via pulling the thermocouple in the sample and is obtained taking the temperature variation over the distance, which is about half the sample height.

$$TG = \frac{T_{centre} - T_{edge}}{D_{centre} - D_{edge}} = \frac{\Delta_T}{\Delta_D} (S27)$$

$$\sigma_{TG} = \sqrt{\left(\frac{\partial TG}{\partial \Delta_T}\right)^2 {\sigma_{\Delta_T}}^2 + \left(\frac{\partial TG}{\partial \Delta_D}\right)^2 {\sigma_{\Delta_D}}^2} = \sqrt{\left(\frac{1}{\Delta_D}\right)^2 {\sigma_{\Delta_T}}^2 + \left(-\frac{\Delta_T}{{\Delta_D}^2}\right)^2 {\sigma_{\Delta_D}}^2} (S28)$$

The temperature of the centre and edge are having an uncertainty of 0.5 K (random uncertainty on the read value) and the distance between intervals is measured with an uncertainty of 0.5 mm. The thermal gradient measured is 0.18 K/mm with an uncertainty of ±0.053 K/mm (29.5%).

Table S3 Example of parameters and their errors determined during calibration and experiment.

Parameters determined during calibration run a) stiffness test with titanium sample and b) temperature profiling and calibration. Values are given for the maximum pressure, stress, and temperature.

Parameter	unit	symbol	example value	error	relative error (%)
Confining pressure	MPa	P_c	20	0.02	0.1
Fluid pressure on actuator	MPa	P_{ram}	28	0.02	0.07
Actuator force	kN	F_{ram}	31.5	0.09	0.29
Sample axial stress	MPa	P_{axial}	402	2	0.5
Differential stress	MPa	ΔS	382	2	0.5
LVDT signal	V	LVDT	2.15	0.001	0.05
Axial displacement calibration	mm	ad	0.889	0.0042	0.47
Total (Ti sample) axial strain		$\varepsilon_{measured}$	0.0418	$2.0*10^{-4}$	0.5
Apparent Young's modulus	GPa	E_{app}	7.134	0.0071	0.1
Machine stiffness	GPa	K_{mach}	7.611	0.024	0.3
Sample temperature	K	T_S	573	4	0.7
Thermal gradient in sample	K/mm	TG	0.18	0.053	29.5

Parameters and errors propagated for gypsum dehydration run into bassanite and anhydrite (Figs. 4 and S6). Values are given for the maximum pressure, stress, and temperature.

Parameter	unit	symbol	example value	error	relative error (%)
Confining pressure	MPa	P_c	20	0.02	0.1
Pore fluid pressure	MPa	P_f	5	0.02	0.4
Fluid pressure on actuator	MPa	P_{ram}	3.4	0.02	0.7
Actuator force	kN	F_{ram}	3.0	0.1	3.3
Sample axial stress	MPa	P_{axial}	40.6	1.3	3.2
Differential stress	MPa	ΔS	20.6	1.3	6.45
LVDT signal	V	LVDT	2.11	0.001	0.05
Axial displacement	mm	ad	3.17	0.015	0.5
total axial strain		$\varepsilon_{measured}$	0.045	$2.2*10^{-4}$	0.5

sample strain (after correction)		ss	0.039	$2.8*10^{-4}$	0.7
Sample temperature	K	T_S	554.5	3.2	0.6

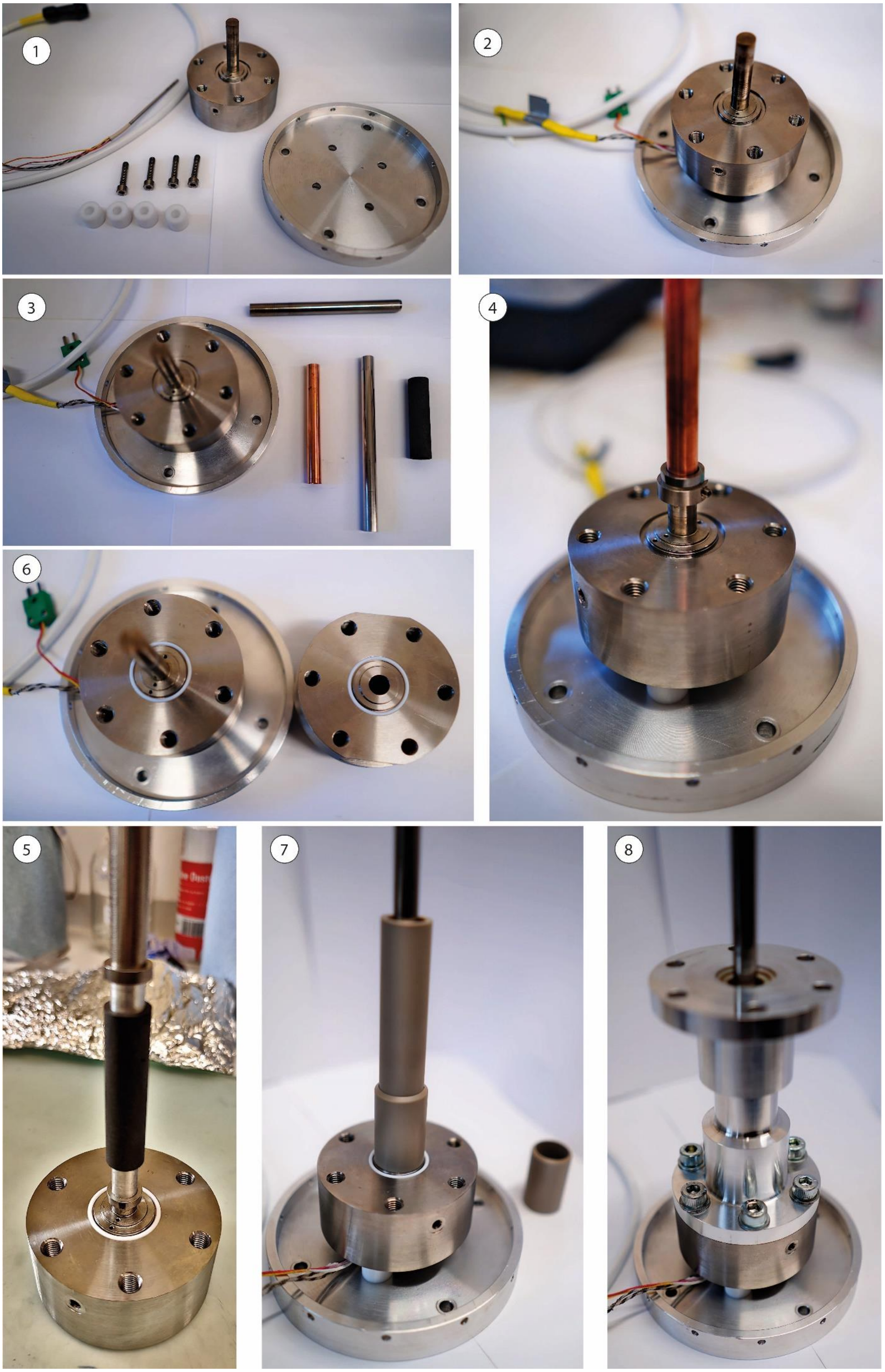
1
2
3
4
6
5
7
8

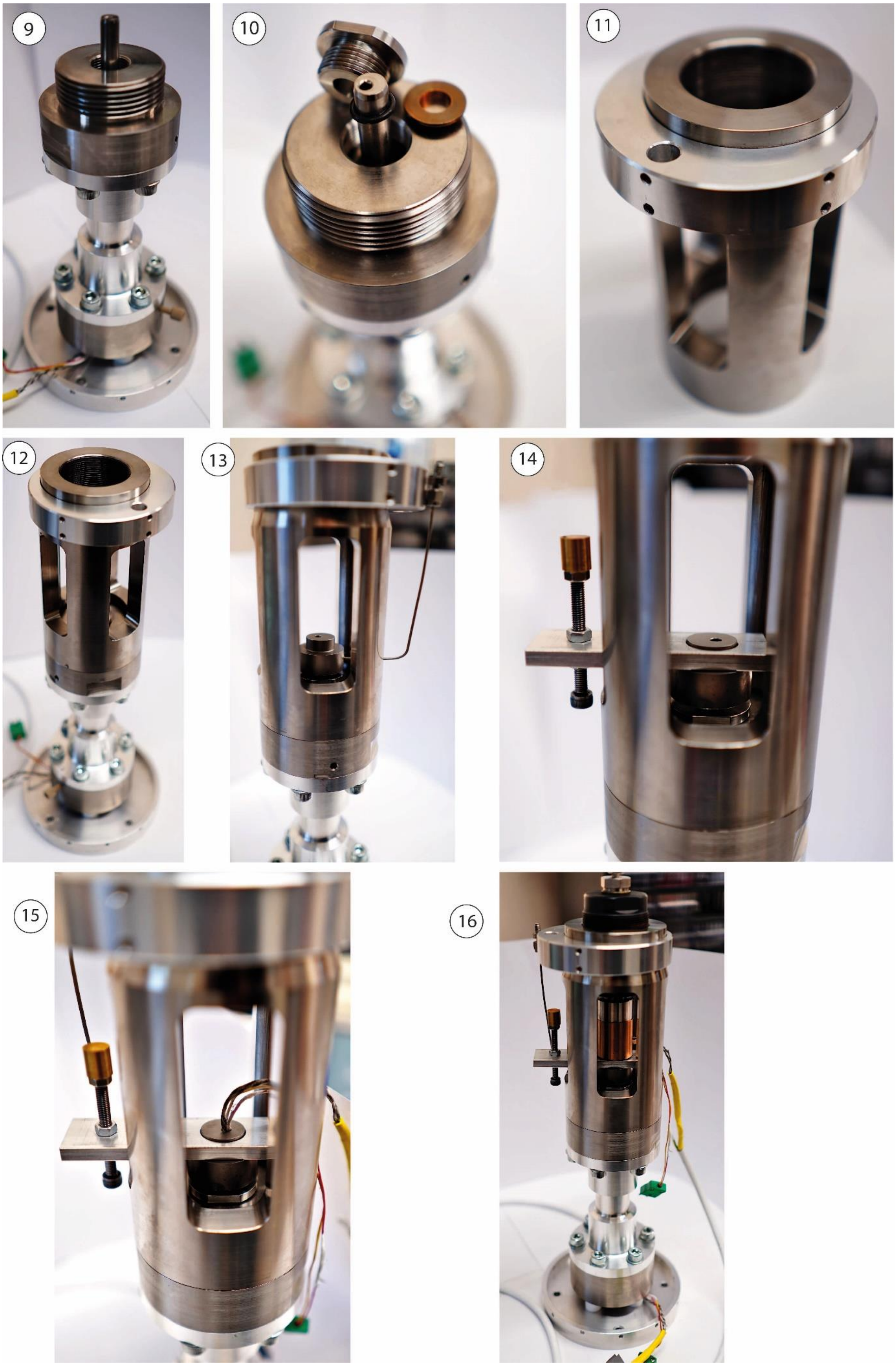
9
10
11
12
13
14
15
16

Figure S1 **Assembly sequence.** Assembly sequence for *Heitt Mjölnir*. Each step is explained in detail in supporting information S2. Photographs (X) details the following assembling steps Y) of text S2: (1)→1); (2)→2) and 3); (3)→4); (4)→5), 6), 7), and 8); (5)→9), 10), and 11); (6)→12); (7)→13); (8)→14) and 15); in first part of the Figure. (9)→16); (10)17); (11)18); (12)→19); (13)→20); (14)→21); (15)→22); (16)→23), and 24) for the second part of the figure.

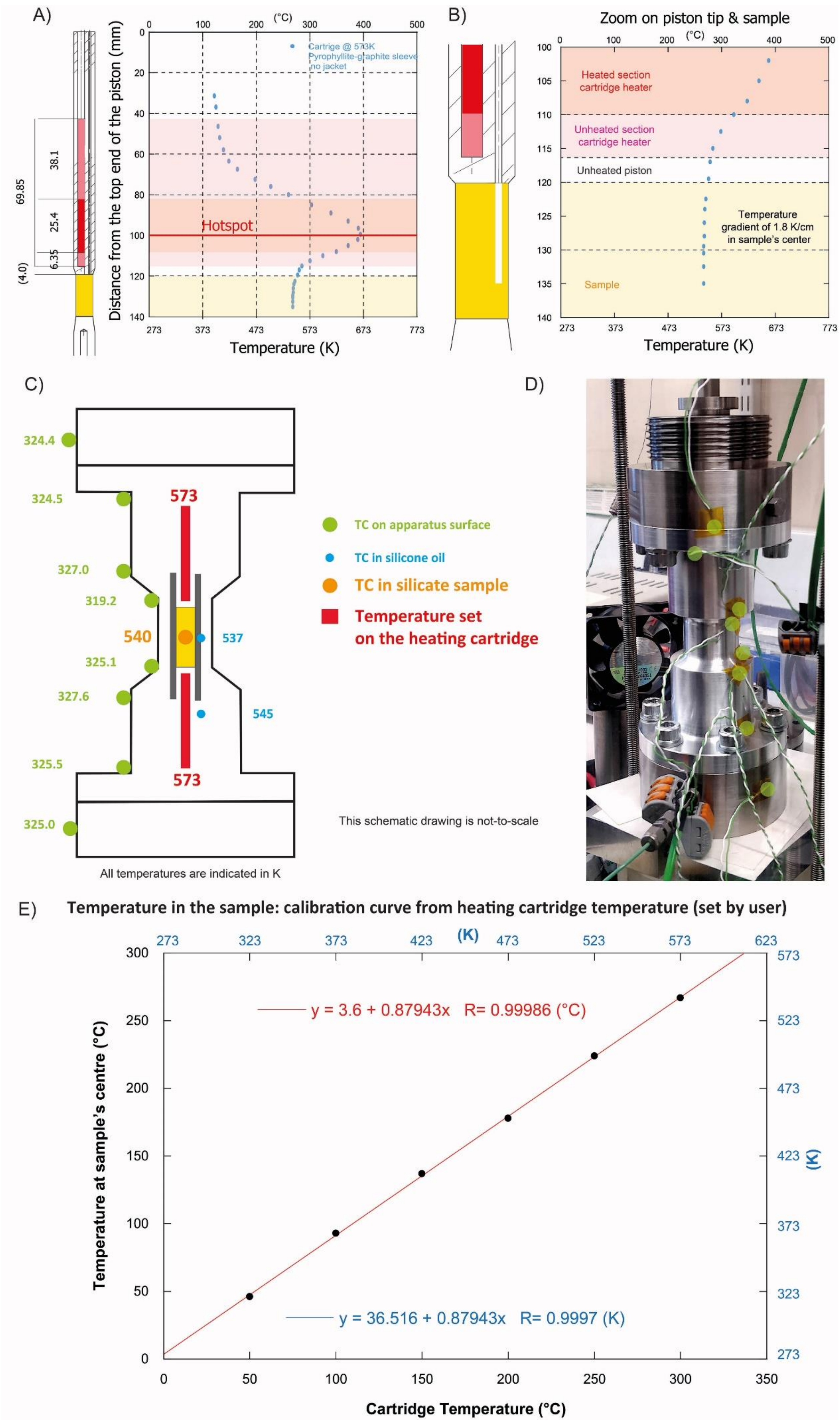
A)
69.85
38.1
25.4
6.35
(4.0)
(°C)
Cartrige @ 573K
Pyrophyllite-graphite sleeve
no jacket
Hotspot
Distance from the top end of the piston (mm)
Temperature (K)
B)
Zoom on piston tip & sample
Heated section
cartridge heater
Unheated section
cartridge heater
Unheated piston
Temperature
gradient of 1.8 K/cm
in sample's center
Sample
Temperature (K)
C)
324.4
324.5
327.0
319.2
540
537
325.1
327.6
545
325.5
325.0
573
573
TC on apparatus surface
TC in silicone oil
TC in silicate sample
Temperature set
on the heating cartridge
This schematic drawing is not-to-scale
All temperatures are indicated in K
D)
E)
Temperature in the sample: calibration curve from heating cartridge temperature (set by user)
(K)
y = 3.6 + 0.87943x R= 0.99986 (°C)
y = 36.516 + 0.87943x R= 0.9997 (K)
Temperature at sample's centre (°C)
Cartridge Temperature (°C)

Figure S2 Heat distribution and temperature calibration in *Heitt Mjölnir*. The temperature profile in the sample and top piston are given in Figs. A) and B). The sample is represented in yellow, the heated part of the cartridge heater in red, the unheated section (outside resistance coils) of the heater in pink and titanium piston in white. The temperature profile displayed was made at a cartridge temperature setpoint of 573 K, the sample was pyrophyllite and a *graphite sleeve* was used as a thermal bond. Figs. C) and D) are showing temperature measurements inside the pressure vessel, with temperatures in the sample centre (yellow), confining silicone oil (blue) and *pressure vessel* and *platens* surfaces (green) at the same cartridge temperature setpoint of 573 K. The measurements on the pressure *vessel's* surface were taken with a fan blowing on its opposite side, as visible in Fig. D). All the measurements (in C and D) are given for a temperature setpoint of 573 K for the cartridge heater (red). Panel E) is the temperature calibration curve to determine the sample temperature from the cartridge temperature (set by the user) in K (blue) or °C (red).

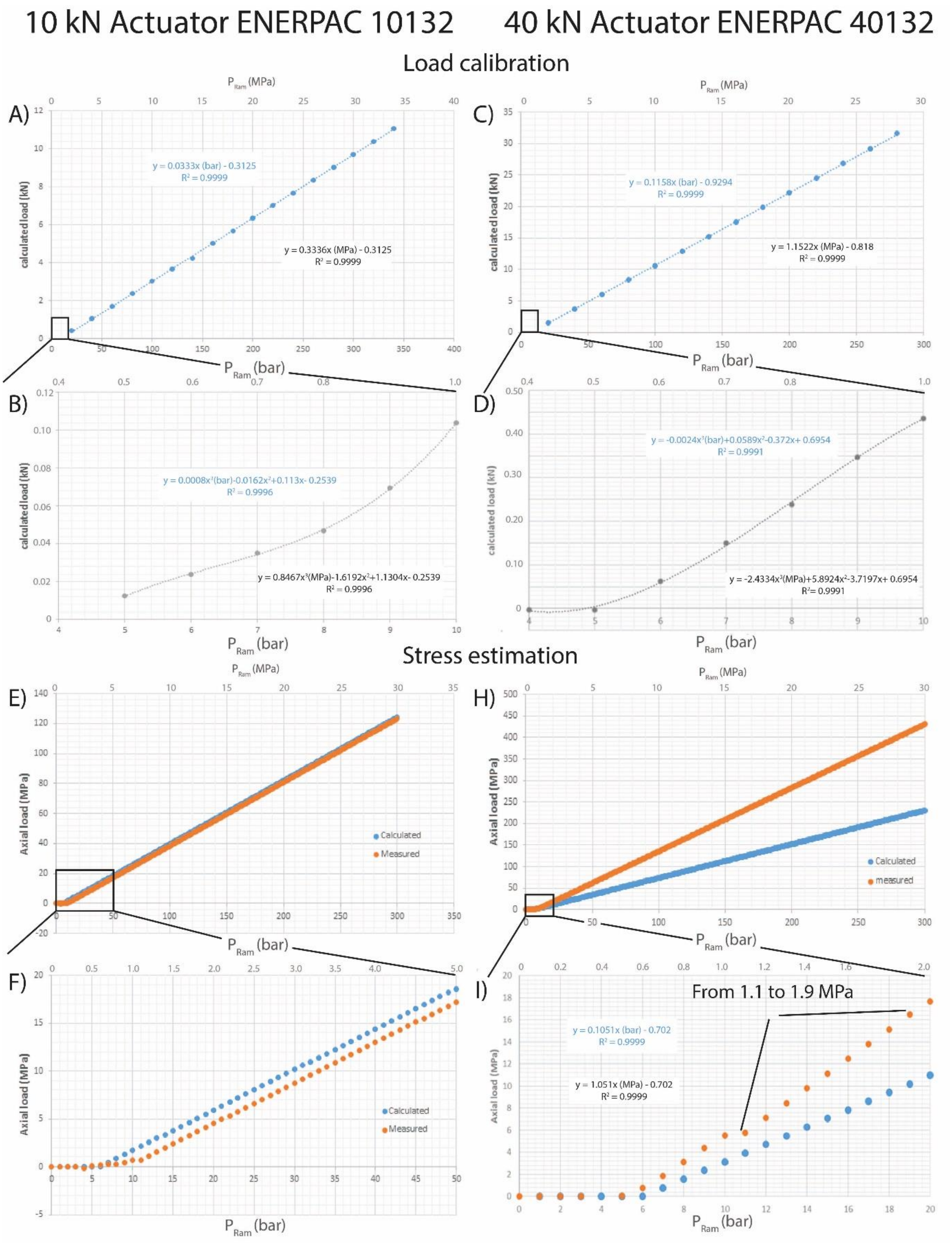

Figure S3 **Load calibration and stress estimations for Enerpac CST10132 and 40132 actuators (10 and 40 kN).** Panels A), B), E), and F) are plotted for 10 kN actuator and C), D), G), and H) for 40 kN actuator, respectively. In the two first rows, the calibration of the actuators is given. The load calculated from our load cell measurements is plotted as a function of the pressure applied on the

actuator. For both actuators, this relation is linear over most of the pressure range (>1 MPa) (A and C) while it deviates from linearity on the first 1 MPa (B and D) due to the compression of the spring (see text S3). Both linear and polynomial fits for low and high loads are given on their respective figures (blue fits are for P_{ram} values in bar and black ones are for values in MPa). For our 40 kN actuator, we found measured data to be better represented via using an additional linear fit between 1 and 1.9 MPa. The fitted parameters (P_{ram} to calculated load) for this range are displayed on panel I). From these measured calibration curves (labelled "measured", in orange), the stress applied axially on the 10 mm diameter sample can be estimated. It is compared with the theoretical stress that can be calculated using the cylinder effective area advance provided from the supplier in blue (see section S3).

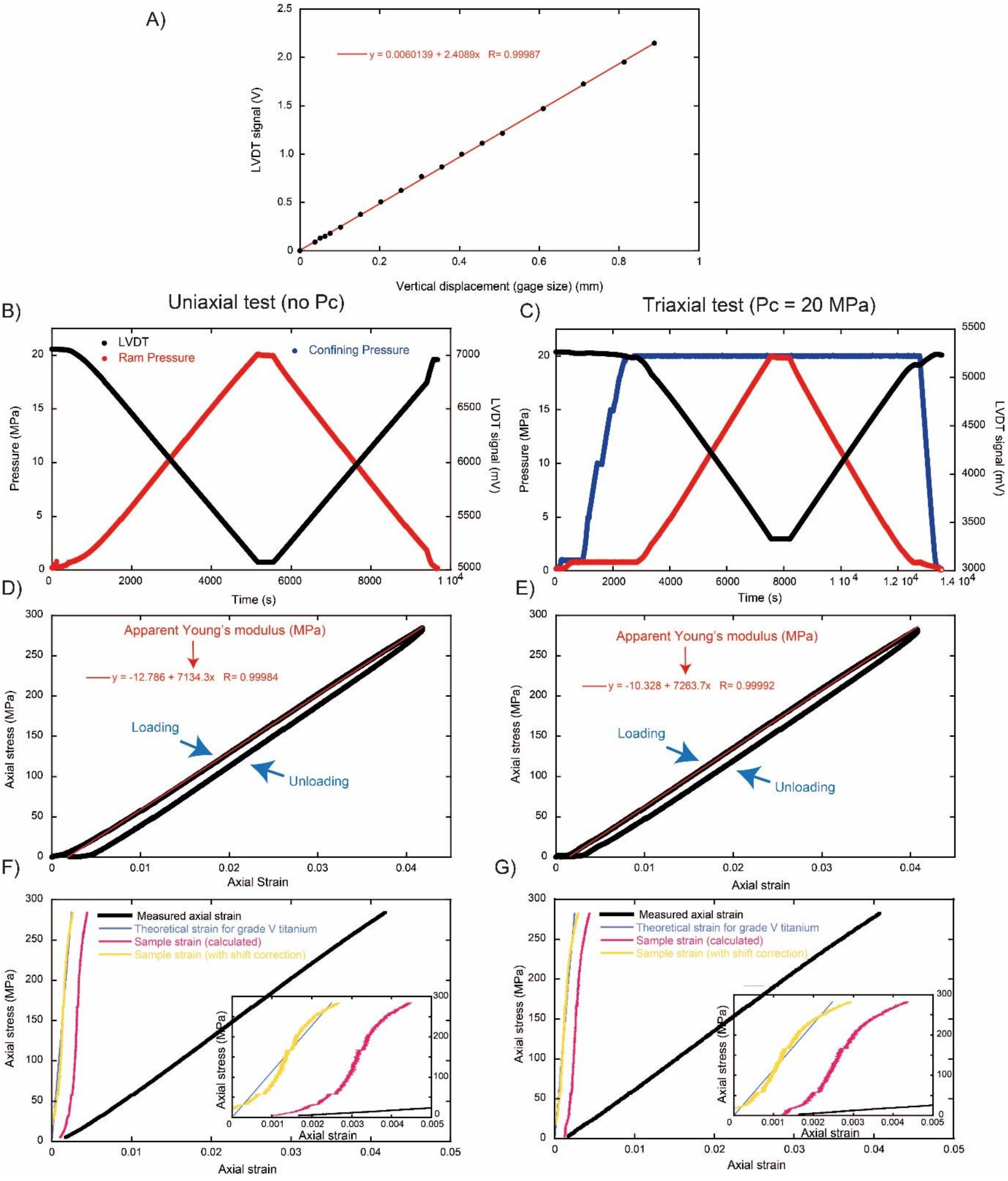

Figure S4 Stiffness calibration tests and sample axial strain estimations. Stiffness calibration steps and results from A) LVDT calibration, to uniaxial B) and triaxial C) experiments on a grade 5 titanium sample, resulting stress-strain curves and apparent Young's modulus fitting D) and E). The sample axial strain calculation from apparent strain and machine stiffness is given in F) and G). The right side of the figure is corresponding to the uniaxial test (P_c = 0.01 MPa / 1 atmosphere) B), D), F) while the right side is for the triaxial test with a confining pressure of P_c = 20 MPa, panels C), E), and G). In Fig. B) and C) Ram (actuator), pressure and confining pressure are presented in red and blue,

respectively (left axis). The LVDT signal is represented in black (right axis). In panels D) and E) the fit on the loading branch of the stress strain curve is given in red. In the last raw F) and G) measured/apparent strain is represented in thick black line, sample theoretical axial strain in blue, calculated sample strain with machine stiffness in pink and calculated sample strain taking in account the strain shifts a lower load in yellow. The interpretation of the loading curves and sample axial strain calculation are detailed in supplementary section S6.

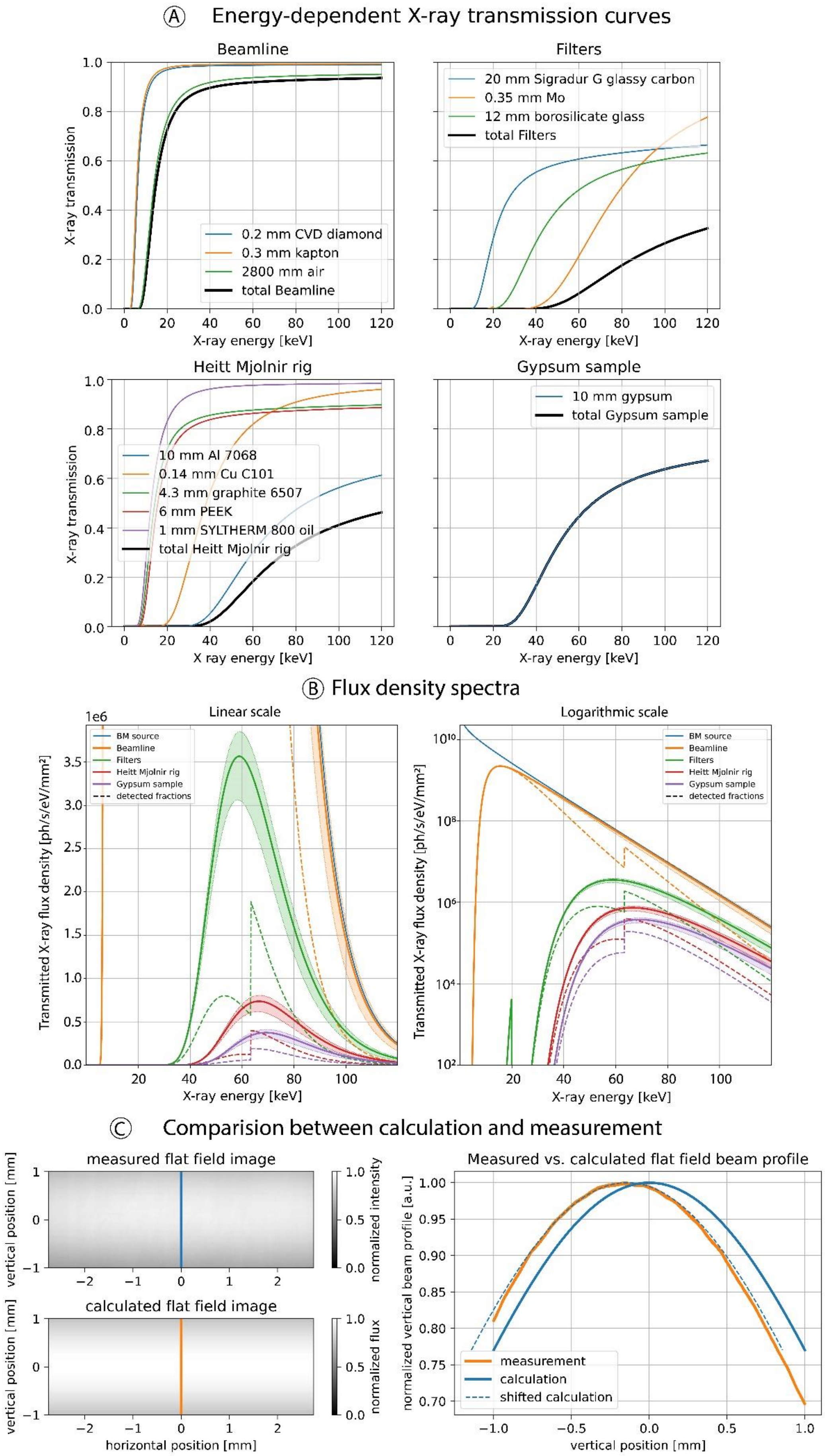
Ⓐ Energy-dependent X-ray transmission curves
Beamline
Filters
X-ray transmission
X-ray energy [keV]
0.2 mm CVD diamond
0.3 mm kapton
2800 mm air
total Beamline
20 mm Sigradur G glassy carbon
0.35 mm Mo
12 mm borosilicate glass
total Filters
Heitt Mjolnir rig
Gypsum sample
10 mm Al 7068
0.14 mm Cu C101
4.3 mm graphite 6507
6 mm PEEK
1 mm SYLTHERM 800 oil
total Heitt Mjolnir rig
10 mm gypsum
total Gypsum sample
X ray energy [keV]
Ⓑ Flux density spectra
Linear scale
Logarithmic scale
1e6
Transmitted X-ray flux density [ph/s/eV/mm²]
BM source
Beamline
Filters
Heitt Mjolnir rig
Gypsum sample
detected fractions
X-ray energy [keV]
Ⓒ Comparision between calculation and measurement
measured flat field image
calculated flat field image
vertical position [mm]
horizontal position [mm]
normalized intensity
normalized flux
Measured vs. calculated flat field beam profile
normalized vertical beam profile [a.u.]
vertical position [mm]
measurement
calculation
shifted calculation

Figure S5 X-ray beam spectrum, materials transmission calculations and their validation. X-ray flux calculations for the experimental setup at the TOMCAT beamline. A) Energy-dependent material transmission curves for the different constituent materials of the experimental setup components. Each panel shows the transmissions of all individual materials contained in a component (coloured lines) as well as the combined overall transmission of that component (thick black line). B) The resulting X-ray flux density spectra after attenuation by each of the beamline components. The thick solid lines correspond to the average flux density over the full field of view. The shaded area around the average indicates the vertical variability of the flux density throughout the field of view. Only the part of the spectrum, which is absorbed by the scintillator crystal, indicated by the dashed lines, can be imaged. The left panel shows the situation on a linear flux density scale, emphasising the filtered spectrum (green) as it gets altered by the presence of the rig (red) and sample (purple). The right panel shows these curves on a logarithmic scale in the context of the full source spectrum and the unfiltered flux spectrum delivered by the beamline. C) Validation of the simulated beam profile after the filters with measured flat field images during the experiments. The average dark field-corrected measured flat field image is shown in the upper left panel, the corresponding calculated detected beam profile in the lower left panel. Vertical line profiles through the images, indicated by the orange and blue lines at the centre of the images are plotted in the right panel. During the measurement, the detector was not perfectly centred on the orbital plane of the synchrotron, resulting in a slight shift of the peak intensity towards the upper edge of the detector. To facilitate the quantitative comparison between measurement and simulation, the simulated curve was manually shifted to match the centre of the measured curve (dashed blue line). The figures were created with the python Jupyter notebook (see supplementary section S7)

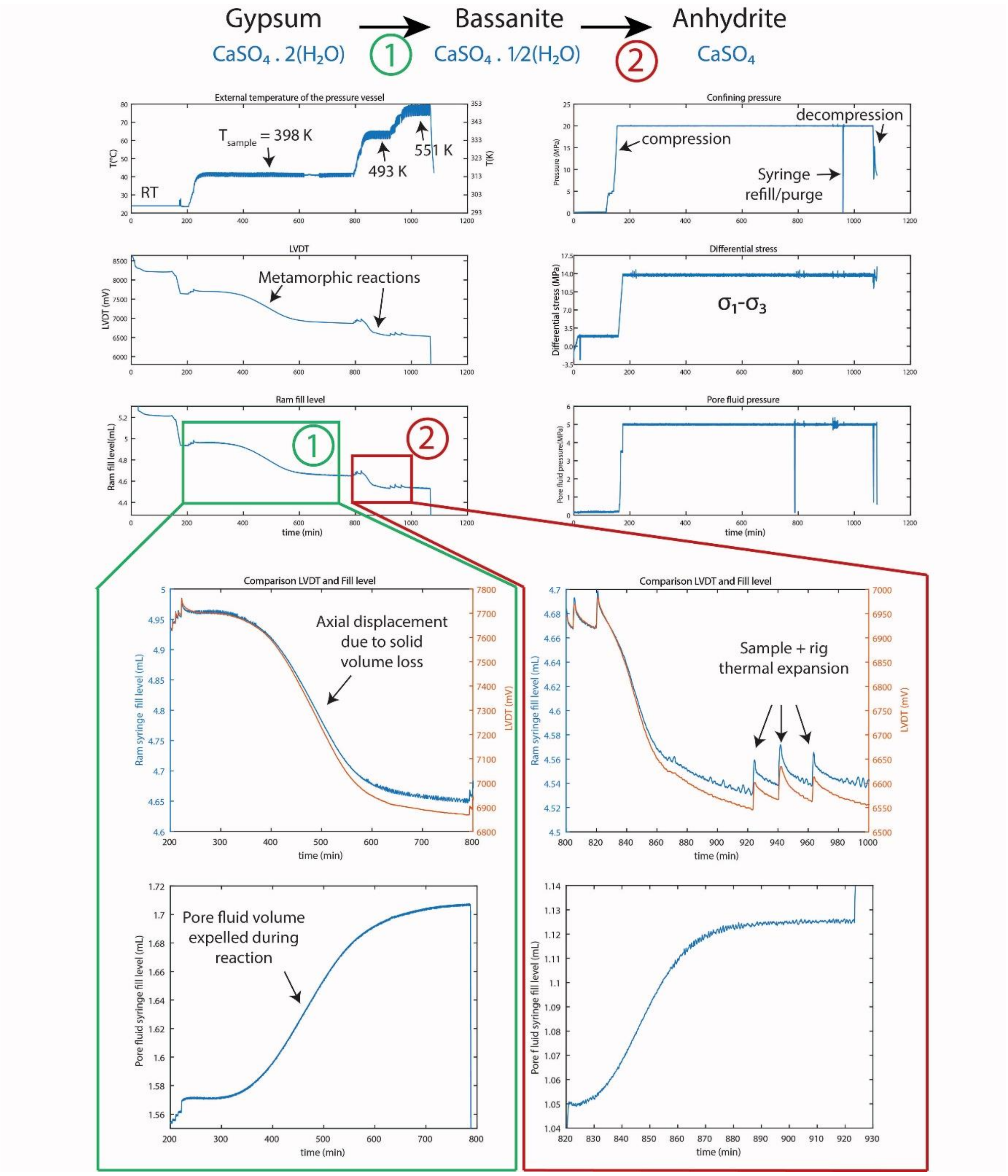

Figure S6 Example of parameters logged during Gypsum dehydration experiment with CETONI high pressure syringed and I/O system. Example of data logged by Cetonix Qmix software during the dehydration experiment of gypsum into basanite and anhydrite at a constant confining pressure of 20 MPa, differential stress of 13.9 MPa, and pore fluid pressure of 5 MPa. The 6 subplots display the different variables of interest during the experimental run as a function of time. From top left to bottom right: external temperature of the *pressure vessel* (with sample temperature indicated in black). The wavy pattern is due to the rotation of the cell during tomography acquisition (fan blowing

on the thermocouple, cooling it for a few seconds). Confining pressure. LVDT signal, with the two drops related to metamorphic reactions (see below). Differential stress (where σ_1 is axial stress and σ_3 confining pressure). Ram fill level: the amount of fluid in the syringe applying pressure on the hydraulic actuator. Pore fluid pressure. Green inset 1) and red inset 2) details variation of ram fill level, LVDT (on the first larger panel) and pore fluid syringe fluid level for the gypsum to basanite and basanite to anhydrite reaction respectively. The signal indicates successive axial shortening of the sample going with a significant fluid released in the pore fluid channel.
