# Supplementary material for: Heitt Mjölnir: a heated miniature triaxial apparatus for 4D synchrotron microtomography
Source: J Synchrotron Radiat. 2024 Jan 1;31(Pt 1):150–61. doi: 10.1107/S1600577523009876 (PMC10833432; doi:10.1107/S1600577523009876)
Supplement: Supplementary file 2 [file s-31-00150-sup2.zip › flux _calculation_HM/Flux_calculation_HeittMjolnir.html]

Flux\_calculation\_HeittMjolnir


This notebook is part of the Supporting Information (SI) for Article:

# Heitt Mjölnir: heated miniature triaxial apparatus for 4D synchrotron microtomography

Damien Freitas\*1,2, Ian B. Butler1, Stephen C. Elphick1, James Gilgannon1, Roberto Rizzo1,3, Oliver Plümper4, John Wheeler5, Christian M. Schlepütz6, Federica Marone6 and Florian Fusseis1

1School of geosciences, University of Edinburgh, James Hutton Road, The King’s Buildings, Edinburgh EH9 3FE, United Kingdom  
2University of Manchester, Diamond Light Source, Harwell Campus, Didcot OX11 0DE, UK  
3Department of Earth Sciences, University of Florence, Via La Pira 4, 50121, Florence, IT  
4Department of Earth Sciences, Utrecht University, Budapestlaan 4, 3584CD Utrecht, NL  
5Department of Earth, Ocean and Ecological Sciences, University of Liverpool, 4 Brownlow Street, Liverpool L69 3GP, UK  
6Swiss Light Source, Paul Scherrer Institute, Forschungsstrasse 111, 5232 Villigen PSI, CH

---

# X-ray flux and spectrum calculations¶

This notebook details the X-ray flux and spectrum calculations for experiments with the Heitt Mjölnir rig at the TOMCAT beamline, Swiss Light Source. The code can be modified as needed to simulate other experimental setups.

The flux and spectrum calculations are performed in three steps, as outlined below.

## Notes on nomenclature¶

For consistent interpretation of the data, we define here the nomenclature used throughout the notebook:

**Flux distribution (2D+1D)**: The flux values resolved both in energy and in spatial coordinates per unit energy and unit area [ph/s/eV/mm^2] -> *F\_mesh*   
**Flux density profile (2D)**: The flux values resolved in space and integrated over all energies per unit area, i.e. the spatial cross-section of the beam with all energies [ph/s/mm^2] -> *Fd\_profile*  
**Flux spectrum (1D)**: The flux values resolved in energy and integrated over both spatial coordinates per unit energy [ph/s/eV] -> *F\_spectrum*  
**Flux density spectrum (1D)**: The flux values resolved in energy and averaged in the spatial coordinate, normalized to area units [ph/s/eV/mm^2] -> *Fd\_spectrum*  
**Flux (0D)**: The flux distribution integrated over space and energy to give a single intensity value [ph/s] -> *F*  
**Flux density (0D)**: The flux distribution integrated in energy and over space, normalized to area units [ph/s/mm^2] -> *Fd*

## 1. Source calculation¶

The full X-ray energy spectrum of the TOMCAT bending magnet source is calculated for the field of view employed in the experiments. Note that due to the finite size of the field of view in the vertical direction, there is a noticable flux decay away from the orbital plane of the synchrotron, which is more significant for hard X-rays at high energies. The "raw" source calculations are denoted by ***F0***, and do not yet take into account any absorption effects by the various materials along the beam.

## 2. Material transmission calculations¶

In this step, all of the materials used in the experiment are specified. This includes the penetrated beamline components (windows, air), the filter materials used to shape the X-ray spectrum, the materials wich make up the Mjölnir rig itself as well as the sample to be investigated, and finally the scintillator material used to detect the X-rays. For each material and component, the energy-dependent X-ray transmission is then calculated and plotted.

## 3. Effect of material transmission on the spectrum¶

This section combines the raw spectrum from step 1 and the transmission calculations from step 2. It computes the effects of the different materials introduced into the beam onto the transmitted spectrum to predict the beam profile, the overall transmitted flux and transmitted and absorbed power.

---

# 0. Preparatory work¶

Configure the notebook, import libraries, define necessary variables and functions.

In [1]:

```
# Import libraries

import matplotlib.pyplot as plt
import numpy as np
import pandas as pd
import xraydb as xdb
import xrt.backends.raycing.sources as rs
```

In [2]:

```
# Configure the workspace

%matplotlib widget
pd.options.display.float_format = '{:.3g}'.format
```

In [3]:

```
# Define variables

eV2J = 1.602176634e-19
```

In [4]:

```
# Define functions and classes

def material_transmission(material, thickness, energy, density=None):
    """
    Calculate the energy-dependent transmission of a material.
    
    Parameters
    ----------
    material : str
        The filter material, e.g.: 'Fe'
    thickness : float
        The material thickness in mm
    energy : array-like float
        Array of X-ray energies in eV for which the transmission is to be calculated
    density : float
        The density of the material. If None, use tablulated values
        
    Returns
    -------
    trans : array-like float
        Transmission values at the specified energies

    """
    
    try:
        # This should work if it's an element (not listed as a named material in the database)
        rho = xdb.atomic_density(material)
        mu = xdb.mu_elam(material, energy) * rho
    except:
        # This should work if it's a material (listed in the materials database)
        mu = xdb.material_mu(material, energy, density)

    abs_len = 1.0e1 / mu
    trans = np.exp(-(thickness / abs_len))
    return trans


def material_absorption(material, thickness, energy, density=None):
    """
    Calculate the energy-dependent absorption of a material.
    
    Parameters
    ----------
    material : str
        The filter material, e.g.: 'Cu'
    thickness : float
        The material thickness in mm
    energy : array-like float
        Array of X-ray energies in eV for which the absorption is to be calculated
    density : float
        The density of the material. If None, use tablulated values
        
    Returns
    -------
    absorption : array-like float
        Absorption values at the specified energies

    """
    
    return (1 - material_transmission(material, thickness, energy, density=density))


class Component:
    """
    A class to combine different material slabs into a single experimental component
    """
    
    def __init__(self, label='', materials=[]):
        self.label = label
        self.materials = materials
        
    def add_material(self, material, thickness):
        self.materials.append((material, thickness))
        
    def set_label(self, label):
        self.label = label

        
def component_transmission(component, energy):
    """
    Calculate the total transmission of a component
    
    Parameters
    ----------
    component : object of class Component
        The component for which the transmission shall be calculated
    energy : array-like float
        Array of X-ray energies in eV for which the absorption is to be calculated
        
    Returns
    -------
    trans_tot : array-like float
        The components overall absorption values at the specified energies
        
    """
    
    trans_tot = np.ones(energy.shape)
    for mat, thick in component.materials:
        transm = material_transmission(mat, thick, energy)
        trans_tot = trans_tot * transm
    return trans_tot


def plot_component_transmission(component, energy, plot_total=True):
    """
    Plot the transmission curves for all of the materials making up a component
    
    Parameters
    ----------
    component : object of class Component
        The component for which the transmission curves shall be plotted
    energy : array-like float
        Array of X-ray energies in eV for which the absorption is to be plotted
    plot_total : bool, optional
        If set to True, add the combined total transmission of the component to the plot.
        (default = True)

    """
    
    plt.cla()
    trans_tot = np.ones(energy.shape)
    for mat, thick in component.materials:
        transm = material_transmission(mat, thick, energy)
        trans_tot = trans_tot * transm
        plt.plot(energy / 1000.0, transm, label=f'{thick:g} mm {mat}')
    if plot_total:
        plt.plot(energy / 1000.0, trans_tot, linewidth=3, color='k', label=f'total {component.label}', zorder=0)
    plt.legend(framealpha=0.7)
    plt.title(component.label)
    plt.ylabel("X-ray transmission")
    plt.xlabel("X-ray energy [keV]")
    plt.grid()
```

In [5]:

```
# Print version and runtime information
import datetime
import matplotlib
import sys
import xrt

py_version = ".".join(map(str, sys.version_info[:3]))

print(f"Execution date: {datetime.datetime.now()}")
print("")
print("Version information")
print("-------------------")
print(f"{'Python:':20s} {py_version}")
print(f"{'Matplotlib (plt):':20s} {matplotlib.__version__}")
print(f"{'Numpy (np):':20s} {np.__version__}")
print(f"{'Pandas (pd):':20s} {pd.__version__}")
print(f"{'XrayDB (xdb):':20s} {xdb.__version__}")
print(f"{'XrayTracer (xrt):':20s} {xrt.__version__}")
```

```
Execution date: 2023-08-09 11:24:56.866833

Version information
-------------------
Python:              3.11.4
Matplotlib (plt):    3.7.2
Numpy (np):          1.25.1
Pandas (pd):         2.0.3
XrayDB (xdb):        4.5.0
XrayTracer (xrt):    1.6.0
```

# 1. Source calculation¶

The source calculation is done by using the XrayTracer library xrt [1,2,3]. In this example, the parameters are set to match the TOMCAT beamline specifications.

[1] Konstantin Klementiev and Roman Chernikov "Powerful scriptable ray tracing package xrt", Proc. SPIE 9209, Advances in Computational Methods for X-Ray Optics III, 92090A (5 September 2014); https://doi.org/10.1117/12.2061400 [doi.org]   
[2] https://xrt.readthedocs.io/ [xrt.readthedocs.io]   
[3] https://doi.org/10.5281/zenodo.1252468 [doi.org]

## 1.1 Define the source parameters¶

The source parameters of the current TOMCAT beamline at the Swiss Light Source are as follows:

| Storage ring energy [GeV] | Storage ring current [A] | Superbend magnetic field strength [T] |
| --- | --- | --- |
| 2.4 | 400 | 2.9 |

Additionally, we define the distance of the point of interest (POI, i.e., the sample) from the source as well as the transverse (horizontal and vertical) size of the region-of-interest (ROI), which corresponds to field of view of the detector measured at the POI.

In [6]:

```
# Source parameters

# Energy range for the calculation [eV]
# (cannot start at zero as spectrum diverges)
energy = np.arange(1, 120001, 100)

# Storage ring energy [GeV] --> eE
storage_ring_energy = 2.4

# Storage ring electron current [A] --> eI
storage_ring_current = 0.4

# Bending magnet field strength [T] --> B0
B0 = 2.9

# The distance of the measurement point (POI) from the source [m]
source_distance = 25

# Horizontal and vertical ROI size at the POI [mm]
size_h = 5.0
size_v = 2.0
```

## 1.2 Run the source calculations¶

The source calculation are exectuted in the next few cells.

Calculate the full flux distribution, resolved both in energy and space, at the POI based on the above parameters. From the distribution, we can derive the other quantities (profile, spectrum, etc.)

In [7]:

```
# According to the xrt manual, the mesh needs to be computed on a larger grid than the FOV and then
# sliced to the correct size afterwards to avoid edge effects in the calculations. Here, we double it in
# both directions.

# calculate the horizontal acceptance of the ROI [rad]
# Use only very few points for the grid as the profile shape is independent of the horizontal opening angle.
npts_theta = 8
dtheta_dh = 1.0 / (source_distance * 1e3)
theta_range = size_h * dtheta_dh
theta = np.linspace(-theta_range, theta_range, 2 * npts_theta, endpoint=False)
dtheta = theta[1] - theta[0]
theta = theta + dtheta / 2.0
dh = dtheta / dtheta_dh

# calculate the vertical acceptance of the ROI [rad]
npts_psi = 60
dpsi_dv = 1.0 / (source_distance * 1e3)
psi_range = size_v * dpsi_dv
psi = np.linspace(-psi_range, psi_range, 2 * npts_psi, endpoint=False)
dpsi = psi[1] - psi[0]
psi = psi + dpsi / 2.0
dv = dpsi / dpsi_dv

# calculate step sizes in theta, psi, and energy
dE = energy[1] - energy[0]

# resulting flux in units per eV (and not in 0.1% bandwidth)
distE = 'eV'

# calculate the source spectral distribution for the above parameters at the POI [ph/s/eV/mm^2]
source = rs.BendingMagnet(eE=storage_ring_energy, eI=storage_ring_current, B0=B0, distE=distE)
F0_mesh = source.intensities_on_mesh(energy=energy, theta=theta, psi=psi)[0] * dtheta_dh * dpsi_dv

# cut out the central half of the mesh in both directions from the larger one used for the calculation to match the FOV
F0_mesh = F0_mesh[:, int(npts_theta/2):int(3*npts_theta/2), int(npts_psi/2):int(3*npts_psi/2)]

# From the distrubution, calculate the spectrum, profile, flux, flux density
F0_profile = F0_mesh.sum(axis=0) * dE
F0_spectrum = F0_mesh.sum(axis=(1, 2)) * dh * dv
Fd0_spectrum = F0_spectrum / (size_h * size_v)
F0 = np.sum(F0_spectrum) * dE
Fd0 = F0 / (size_h * size_v)

# Calculate also the power distribution, spectrum, profile, and density
energy_3d = np.tile(np.expand_dims(energy, axis=(1,2)), (1, F0_mesh.shape[1], F0_mesh.shape[2]))
P0_mesh = F0_mesh * energy_3d * eV2J
P0_spectrum = P0_mesh.sum(axis=(1,2)) * dh * dv
P0_dens_spectrum = P0_spectrum / (size_h * size_v)
P0_profile = P0_mesh.sum(axis=0) * dE
P0 = np.sum(P0_mesh.ravel()) * dE * dh * dv
P0_dens = P0 / (size_h * size_v)
```

## 1.3 Output some basic information about the source flux and power through the ROI.¶

**Note:** The total number of photons calculated for the source is completely dominated by the low-energy contributions as the calculations diverge at E=0 (for the equidistant sampling in the energy domain that is employed here). So the numbers shown here for the source are to be interpreted with care! As soon as this spectrum propagates through the beamline, the entire low-energy part of the spectrum is lost immediately due to absorption.

In [8]:

```
print("Region of interest (ROI): {} mm x {} mm @ {} m".format(size_h, size_v, source_distance))
print("")

# Print flux info
print("Integrated flux over ROI from BM [ph/s]: {:.03g}".format(F0))
print("Average flux density over ROI [ph/s/mm^2]: {:.03g}".format(Fd0))
print("Maximim flux density within ROI [ph/s/mm^2]: {:.03g}".format(F0_profile.max()))
print("Minimim flux density within ROI [ph/s/mm^2]: {:.03g}".format(F0_profile.min()))
print("")

# Print power info
print("Total power in ROI from BM [W]: {:.3g}".format(P0))
print("Average power density over ROI [W/mm^2] {:.3g}".format(P0_dens))
print("Maximim power density within ROI [W/mm^2]: {:.03g}".format(P0_profile.max()))
print("Minimim flux density within ROI [W/mm^2]: {:.03g}".format(P0_profile.min()))
```

```
Region of interest (ROI): 5.0 mm x 2.0 mm @ 25 m

Integrated flux over ROI from BM [ph/s]: 2.57e+15
Average flux density over ROI [ph/s/mm^2]: 2.57e+14
Maximim flux density within ROI [ph/s/mm^2]: 2.59e+14
Minimim flux density within ROI [ph/s/mm^2]: 2.55e+14

Total power in ROI from BM [W]: 3.27
Average power density over ROI [W/mm^2] 0.327
Maximim power density within ROI [W/mm^2]: 0.334
Minimim flux density within ROI [W/mm^2]: 0.314
```

## 1.4 Plot the flux density spectrum¶

The flux density spectrum averaged over the ROI is plotted below.

On the left axis (blue curve), the flux spectrum is plotted in the units used throughout this notebook: Values are normalized in the energy dimension per eV. As can be seen in the plot, the values diverge as the energy approaches zero. This is the reason why integrated flux values are difficult to interpret, because the value very sensitively depends on the integration limits, and zero energy needs to be excluded.

The red curve (right y-axis) is plotted in the much more customary units normalizing the energy dimension as per 0.1% bandwidth. While these units are convenient when dealing with bandwith-limited optical components, the linear scaling in eV is much more directly applicaple to the calculations performed on the whole spectrum.

In [9]:

```
Fd0_spectrum_bw = Fd0_spectrum * energy / 1000

fig, ax1 = plt.subplots()
ax1.semilogy(energy/1000.0, Fd0_spectrum, color='C0', linewidth=3, label='normalized per eV')
ax1.set_xlabel('Energy [keV]')
ax1.set_ylabel('Flux density in ROI [ph/s/eV/mm^2]', color='C0')
ax1.tick_params(axis='y', colors='C0')
plt.legend(loc='lower left')
plt.ylim(1e5, 1e11)
plt.grid()
ax2 = ax1.twinx()
ax2.semilogy(energy/1000.0, Fd0_spectrum_bw, color='r', linewidth=3, label='normalized per 0.1% BW')
ax2.set_ylabel('Flux density in ROI [ph/s/0.1%BW/mm^2]', color='r')
ax2.tick_params(axis='y', colors='r')
plt.legend(loc='upper right')
plt.title("Flux density spectra plotted in different units")
plt.ylim(1e5, 1e11)
plt.show()
```

Figure

# 2. Transmission calculations¶

Filter transmission and scintillator absorption calculations are carried out using the XrayDB library [1,2]

Note: a nice web-interface to the XrayDB data is available here: https://xraydb.xrayabsorption.org/ [xraydb.xrayabsorption.org]

[1] https://xraypy.github.io/XrayDB/ [xraypy.github.io]  
[2] https://doi.org/10.5281/zenodo.7574458 [doi.org]

## 2.1 Define filter and scintillator materials¶

In general, one needs to define three parameters for each filter/scintillator:

1. The **material**: The atomic composition (chemical formula) of the material to be used.
2. The **thickness**: The thickness of the material along the beam direction [mm].
3. The **density**: The density of the material [g/cm3].

A large number of materials is already preconfigured in the XrayDB library's database. Here, we add specifically those that are relevant for the Heitt Mjölnir experiments to the database.

In [10]:

```
# Add custom materials to the list here.
# Entries are tuples that contain four fields:
#      1.  common name
#      2.  chemical formula (in elemental molar ratios)
#      3.  density in gr/cm^3
#      4.  comma delimited list of categories

materials = [
    # Name, Formula, Density, Categories
    ('Sigradur G glassy carbon', 'C', 1.42, ['element']),
    ('CVD diamond', 'C', 3.52, ['element']),
    ('borosilicate glass', '(B2O3)0.12(SiO2)0.88', 2.51, ['glass']),
    ('Al 7068', 'Al1.000Cu0.010Mg0.033Zn0.037', 2.85, ['alloy']),
    ('Cu C101', 'Cu', 8.92, ['element']),
    ('graphite 6507', 'C', 1.74, ['element']),
    ('PEEK', 'C19H14O3', 1.32, ['polymer']), 
    ('gypsum', 'CaSO4(H2O)2', 2.36, ['rock']),
    ('LuAG', 'Al5Lu3O12', 6.71, ['ceramic', 'scintillator']),
    ('SYLTHERM 800 oil', 'CH3(Si(CH3)2O)4Si(CH3)3', 0.936, ['polymer'])
]

# Add the materials to the database
for material in materials:
    name, formula, density, categories = material
    if not xdb.find_material(name):
        xdb.add_material(name, formula, density, categories)
```

## 2.2 Specify the materials of different components of the experiment¶

In [11]:

```
# Define different experiment components and list their materials and thicknesses

BL = Component(
    label="Beamline",
    materials=[
        ("CVD diamond", 0.2),
        ("kapton", 0.3),
        ("air", 2800),
    ]
)

BL_filters = Component(
    label="Filters",
    materials=[
        ("Sigradur G glassy carbon", 20.0),
        ("Mo", 0.35),
        ("borosilicate glass", 12.0),
    ]
)

HM_rig = Component(
    label="Heitt Mjölnir rig",
    materials=[
        ("Al 7068", 10.0),
        ("Cu C101", 0.14),
        ("graphite 6507", 4.3),
        ("PEEK", 6.0),
        ("SYLTHERM 800 oil", 1.0),
    ]
)

HM_sample = Component(
    label="Gypsum sample",
    materials=[
        ("gypsum", 10.0),
    ]
)

Scintillator = Component(
    label="Scintillator",
    materials=[
        ("LuAG", 0.15),
    ]
)
```

## 2.3 Ordering of components¶

Now we define the order in which the effect of the components is calculated. This is, in essence, their order along the beam path. However, this is not strictly true, as for example the sample is placed inside the the rig, with half of the rig before the sample, and the other half behind.

For the components individual transmission or the aggregate transmission calculations through all components, the order is not important as the multiplication of the spectrum with the transmission values is commutative. However, for the transmitted flux of each individual component, the order matters due to beam hardening effects of the continuous flux distribution caused by previous components.

For the power absorbed by each component, the order is also relevant, as downstream components are "shielded" by the more upstream components.

Hence, the values calculated here should be taken as approximations and ball-park figures, which is often enough for a first evaluation. If exact numbers are to be calculated, then the exact order of components and possibly even materials within the components would need to be reflected in the component definitions.

In [12]:

```
# Define the order in which the components appear along the beam direction
component_order = [BL, BL_filters, HM_rig, HM_sample]
```

## 2.4 Plot the X-ray transmission values of different components¶

In [13]:

```
fig, axs = plt.subplots(nrows=1, ncols=len(component_order), sharey=True, figsize=[4*len(component_order),4])

for ind, component in enumerate(component_order):
    plt.sca(axs[ind])
    plot_component_transmission(component, energy)
plt.ylim(0,1)
fig.suptitle("Energy-dependent X-ray transmission curves", fontsize=16)
fig.tight_layout()
plt.show()

plt.savefig('transmission_curves.pdf', transparent=True)
```

Figure

## 2.5 Calculate and plot the X-ray absorption of the scintillator¶

The X-ray absorption of the scintillator crystal is used as an indicator for its detection efficiency for X-rays at different energies. Note that this is not a direct measurment of of the actual light yield and cannot be used to quantitatively predict the intensity levels recorded by the camera, but it nevertheless provides some information about how much of the incoming flux is actually detectable (all X-rays transmitted through the scintillator without energy loss are surely not detectable by the visible light camera system).

In [14]:

```
plt.figure()
mat, thick = Scintillator.materials[0]
scintillator_absorption = material_absorption(mat, thick, energy)
plt.plot(energy / 1000.0, scintillator_absorption, label=f'{thick:g} mm {mat}')
plt.legend(framealpha=0.5)
plt.title(Scintillator.label)
plt.xlabel("X-ray energy [keV]")
plt.ylabel("X-ray absorption")
plt.grid()
plt.show()
```

Figure

# 3. Transmitted and detected X-ray spectrum and flux¶

## 3.1 Calculate the cumulative spectrum information¶

The spectrum, flux, profile, intensity, and power values are calculated from the source through all of the subsequent components.

In [15]:

```
component_calculations = {}
aggregate_transmission = np.ones(energy.shape)
F_spectrum = F0_spectrum
Fd0_spectrum_onaxis = F0_mesh[:,0,int(F0_mesh.shape[2]/2.0)]
Fd0_spectrum_offaxis = F0_mesh[:,0,0]

# expand the calculated scintillator absorption to the array size of the energy-resolved flux profile (F0_mesh)
scintillator_absorption_3d = np.tile(np.expand_dims(scintillator_absorption, axis=(1,2)), (1, F0_mesh.shape[1], F0_mesh.shape[2]))

# Loop over all components in succession to calculate their impact on the beam spectrum, flux, flux_profile, integrated intensity and energy
# The results are stored in a dictionary using the component labels as keys.
for component in component_order:
    individual_transmission = component_transmission(component, energy)
    individual_absorption = 1 - individual_transmission
    
    aggregate_transmission = aggregate_transmission * individual_transmission
    aggregate_transmission_3d = np.tile(np.expand_dims(aggregate_transmission, axis=(1,2)), (1, F0_mesh.shape[1], F0_mesh.shape[2]))
    F_mesh = F0_mesh * aggregate_transmission_3d
    F_mesh_detected = F_mesh * scintillator_absorption_3d
    
    # calculate the absorbed part of the spectrum from the previous iteration spectrum and the component absorption
    # (make sure to update F_spectrum only afterwards)
    F_spectrum_abs = F_spectrum * individual_absorption
    Fd_spectrum_abs = F_spectrum_abs / (size_h * size_v)
    
    # calculate the transmitted flux and flux density spectra
    F_spectrum = F0_spectrum * aggregate_transmission
    Fd_spectrum = F_spectrum  / (size_h * size_v)
    Fd_spectrum_onaxis = Fd0_spectrum_onaxis * aggregate_transmission
    Fd_spectrum_offaxis = Fd0_spectrum_offaxis * aggregate_transmission
    
    # calculate the detectable part of the transmitted flux and flux density spectra
    F_spectrum_detected = F_spectrum * scintillator_absorption
    Fd_spectrum_detected = Fd_spectrum * scintillator_absorption
    Fd_spectrum_detected_onaxis = Fd_spectrum_onaxis * scintillator_absorption
    Fd_spectrum_detected_offaxis = Fd_spectrum_offaxis * scintillator_absorption
    
    # calculate the spatially resolved flux profile and its detectable part
    F_profile_trans = F_mesh.sum(axis=0) * dE
    F_profile_detected = F_mesh_detected.sum(axis=0) * dE
    
    # calculate the number of transmitted, absorbed, and detected photons by the component
    F_trans = np.sum(F_spectrum) * dE
    F_abs = np.sum(F_spectrum_abs) * dE
    F_detected = np.sum(F_spectrum_detected) * dE
    
    # calculate the transmitted, absorbed, and detectable beam power by the component
    P_trans = np.sum(F_spectrum * energy) * dE * eV2J
    P_abs = np.sum(F_spectrum_abs * energy) * dE * eV2J
    P_detected = np.sum(F_spectrum_detected * energy) * dE * eV2J
    
    component_calculations[component.label] = {
        'individual_transmission': individual_transmission,
        'aggregate_transmission': aggregate_transmission,
        'F_spectrum': F_spectrum,
        'F_spectrum_absorbed': F_spectrum_abs,
        'F_spectrum_detected': F_spectrum_detected,
        'Fd_spectrum': Fd_spectrum,
        'Fd_spectrum_onaxis': Fd_spectrum_onaxis,
        'Fd_spectrum_offaxis': Fd_spectrum_offaxis,
        'Fd_spectrum_detected': Fd_spectrum_detected,
        'Fd_spectrum_detected_onaxis': Fd_spectrum_detected_onaxis,
        'Fd_spectrum_detected_offaxis': Fd_spectrum_detected_offaxis,
        'F_transmitted': F_trans,
        'F_absorbed': F_abs,
        'F_detected': F_detected,
        'F_profile_transmitted': F_profile_trans,
        'F_profile_detected': F_profile_detected,
        'P_transmitted': P_trans,
        'P_absorbed': P_abs,
        'P_detected': P_detected,
    }
```

## 3.2 Plot the flux density spectra transmitted through the components¶

Solid lines indicate the energy-dependent average flux density values after each component. The shaded area around the solid lines corresponds to the spatial variation in flux density from the on-axis position (highest value) to the uppermost or lowermost off-axis position away from the orbital plane of the synchrotron. The dashed lines correspond to the portion of these flux curves which are actually absorbed by the scintillator crystal and can hence be detected in the experiment (they would be subject to the same intensity spread as indicated by the shaded areas).

In [16]:

```
# Plot the flux spectra
fig, axs = plt.subplots(nrows=1, ncols=2, figsize=[15,7.5])
fig.suptitle('Flux density spectra', fontsize=16)

# left: linear y-scale
plt.sca(axs[0])
plt.cla()
x = energy/1000.0
plt.plot(x, Fd0_spectrum, label='BM source')
for ind, component in enumerate(component_order):
    Fd_on = component_calculations[component.label]['Fd_spectrum_onaxis']
    Fd_off = component_calculations[component.label]['Fd_spectrum_offaxis']
    plt.plot(x, component_calculations[component.label]['Fd_spectrum'], label=component.label, color=f'C{ind+1}', linewidth=2)
    plt.plot(x, component_calculations[component.label]['Fd_spectrum_detected'], '--', color=f'C{ind+1}')
    plt.plot(x, Fd_on, ':', color=f'C{ind+1}', linewidth=0.75)
    plt.plot(x, Fd_off, ':', color=f'C{ind+1}', linewidth=0.75)
    plt.fill_between(x, Fd_on, Fd_off, alpha=0.2, color=f'C{ind+1}')
# dummy line to get one common label for the dotted curves 
plt.plot([0, 1], [2, 2], 'k--', label='detected fractions')
plt.xlim(x.min(), x.max())
plt.ylim(0, component_calculations['Filters']['Fd_spectrum'].max() * 1.1)
plt.xlabel("X-ray energy [keV]")
plt.ylabel("Transmitted X-ray flux density [ph/s/eV/mm^2]")
plt.legend()
plt.title("linear scale")
plt.grid()

# right: logarithmic y-scale
plt.sca(axs[1])
plt.cla()
plt.semilogy(x, Fd0_spectrum, label='BM source')
for ind, component in enumerate(component_order):
    Fd_on = component_calculations[component.label]['Fd_spectrum_onaxis']
    Fd_off = component_calculations[component.label]['Fd_spectrum_offaxis']
    plt.plot(x, component_calculations[component.label]['Fd_spectrum'], label=component.label, color=f'C{ind+1}', linewidth=2)
    plt.plot(x, component_calculations[component.label]['Fd_spectrum_detected'], '--', color=f'C{ind+1}')
    plt.plot(x, Fd_on, ':', color=f'C{ind+1}', linewidth=0.75)
    plt.plot(x, Fd_off, ':', color=f'C{ind+1}', linewidth=0.75)
    plt.fill_between(x, Fd_on, Fd_off, alpha=0.2, color=f'C{ind+1}')
# dummy line to get one common label for the dotted curves 
plt.plot([0, 1], [2, 2], 'k--', label='detected fractions')
plt.xlim(x.min(), x.max())
plt.ylim(100, component_calculations['Beamline']['Fd_spectrum'].max() * 10)
plt.xlabel("X-ray energy [keV]")
plt.ylabel("Transmitted X-ray flux density [ph/s/eV/mm^2]")
plt.legend()
plt.title("logarithmic scale")
plt.grid()
plt.savefig('flux_spectra.pdf', transparent=True)
plt.show()
```

Figure

## 3.3 Plot the progression of the beam profile¶

Due to the energy-dependent intensity decay in the vertical direction away from the synchrotron orbit, the measured beam profile is affected significantly by beam hardening and progressively narrows around the orbital plane as more and more of the low energy X-rays are absorbed by components in the beam.

In [17]:

```
# define functions to plot the beam profiles

def plot_beam_profile_img(profile, extent):
    plt.imshow(beam_profile.T, extent=extent, interpolation='bilinear', cmap='gray')
    plt.clim(0, beam_profile.max())
    #plt.axis('tight')
    #plt.axis('equal')
    ax = plt.gca()
    ax.set_aspect('equal', adjustable='box')
    ax.set_xticks([])
    ax.set_xticklabels([])
    ax.set_yticks([])
    ax.set_yticklabels([])    
    plt.xlabel('Horizontal extent')
    plt.ylabel('Vertical extent')
    plt.colorbar(label='Flux density')

def plot_beam_profile_vertical(profile, extent):
    bp = profile[0,:]
    x = np.linspace(extent[2], extent[3], len(bp))
    plt.plot(x, bp)
    plt.ylim([0, bp.max()*1.1])
    plt.xlabel('Vertical position [mm]')
```

### 3.3.1 Transmitted beam profiles¶

In [18]:

```
# plot the series of transmitted beam profiles

beam_profile = F0_mesh.sum(axis=0) * dE
n_components = len(component_order)

fig, axs = plt.subplots(nrows=2, ncols=n_components+1, figsize=[3 * (n_components + 1), 5], gridspec_kw={'height_ratios': [1, 2]})
fig.suptitle('Transmitted beam profiles', fontsize=16)
plt.sca(axs[0,0])
extent = [-size_h/2.0, size_h/2.0, -size_v/2.0, size_v/2.0]
plot_beam_profile_img(beam_profile, extent)
plt.title('BM source')
plt.sca(axs[1,0])
plot_beam_profile_vertical(beam_profile, extent)
plt.ylabel('Flux density [ph/s/mm^2]')

for ind, component in enumerate(component_order):
    beam_profile = component_calculations[component.label]['F_profile_transmitted']
    plt.sca(axs[0,ind+1])
    plot_beam_profile_img(beam_profile, extent)
    plt.title(component.label)
    plt.sca(axs[1,ind+1])
    plot_beam_profile_vertical(beam_profile, extent)

fig.tight_layout()
plt.show()
```

Figure

### 3.3.2 Detected beam profiles¶

In these plots, the detection efficiency of the scintillator is taken into account to approximate the detected beam profile as is would appear on the camera. Note that we do not take into account energy-dependent light yield, etc., but simply use the scintillator absorption as a proxy for its detection efficiency.

In [19]:

```
# plot the series of detected beam profiles
beam_profile = F0_mesh.sum(axis=0) * dE
n_components = len(component_order)

fig, axs = plt.subplots(nrows=2, ncols=n_components+1, figsize=[3 * (n_components + 1), 5], gridspec_kw={'height_ratios': [1, 2]})
fig.suptitle('Detected beam profiles', fontsize=16)
plt.sca(axs[0,0])
extent = [-size_h/2.0, size_h/2.0, -size_v/2.0, size_v/2.0]
plot_beam_profile_img(beam_profile, extent)
plt.title('BM source')
plt.sca(axs[1,0])
plot_beam_profile_vertical(beam_profile, extent)
plt.ylabel('Flux density [ph/s/mm^2]')

for ind, component in enumerate(component_order):
    beam_profile = component_calculations[component.label]['F_profile_detected']
    plt.sca(axs[0,ind+1])
    plot_beam_profile_img(beam_profile, extent)
    plt.title(component.label)
    plt.sca(axs[1,ind+1])
    plot_beam_profile_vertical(beam_profile, extent)

fig.tight_layout()
plt.show()
```

Figure

## 3.4 Calculate the integrated flux values¶

The flux profiles are integrated over the field of view and over all energies to give the number of photons passing through or being absorbed by each component

In [20]:

```
# Gather the flux intensity information from all components and the beamline
n = len(component_order)
F_transmitted = np.zeros(n+1)
F_transmitted[0] = F0
F_detected = np.zeros(n+1)
F_detected[0] = np.sum(F0_spectrum * scintillator_absorption) * dE
F_absorbed = np.zeros(n)
component_labels = ["BM Source", ]
header_labels = ["absolute Flux [ph/s]", "% BM Source", ]

for ind, component in enumerate(component_order):
    F_transmitted[ind+1] = component_calculations[component.label]['F_transmitted']
    F_detected[ind+1] = component_calculations[component.label]['F_detected']
    F_absorbed[ind] = component_calculations[component.label]['F_absorbed']
    component_labels.append(component.label)
    header_labels.append(f"% of {component.label}")
    
# Calculate the relative flux intensities
F_trans_rel = np.zeros([n+1, n+1])
F_det_rel = np.zeros([n+1, n+1])
F_abs_rel = F_absorbed / F0 * 100
for i in range(n+1):
    F_trans_rel[:, i] = F_transmitted / F_transmitted[i] * 100
    F_det_rel[:, i] = F_detected / F_detected[i] * 100
```

### 3.4.1 Transmitted flux¶

The integrated flux transmitted through each component is calculated (1st column). Additionally, the fraction of that flux compared to the flux after any of the other components is given in the following columns of the table as a percentage. Calculations are performed by integrating the flux over the entire cross-section of the ROI, so they represent the total number of photons reaching the sample, but are not normalized per unit area.

According to this table, the transmission of the Heitt Mjölnir rig is about 21.7% of the beam provided after the filters (:= 100%, corresponding to the flat field images). Adding the sample, the total transmission goes to about 11.2% of the filtered beam in the flat field.

Another striking observation is that from the total flux available from the beamline (after taking windows and air absorption into account), only about 0.28% are used in the experiments, the rest (predominantly lower energy photons) it taken out by the filters!

In [21]:

```
# Output table with absolute and relative transmitted flux information    
F_trans_data = np.hstack((F_transmitted[:, np.newaxis], F_trans_rel))
df = pd.DataFrame(data=F_trans_data, columns=header_labels, index=component_labels)
display(df)
```

|  | absolute Flux [ph/s] | % BM Source | % of Beamline | % of Filters | % of Heitt Mjölnir rig | % of Gypsum sample |
| --- | --- | --- | --- | --- | --- | --- |
| BM Source | 2.57e+15 | 100 | 594 | 2.07e+05 | 9.56e+05 | 1.85e+06 |
| Beamline | 4.33e+14 | 16.8 | 100 | 3.49e+04 | 1.61e+05 | 3.11e+05 |
| Filters | 1.24e+12 | 0.0482 | 0.286 | 100 | 461 | 891 |
| Heitt Mjölnir rig | 2.69e+11 | 0.0105 | 0.0622 | 21.7 | 100 | 193 |
| Gypsum sample | 1.39e+11 | 0.00541 | 0.0321 | 11.2 | 51.7 | 100 |

### 3.4.2 Detected transmitted flux¶

The fraction of the transmitted flux that can be detected by the scintillator is a viable proxy to estimate roughly the intensity changes observed by the camera system in the experiment.

Compared to the table above, the numbers for transmitted flux by the rig (22.1%) and sample flux (11.5%) with respect to the flat field illumination after the filters (:= 100%) have not changed much.

In [22]:

```
# Output table with absolute and relative detected transmitted flux information    
F_det_data = np.hstack((F_detected[:, np.newaxis], F_det_rel))
df = pd.DataFrame(data=F_det_data, columns=header_labels, index=component_labels)
display(df)
```

|  | absolute Flux [ph/s] | % BM Source | % of Beamline | % of Filters | % of Heitt Mjölnir rig | % of Gypsum sample |
| --- | --- | --- | --- | --- | --- | --- |
| BM Source | 2.5e+15 | 100 | 678 | 6.08e+05 | 2.75e+06 | 5.29e+06 |
| Beamline | 3.69e+14 | 14.7 | 100 | 8.97e+04 | 4.05e+05 | 7.81e+05 |
| Filters | 4.11e+11 | 0.0164 | 0.111 | 100 | 452 | 871 |
| Heitt Mjölnir rig | 9.1e+10 | 0.00364 | 0.0247 | 22.1 | 100 | 193 |
| Gypsum sample | 4.72e+10 | 0.00189 | 0.0128 | 11.5 | 51.9 | 100 |

### 3.4.3 Absorbed flux by component¶

The next table shows the total number of photons and the fraction of the flux that is absorbed by every component.

The fact that nearly 83% of the flux provided by the source do not make it even through the beamline itself is related to the source flux diverging at low energies (if plotted in units of eV rather than the usual 0.1% bandwidth). So this number is somewhat meaningless for the experiments and depends a lot on which low energy ranges the spectrum calculation includes. Also, as mentioned above, the exact ordering of components is relevant for these calculations, so the numbers indicated here are rather approximate, but give a good indication as to the general order of magnitudes.

In [23]:

```
F_abs_data = np.vstack((F_absorbed, F_abs_rel)).T
df = pd.DataFrame(data=F_abs_data,
                  columns=["absolute absorbed Flux [ph/s]", "relative absorption [%]"], index=component_labels[1:])
display(df)
```

|  | absolute absorbed Flux [ph/s] | relative absorption [%] |
| --- | --- | --- |
| Beamline | 2.14e+15 | 83.2 |
| Filters | 4.32e+14 | 16.8 |
| Heitt Mjölnir rig | 9.72e+11 | 0.0378 |
| Gypsum sample | 1.3e+11 | 0.00505 |

## 3.5 Calculate the transmitted and absorbed power by component¶

The total energy flux through the entire ROI is estimated from the energy spectrum.

In [24]:

```
# Gather the power information from all components and the beamline
n = len(component_order)
P_transmitted = np.zeros(n+1)
P_absorbed = np.zeros(n)
P_transmitted[0] = P0

header_labels[0] = "absolute transmitted Power [W]"
for ind, component in enumerate(component_order):
    P_transmitted[ind+1] = component_calculations[component.label]['P_transmitted']
    P_absorbed[ind] = component_calculations[component.label]['P_absorbed']

# Calculate the relative flux intensities
P_trans_rel = np.zeros([n+1, n+1])
P_abs_rel = P_absorbed / P0 * 100
for i in range(n+1):
    P_trans_rel[:, i] = P_transmitted / P_transmitted[i] * 100
```

### 3.5.1 Transmitted power¶

In [25]:

```
# Output table with absolute and relative (transmitted) flux information    
P_trans_data = np.hstack((P_transmitted[:, np.newaxis], P_trans_rel))
df = pd.DataFrame(data=P_trans_data, columns=header_labels, index=component_labels)
display(df)
```

|  | absolute transmitted Power [W] | % BM Source | % of Beamline | % of Filters | % of Heitt Mjölnir rig | % of Gypsum sample |
| --- | --- | --- | --- | --- | --- | --- |
| BM Source | 3.27 | 100 | 199 | 2.49e+04 | 1.04e+05 | 1.94e+05 |
| Beamline | 1.64 | 50.1 | 100 | 1.25e+04 | 5.19e+04 | 9.71e+04 |
| Filters | 0.0131 | 0.401 | 0.8 | 100 | 415 | 777 |
| Heitt Mjölnir rig | 0.00316 | 0.0965 | 0.193 | 24.1 | 100 | 187 |
| Gypsum sample | 0.00169 | 0.0517 | 0.103 | 12.9 | 53.5 | 100 |

### 3.5.2 Absorbed power¶

The power absorbed in each component is calculated.

The numbers indicate that the total power absorbed by the rig and the sample are absolutely negligible and in the mW range for the full ROI.

In [26]:

```
P_abs_data = np.vstack((P_absorbed, P_abs_rel)).T
df = pd.DataFrame(data=P_abs_data, columns=["absolute absorbed Power [W]", "relative Power absorption [%]"], index=component_labels[1:])
display(df)
```

|  | absolute absorbed Power [W] | relative Power absorption [%] |
| --- | --- | --- |
| Beamline | 1.63 | 49.9 |
| Filters | 1.63 | 49.7 |
| Heitt Mjölnir rig | 0.00995 | 0.305 |
| Gypsum sample | 0.00147 | 0.0449 |

---

# Appendix¶

## A. Comparing the transmission of different copper sleeves¶

Two versions of the copper sleeves were used in the experiments. The one used at TOMCAT has a wall thickness of 70 um (total thickness along the beam of 140 um), while the other one used at PSICHÉ had a wall thickness of 200 um (total thickness of 400 um). The following plot compares the X-ray transmission curves of the two different versions.

In [27]:

```
Cu = Component(
    label="Copper sleeves",
    materials=[
        ("Cu", 0.14),
        ("Cu", 0.40),
    ]
)
```

In [28]:

```
plt.figure()
plot_component_transmission(Cu, energy, plot_total=False)
plt.ylabel("X-ray Transmission")
plt.ylim(0,1)
plt.xlim(0,100)
plt.show()
```

Figure

# B. Experimental validation of the simulations¶

To validate the simulations above, they are compared to actually measured data during the experiments.

Note: The experimental data used in the comparison is not directly included as part of the supplementary article information. The code is provided, nevertheless, to facilitate similar validations for other experiments. One would simply have to adapt the corresponding code lines for loading the data files.

Original data sources:

- projections images: HM17\_Pf50\_Pc200\_R31\_005
- flat and dark field images: HM17\_Pf50\_Pc200\_R31\_refs\_000

## B.1 Beam profile of the flat field illumination¶

The simulated beam profile after the filters is compared to the measured beam profile in the acquired flat field images. The flat field image loaded here is the median of the last 50 (out of a total of 100) flat field images acquired for this scan. The flat field image is dark-field corrected before the comparison to subtract the constant dark field background of the camera that is not represented in the X-ray beam. The dark field image used here is the median of the 50 acquired dark field images of the scan.

It is evident from the plots that the prediction by the simulation matches the measured profile very well. Only the alignment of the detector with respect to the beam during the measurements was not perfect, resulting in a slight shift of the center of the beam profile towards the upper edge of the detector. The plot shows both the measured (orange) and the simulated (blue) beam profiles, normalized to their maximum values. Additionally, the simulated curve has been manually shifted to match the center of the measured profile (dashed line) to facilitate the direct comparison.

In [29]:

```
from PIL import Image
import matplotlib.gridspec as gridspec
import warnings
warnings.filterwarnings('ignore')

im_dark = np.array(Image.open('dark_image.tif'))
im_ff = np.array(Image.open('ff_image.tif'))
im_prj = np.array(Image.open('prj_image.tif'))

im_ff_corr = im_ff - im_dark
im_prj_corr = (im_prj - im_dark) / im_ff_corr

beam_profile_sim = component_calculations['Filters']['F_profile_detected'].T
v_profile_sim = beam_profile_sim[:,0]
x_sim = np.linspace(extent[2], extent[3], len(v_profile_sim))
v_profile_meas = np.sum(im_ff_corr, axis=1)
x_meas = np.linspace(extent[2], extent[3], len(v_profile_meas))

fig = plt.figure(figsize=[10,4])
fig.suptitle("Comparison between calculation and measurement")
gs = gridspec.GridSpec(2, 2)

# display the measured flat field image
ax = fig.add_subplot(gs[0, 0])
plt.sca(ax)
plt.imshow(im_ff_corr/im_ff_corr.ravel().max() , extent=extent, cmap='gray')
plt.clim(0, 1)
plt.title('measured flat field image')
plt.ylabel('vertical position [mm]')
ax.set_xticklabels([])
plt.colorbar(label='normalized intensity')
plt.vlines([0], -1, 1, linewidth=3, color='C0')

# display the calculated flat field image
ax = fig.add_subplot(gs[1, 0])
plt.sca(ax)
plt.imshow(beam_profile_sim / beam_profile_sim.ravel().max(), extent=extent, cmap='gray')
plt.clim(0, 1)
plt.title('calculated flat field image')
plt.ylabel('vertical position [mm]')
plt.xlabel('horizontal position [mm]')
plt.colorbar(label='normalized flux')
plt.vlines([0], -1, 1, linewidth=3, color='C1')

# compare the calculated to the measured vertical beam profile
ax = fig.add_subplot(gs[:, 1])
plt.sca(ax)
plt.plot(x_meas, v_profile_meas / v_profile_meas.max(), linewidth=3, color='C1', label='measurement')
plt.plot(x_sim, v_profile_sim / v_profile_sim.max(), linewidth=3, color='C0', label='calculation')
plt.ylabel('normalized vertical beam profile [a.u.]')
plt.xlabel('vertical position [mm]')
plt.title('Measured vs. calculated flat field beam profile')
plt.grid()
plt.plot(x_sim-0.143, v_profile_sim / v_profile_sim.max(), '--', color='C0', label='shifted calculation')
plt.legend()
fig.tight_layout()
plt.show()
plt.savefig('flux_comparison.pdf', transparent=True)
```

Figure

## B.2 Predicted transmission¶

The predicted transmission value of the sample and rig is compared to the measurements.

The projection image used here is an average of 80 equally spaced projection images throughout the 360 degree rotation range. Note that the center of the rig is located near the right image border due to the extended-field-of-view scanning mode of the 360-degree scans. The averaging helps to reduce local variations in transmission due to microstructure and gives a more representative measure of the transmission of the homogeneous material slabs, as they are assumed in the simulation. The projection image is dark- and flat-field corrected to result directly in the transmission values for each pixel.

The transmission values in a 200 pixel wide box around the center line of the rig are averaged over the complete image height to compare them to the simulated transmission values (which are also averaged over the full field of view).

The resulting measured transmission of 11.1% is very close to the predicted values of 11.2% for the transmitted flux and the 11.5% for the detected part of the transmitted flux, the latter being the more relevant number for the comparison.

In summary, the simulation was able to predict the measured values within a fraction of a percent, confirming that assumptions and approximations made throught the calculations are justified. The simulations prove to be a valid tool to aid in the experiment design and analysis.

In [30]:

```
# look at the average transmission of the rig plus sample around the center of the rig

from matplotlib.patches import Rectangle

center_x = 1850
roi_halfsize = 100

x1, x2 = center_x - roi_halfsize, center_x + roi_halfsize
center_y = int(im_prj_corr.shape[0] / 2.0)
y1, y2 = 0, im_prj_corr.shape[0]-1
roi = im_prj_corr[y1:y2, x1:x2]
trans = np.mean(roi[np.isfinite(roi)]) * 100
fig = plt.figure(figsize=[9, 3])
fig.suptitle("Measured transmission through the sample", fontsize=14)
plt.imshow(im_prj_corr, cmap='gray')
plt.clim(np.percentile(im_prj_corr[np.isfinite(im_prj_corr)], 0.1), np.percentile(im_prj_corr[np.isfinite(im_prj_corr)], 99.0))
plt.colorbar(label='transmission')
plt.title("Flat- and dark-field-corrected projection image")
ax = plt.gca()
plt.vlines([center_x], 0, im_prj_corr.shape[0]-1, linestyle='--', color='w')
plt.text(x1-50, 50, "Center line of the rig", color='w',
         fontsize=14, horizontalalignment='right', verticalalignment='top')
ax.add_patch(Rectangle((x1, y1), (x2-x1), (y2-y1), edgecolor = 'C1', fill=False, lw=3))
plt.text(x1-50, 400, f"Average transmission\nin rectangle: {trans:.1f}%", color='C1',
         fontsize=16, weight='bold', horizontalalignment='right', verticalalignment='center')
plt.tight_layout()
plt.show()
```

Figure
