# Supplementary material for: Heitt Mjölnir: a heated miniature triaxial apparatus for 4D synchrotron microtomography
Source: J Synchrotron Radiat. 2024 Jan 1;31(Pt 1):150–61. doi: 10.1107/S1600577523009876 (PMC10833432; doi:10.1107/S1600577523009876)

M6 breadboard

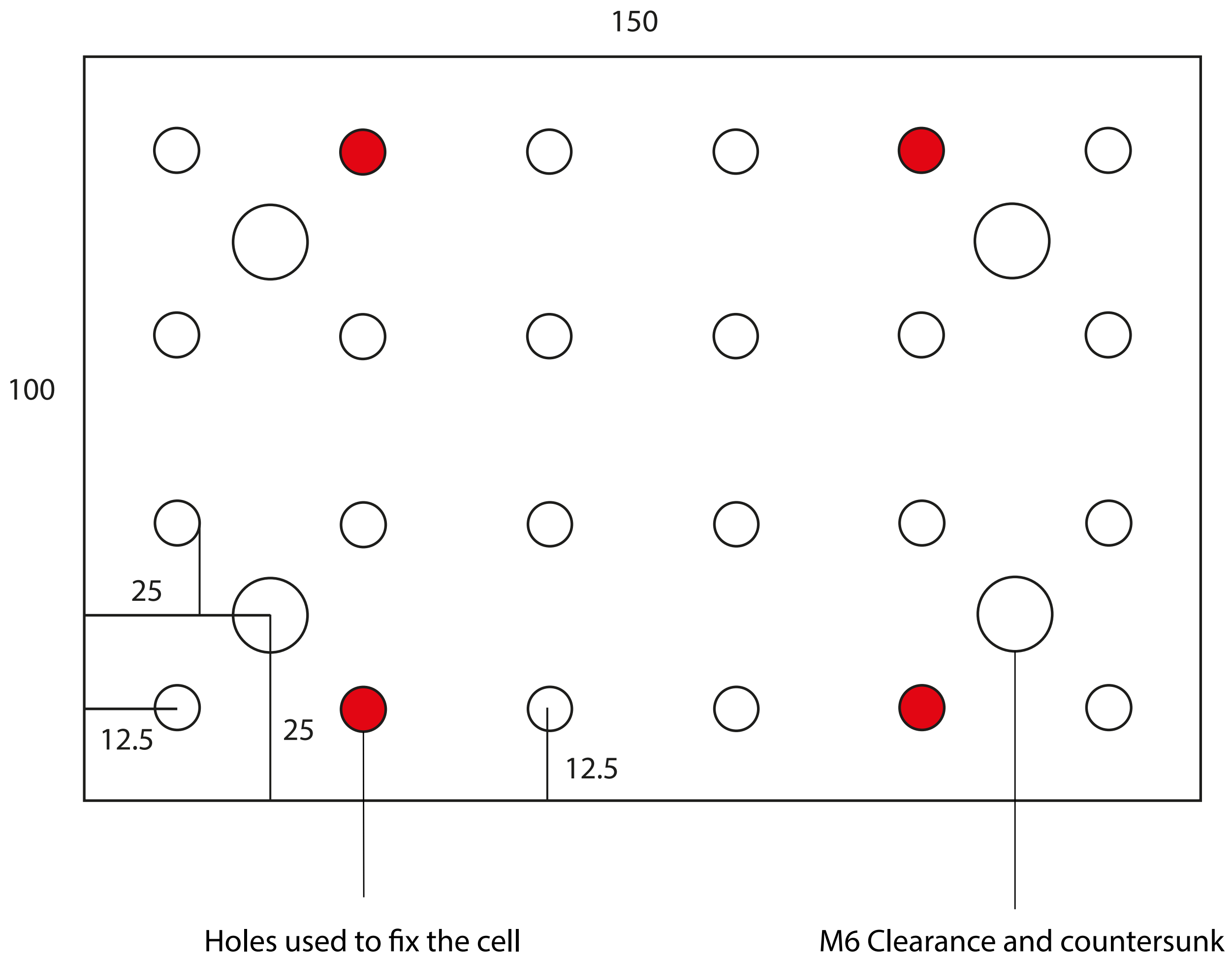

Unless otherwise specified dimensions are in millimetres

REV	DESCRIPTION THORLABS MB1015/M	NAME	DATE

Designed by D. Freitas & I. B. Butler<br>Date 31/08/2022		Draftsman D. Freitas<br>Customer F. Fusseis		Heat treatment / Surface treatment	Format A4<br>Scale 1 : 1
- ÷ XX	XX ÷ XXX	XXX ÷ XXXX	OVER XXXX	Material	Weight
±0.1	±0.2	±0.5	±1	Anodised Aluminium	Quantity

The University Of Edinburgh
School Of GeoSciences

Part code	Edition 00	Job code
Part name BREADBOARD		Sheet 1 / 1

The information contained in this drawing is the sole property of The University Of Edinburgh. Any reproduction in part or whole without the written permission of The University Of Edinburgh is prohibited

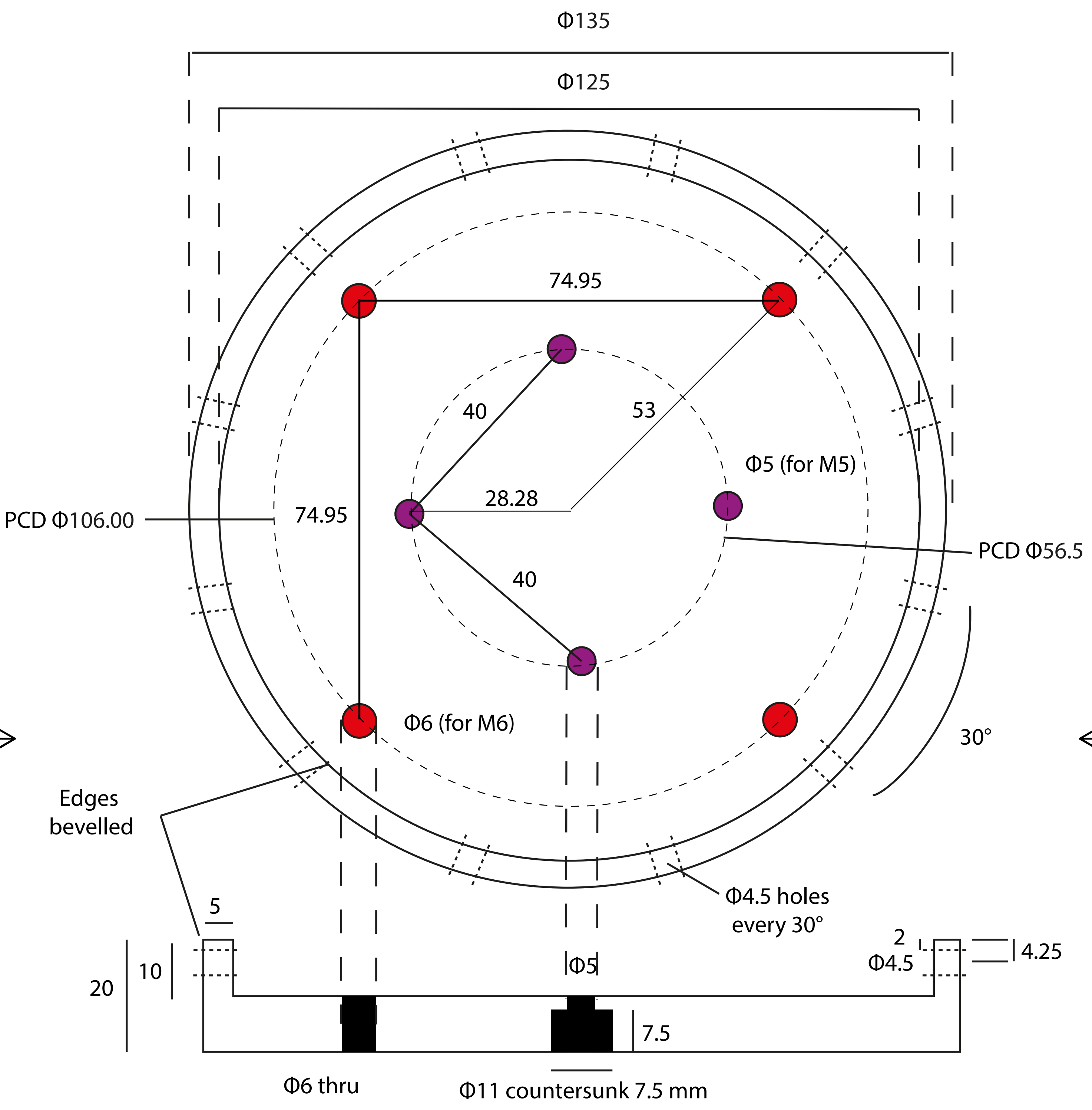

Unless otherwise specified dimensions are in millimetres

The information contained in this drawing is the sole property of The University Of Edinburgh. Any reproduction in part or whole without the written permission of The University Of Edinburgh is prohibited

REV	DESCRIPTION	NAME	DATE

Designed by<br>D. Freitas & I. B. Butler<br>Date<br>31/08/2022	Draftsman<br>D. Freitas<br>Customer<br>F. Fusseis	Heat treatment / Surface treatment	Format<br>A4<br>Scale<br>1 : 1

- ÷ XX	XX ÷ XXX	XXX ÷ XXXX	OVER XXXX	Material	Weight	Quantity
±0.1	±0.2	±0.5	±1	Aluminium 6000 series		

The University Of Edinburgh
School Of GeoSciences

Part code	Edition	Job code
	00	

Part name	Sheet
BASEPLATE	1 / 1

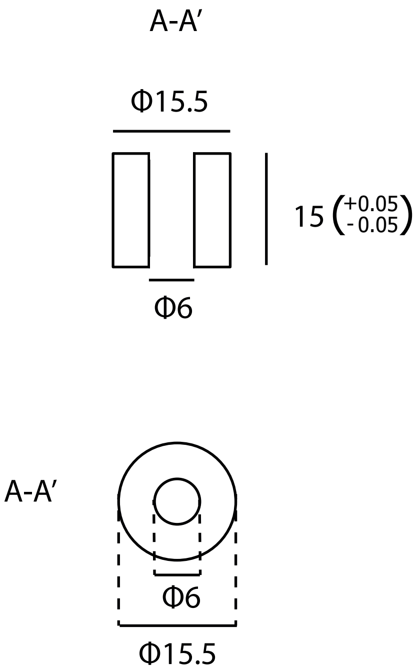

Unless otherwise specified dimensions are in millimetres

REV	DESCRIPTION	NAME	DATE

Designed by D. Freitas & I. B. Butler<br>Date 31/08/2022	Draftsman D. Freitas<br>Customer F. Fusseis	Heat treatment / Surface treatment	Format A4<br>Scale 1 : 1

- ÷ XX	XX ÷ XXX	XXX ÷ XXXX	OVER XXXX	Material	Weight	Quantity
±0.1	±0.2	±0.5	±1	PTFE	kg	

The University Of Edinburgh
School Of GeoSciences

Part code	Edition	Job code
	00	

Part name	Sheet
PTFE SPACER	1 / 1

The information contained in this drawing is the sole property of The University Of Edinburgh. Any reproduction in part or whole without the written permission of The University Of Edinburgh is prohibited

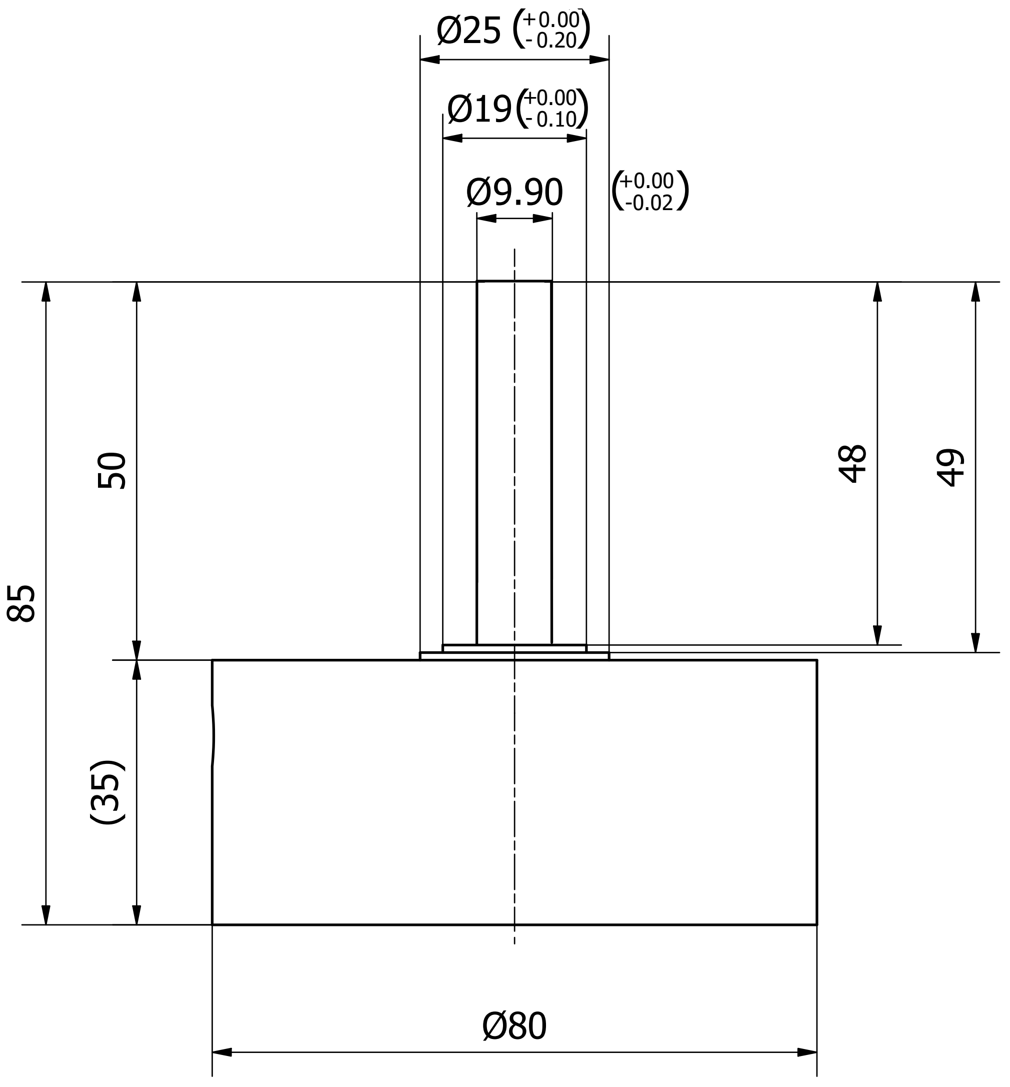

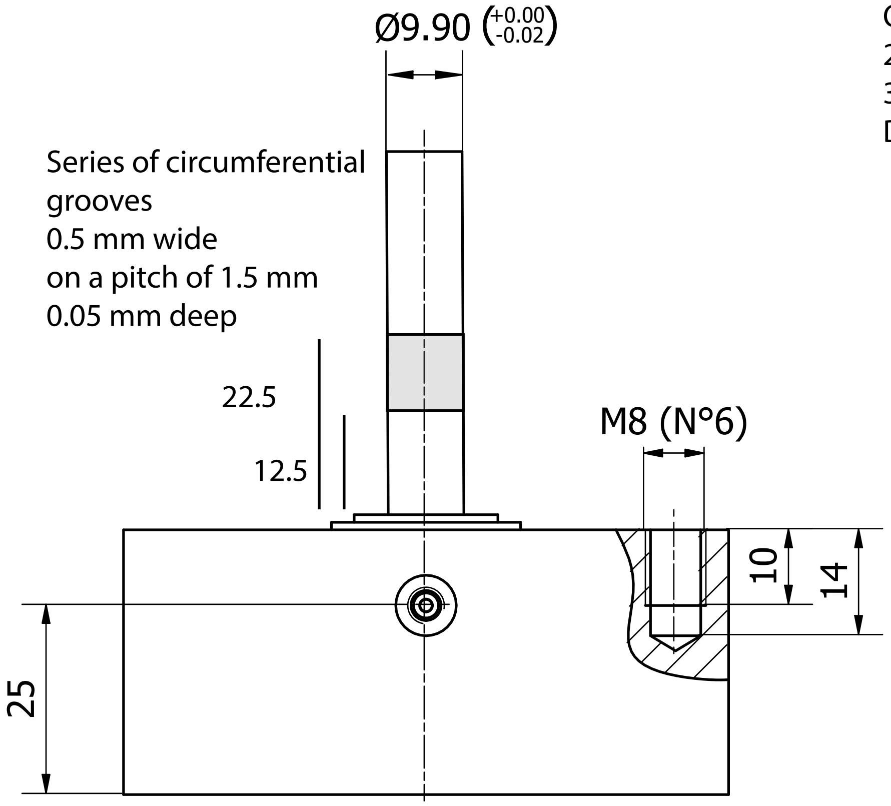

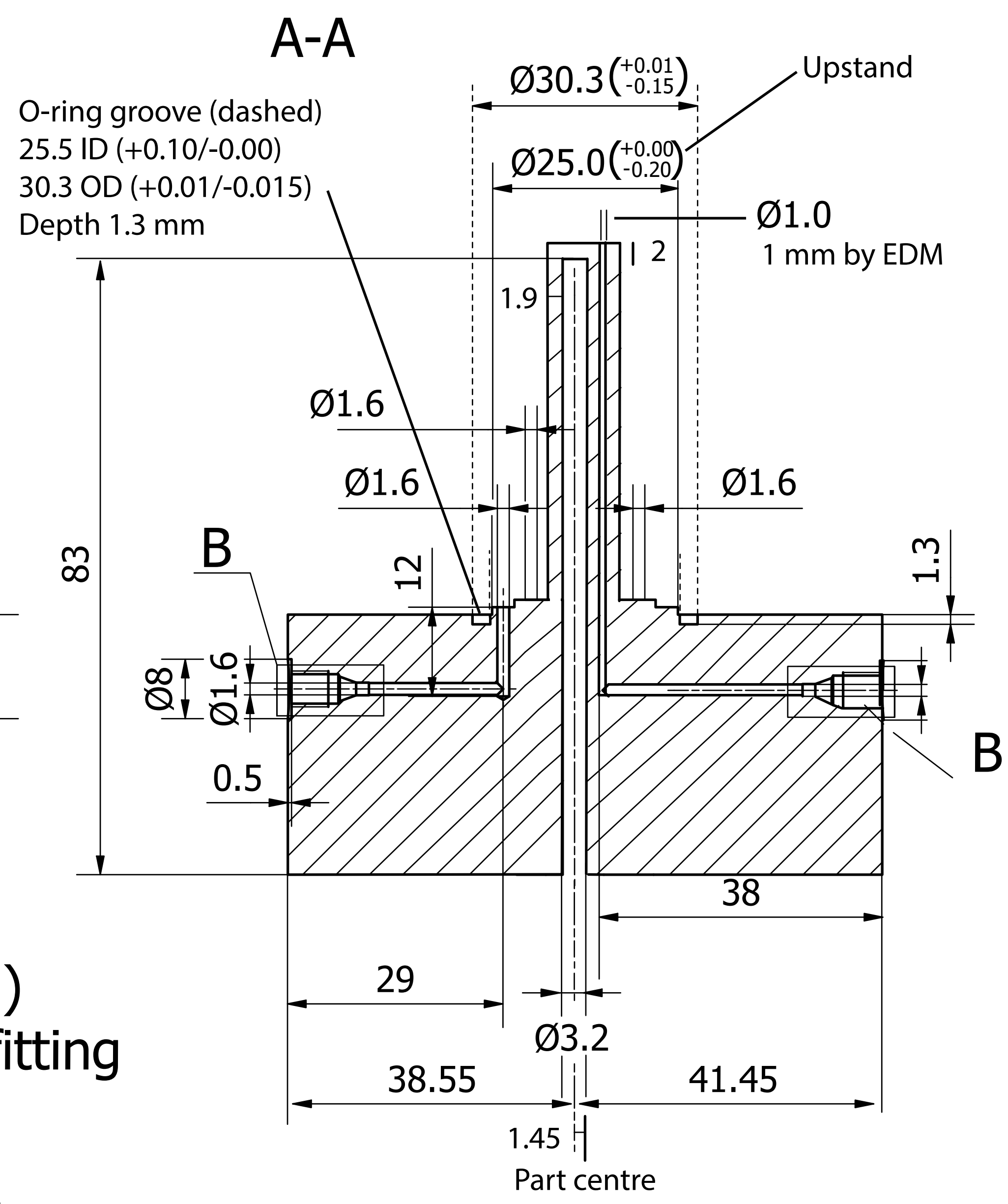

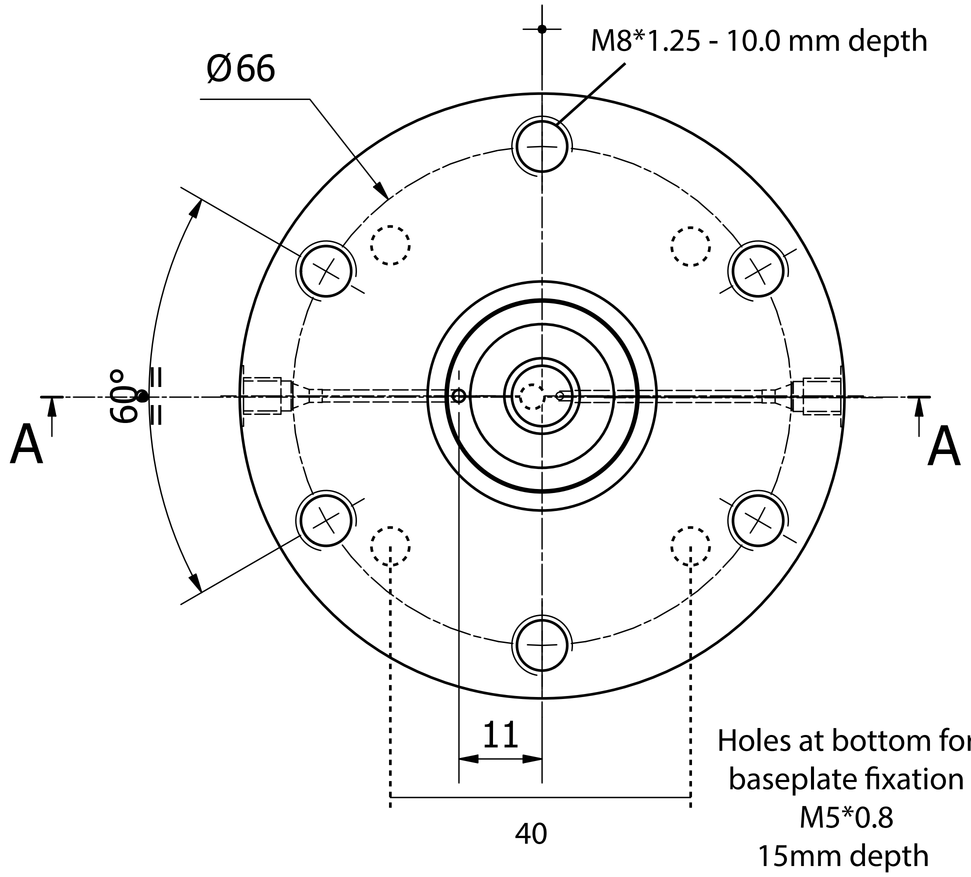

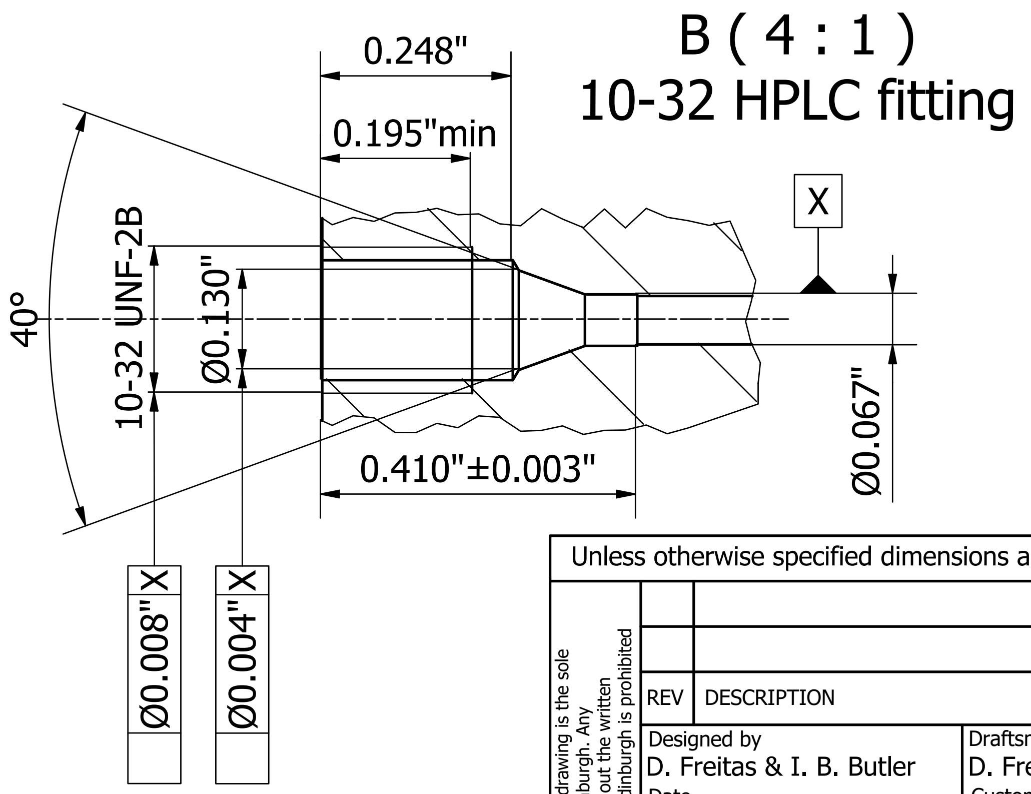

Flow Chart
1) Turn down the external dimensions
2) 3.2 mm hole and side HPLC ending in the upstand
3) Spark erosion for 1 mm hole (external)
4) Drill side HPLC port to merge with 1 mm EDM hole
5) External groves and polishing

Unless otherwise specified dimensions are in millimetres

REV	DESCRIPTION	NAME	DATE

Designed by	Draftsman	Heat treatment / Surface treatment	Format	
D. Freitas & I. B. Butler	D. Freitas		A3	
Date: 31/08/2022	Customer: F. Fusseis		Scale: 1 : 1	

- ÷ XX	XX ÷ XXX	XXX ÷ XXXX	OVER XXXX	Material	Weight	Quantity
±0.1	±0.2	±0.5	±1	Grade 5 Titanium	0.793 kg	

The University Of Edinburgh
School Of GeoSciences

Part code	Edition	Job code
WKSP00228	00	WKS19-045

Part name	Sheet
BOTTOM PISTON / PLATEN	1 / 1

The information contained in this drawing is the sole property of The University Of Edinburgh. Any reproduction in part or whole without the written permission of The University Of Edinburgh is prohibited

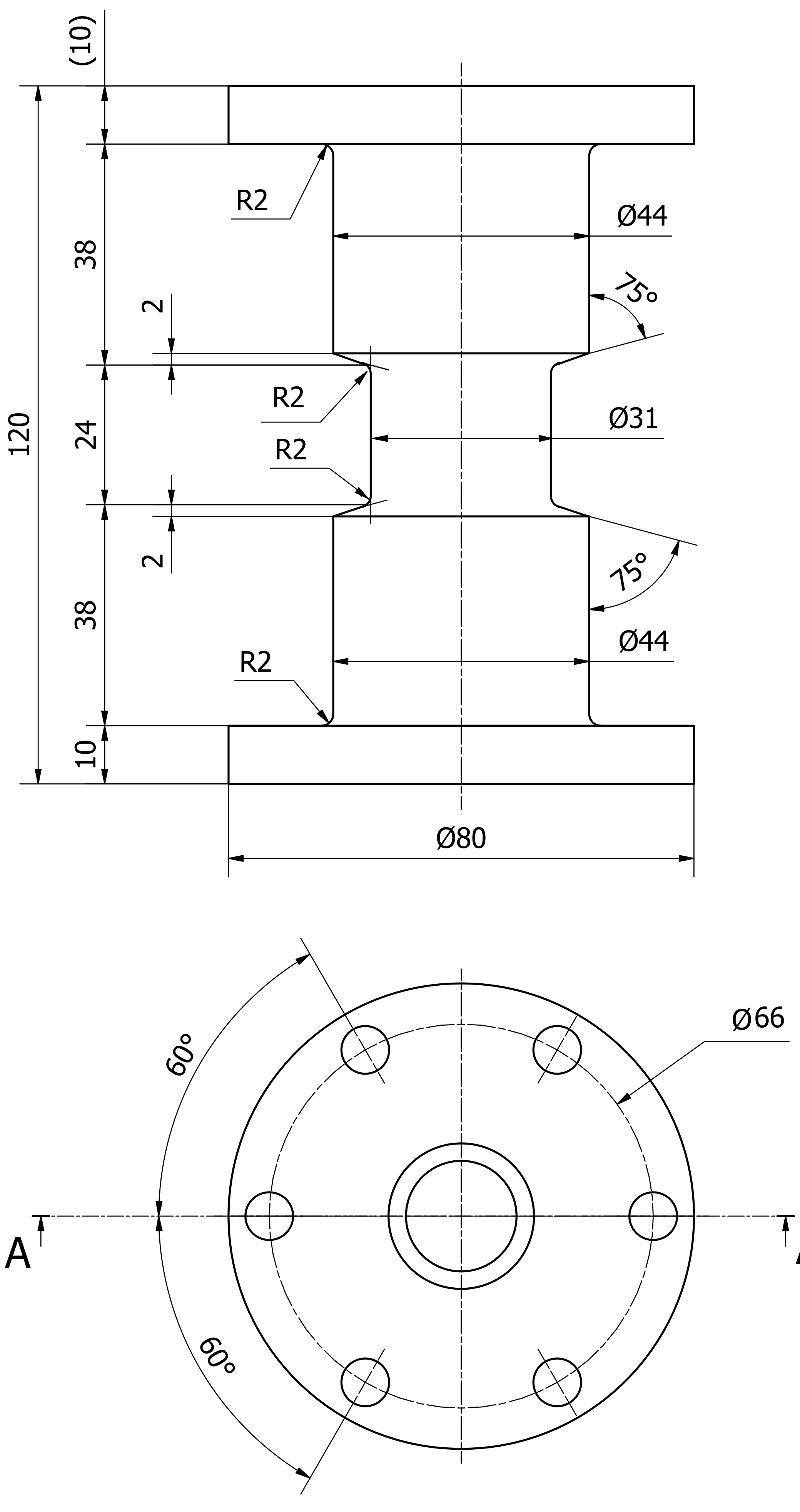

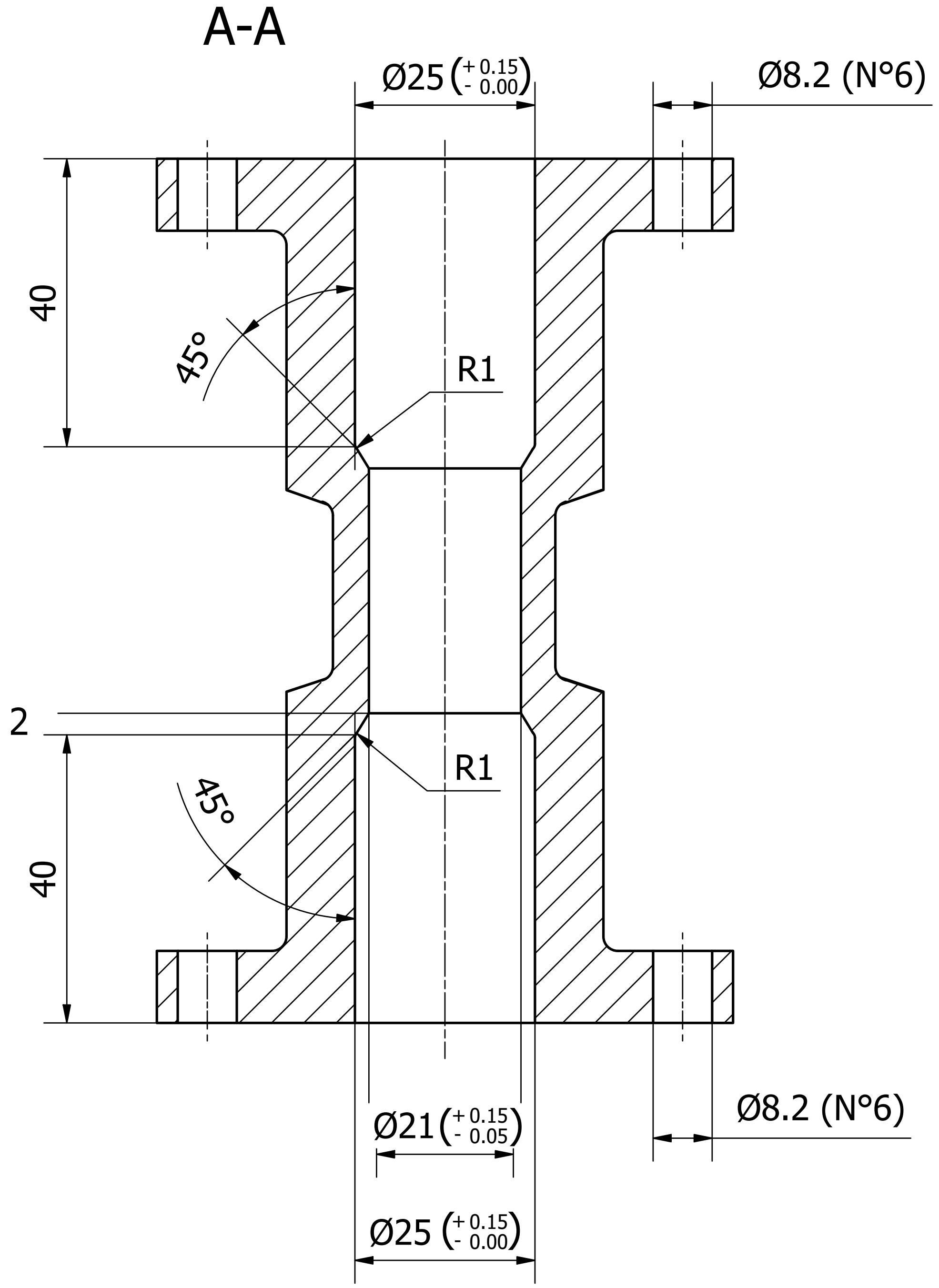

Unless otherwise specified dimensions are in millimetres

REV	DESCRIPTION				NAME	DATE
Designed by D. Freitas & I. B. Butler		Draftsman D. Freitas		Heat treatment / Surface treatment	Format A3	
Date 31/08/2022		Customer F. Fusseis			Scale 1 : 1	
- ÷ XX	XX ÷ XXX	XXX ÷ XXXX	OVER XXXX	Material	Weight	Quantity
±0.1	±0.2	±0.5	±1	Aluminium 7068	0.467 kg	
**The University Of Edinburgh** School Of GeoSciences			Part code WKSP00229	Edition 00	Job code	
			Part name PRESSURE VESSEL			Sheet 1 / 1

The information contained in this drawing is the sole property of The University Of Edinburgh. Any reproduction in part or whole without the written permission of The University Of Edinburgh is prohibited

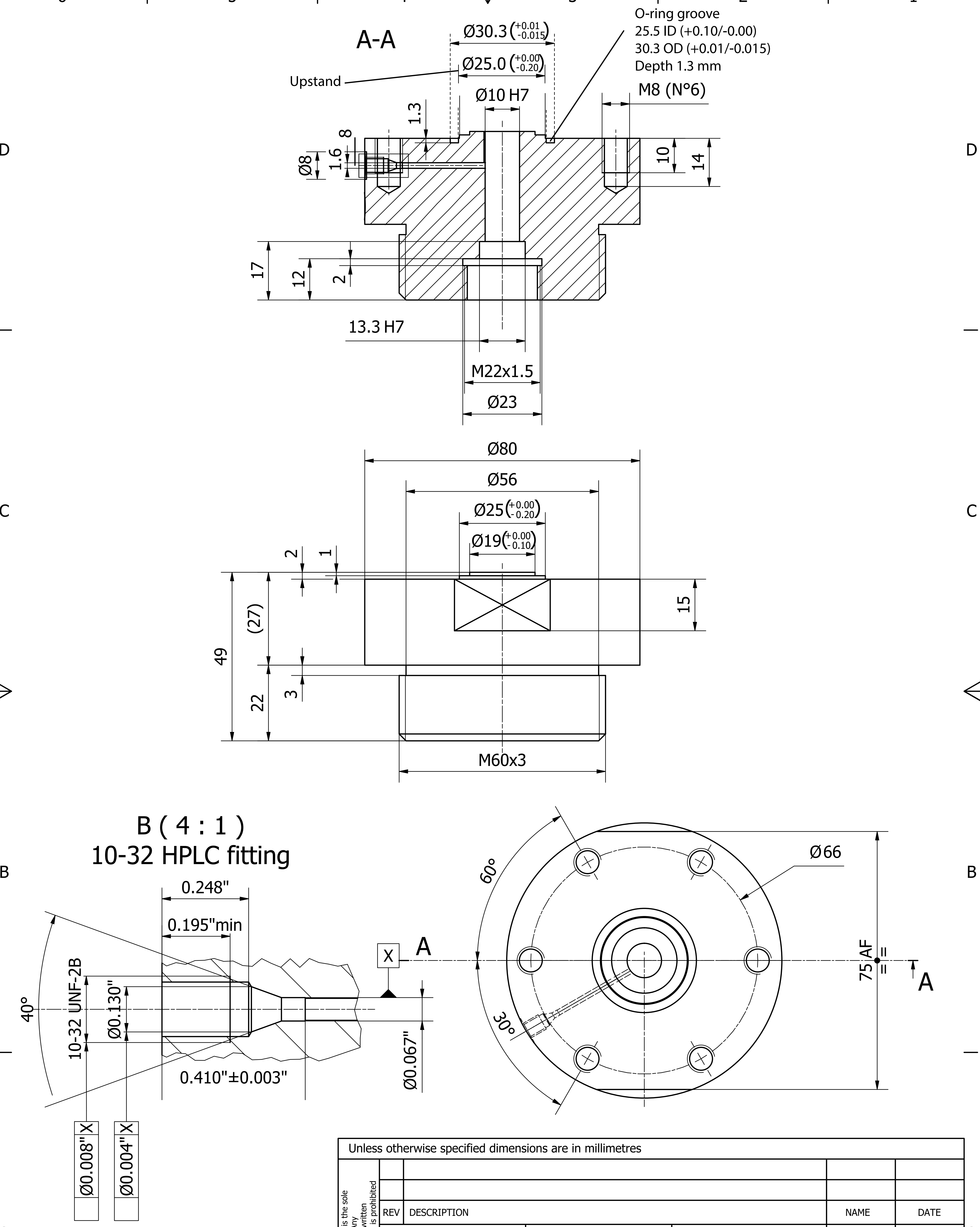

Unless otherwise specified dimensions are in millimetres

REV	DESCRIPTION	NAME	DATE

Designed by	Draftsman	Heat treatment / Surface treatment	Format	
D. Freitas & I. B. Butler	D. Freitas		A3	
Date	Customer		Scale	
31/08/2022	F. Fusseis		1 : 1	

- ÷ XX	XX ÷ XXX	XXX ÷ XXXX	OVER XXXX	Material	Weight	Quantity
±0.1	±0.2	±0.5	±1	Grade 5 Titanium	0.790 kg	

The University Of Edinburgh
School Of GeoSciences

Part code	Edition	Job code
WKSP00232	00	

Part name	Sheet
TOP PLATEN	1 / 1

The information contained in this drawing is the sole property of The University Of Edinburgh. Any reproduction in part or whole without the written permission of The University Of Edinburgh is prohibited

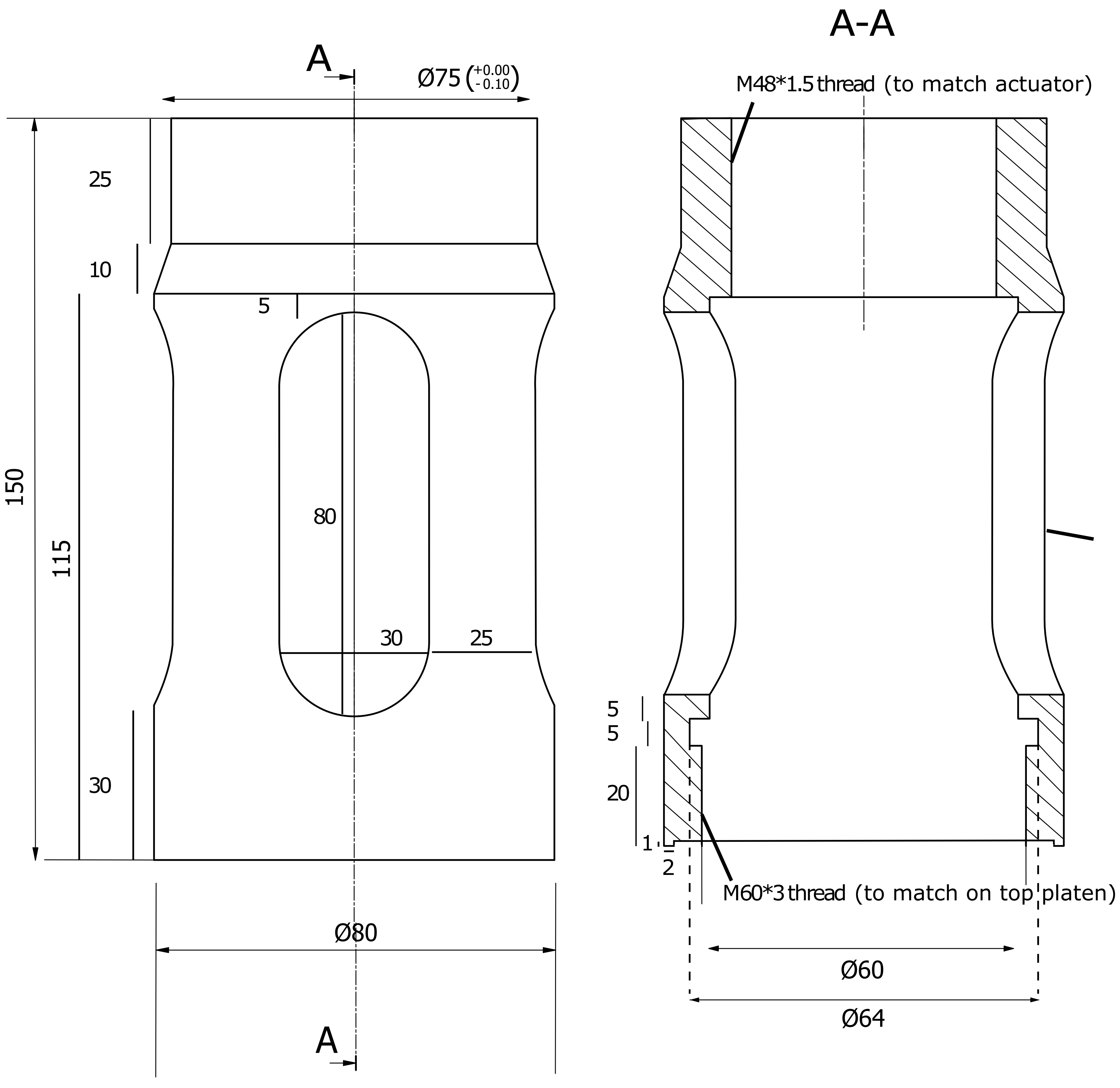

Unless otherwise specified dimensions are in millimetres

The information contained in this drawing is the sole property of The University Of Edinburgh. Any reproduction in part or whole withoutthe written permission of The University Of Edinburgh is prohibited

REV	DESCRIPTION	NAME	DATE
	Grade 5 Titanium		

Designed by<br>D. Freitas & I. B. Butler<br>Date<br>31/08/2022	Draftsman<br>D. Freitas<br>Customer<br>F. Fusseis	Weight<br>3.069 kg<br>Quantity	Job code

Format	Scale	Sheet	Edition
A3	1 : 1	1/1	

The University Of Edinburgh
School Of GeoSciences

Assembly name
Actuator Carrier
Assembly code

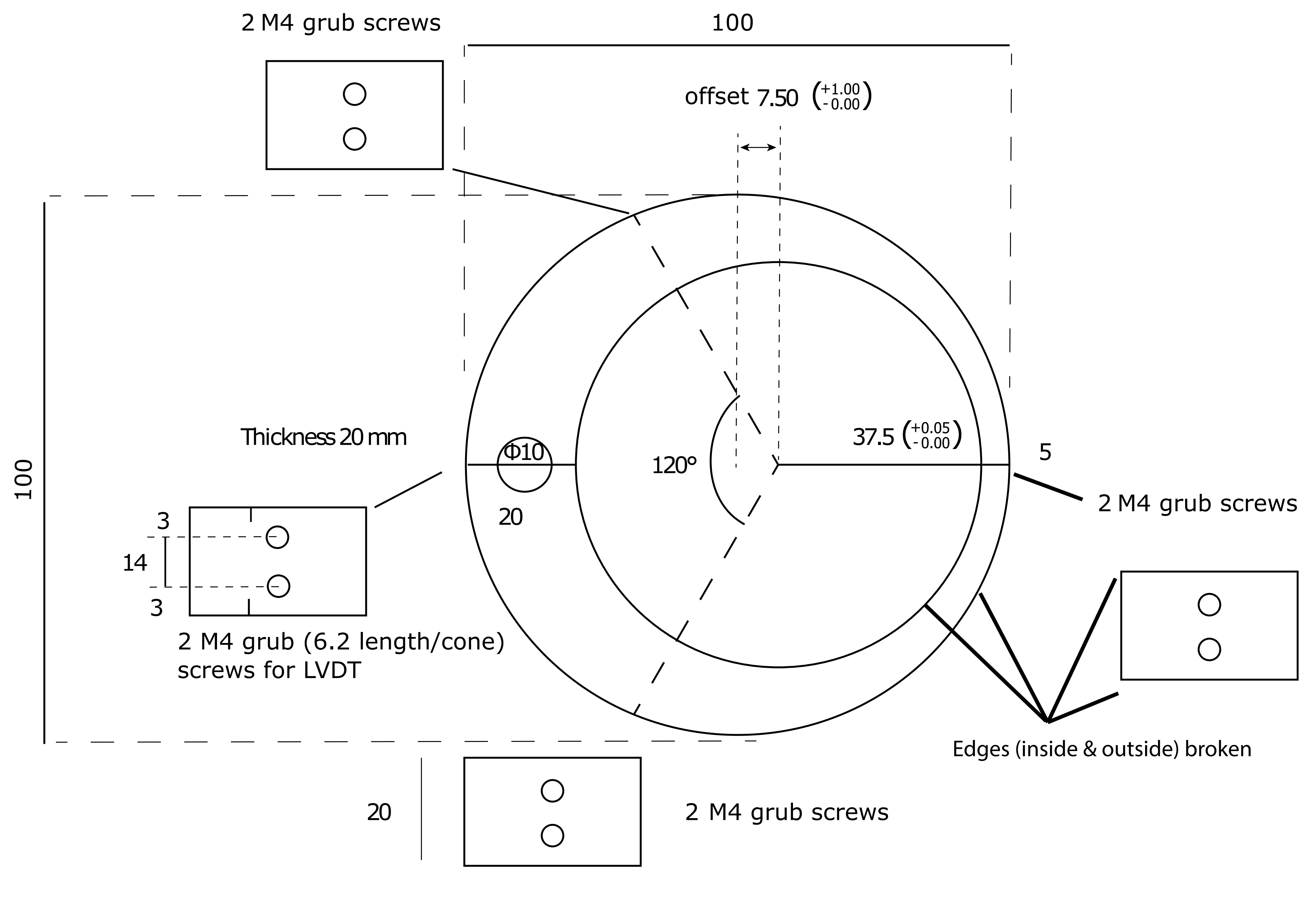

Unless otherwise specified dimensions are in millimetres

	Aluminium 6000 series		
REV	DESCRIPTION	NAME	DATE

Designed by D. Freitas & I. B. Butler<br>Date 31/08/2022	Draftsman D. Freitas<br>Customer F. Fusseis	Weight<br>Quantity	Job code	
**The University Of Edinburgh**<br>School Of GeoSciences		Format A3	Scale 1 : 1	Sheet 1/1	Edition
		Assembly name LVDT ring<br>Assembly code		

The information contained in this drawing is the sole property of The University Of Edinburgh. Any reproduction in part or whole withoutthe written permission of The University Of Edinburgh is prohibited

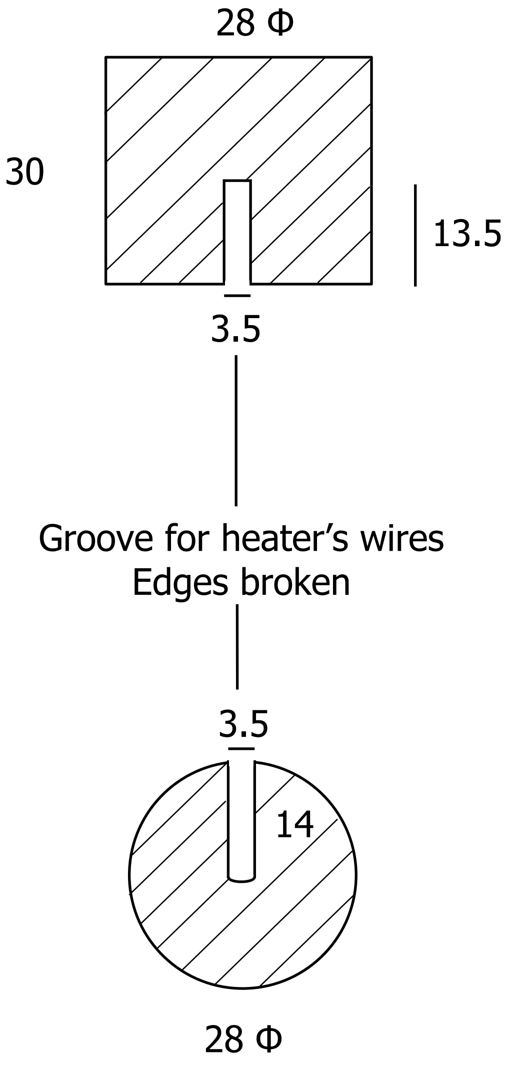

Unless otherwise specified dimensions are in millimetres

REV	DESCRIPTION	NAME	DATE

Designed by<br>D. Freitas & I. B. Butler<br>Date<br>31/08/2022	Draftsman<br>D. Freitas<br>Customer<br>F. Fusseis	Heat treatment / Surface treatment	Format<br>A4<br>Scale<br>1 : 1

- ÷ XX	XX ÷ XXX	XXX ÷ XXXX	OVER XXXX	Material	Weight	Quantity
±0.1	±0.2	±0.5	±1	Stainless Steel	kg	

The University Of Edinburgh
School Of GeoSciences

Part code	Edition	Job code
WKSP00238	00	

Part name	Sheet
SPACER	1 / 1

The information contained in this drawing is the sole property of The University Of Edinburgh. Any reproduction in part or whole without the written permission of The University Of Edinburgh is prohibited

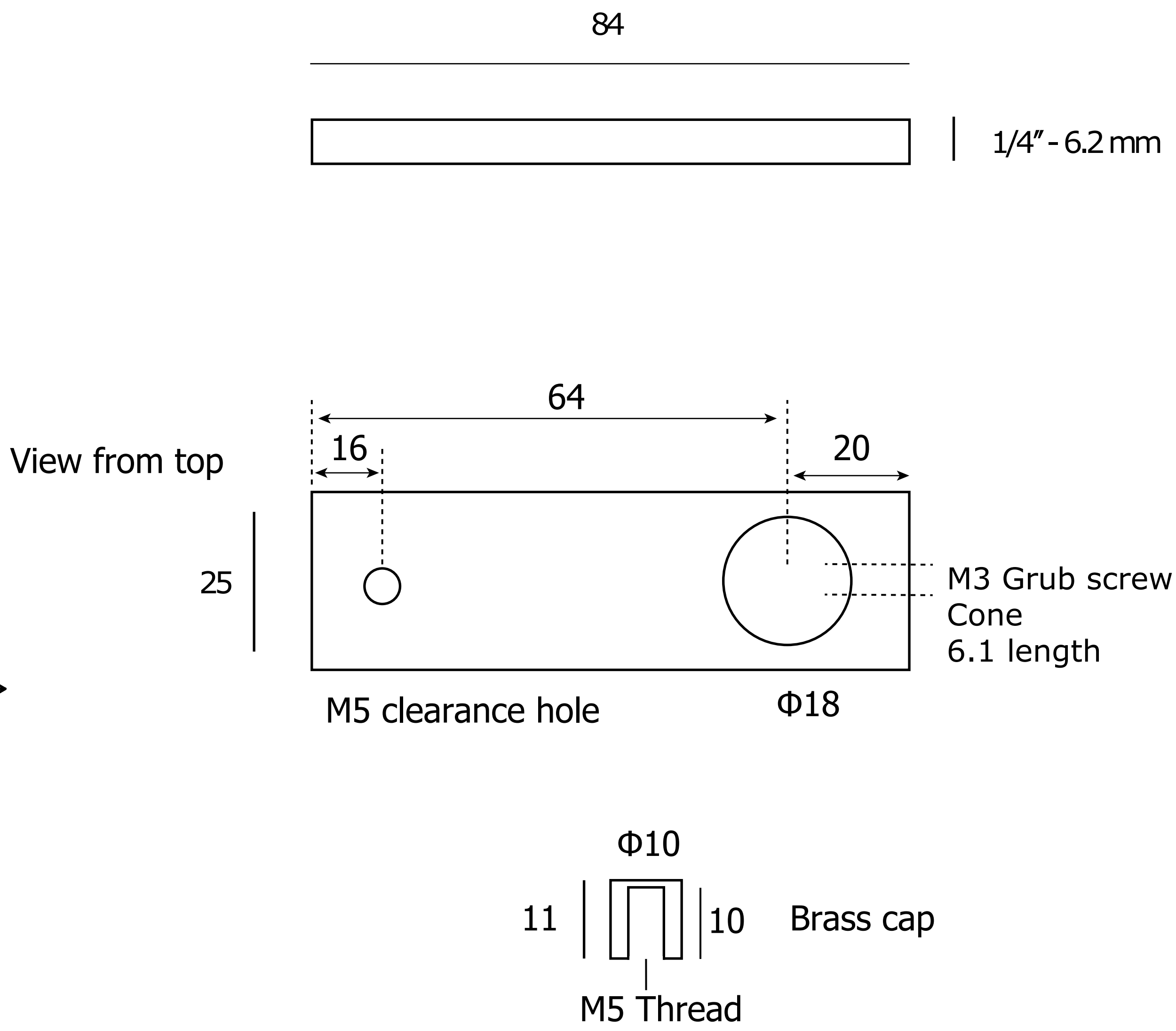

Unless otherwise specified dimensions are in millimetres

REV	DESCRIPTION	NAME	DATE

Designed by D. Freitas & I. B. Butler		Draftsman D. Freitas		Heat treatment / Surface treatment	Format A4	
Date 31/08/2022		Customer F. Fusseis			Scale 1: 1	
- ÷ XX	XX ÷ XXX	XXX ÷ XXXX	OVER XXXX	Material	Weight	Quantity
±0.1	±0.2	±0.5	±1	Stainless Steel		

The University Of Edinburgh
School Of GeoSciences

Part code	Edition	Job code
	00	

Part name	Sheet
LVDT Tell-Tale	1 / 1

The information contained in this drawing is the sole property of The University Of Edinburgh. Any reproduction in part or whole without the written permission of The University Of Edinburgh is prohibited

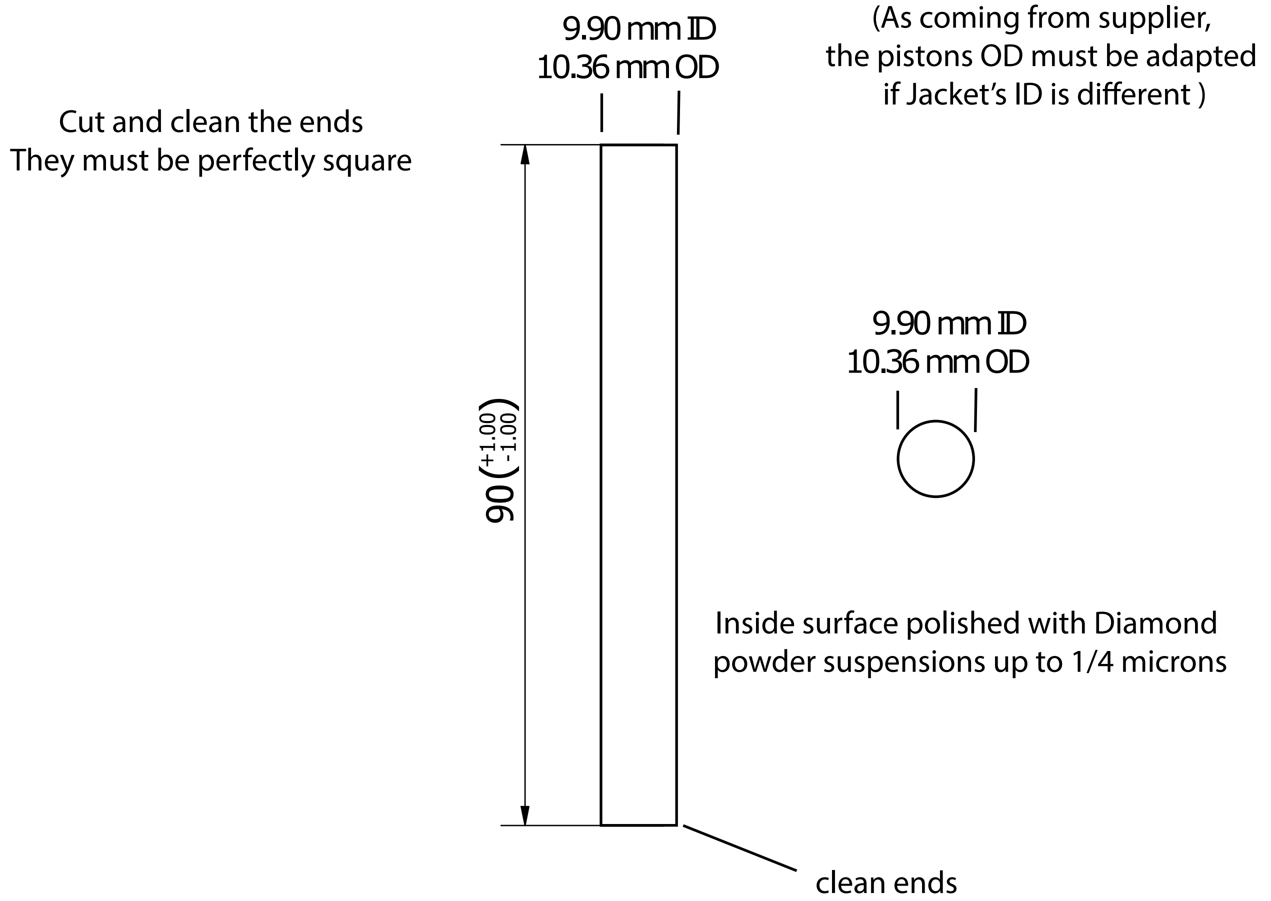

Obtained from Goodfellow copper tubes
(Ref: CU00-TB-000145 Copper)

Unless otherwise specified dimensions are in millimetres

The information contained in this drawing is the sole property of The University Of Edinburgh. Any reproduction in part or whole without the written permission of The University Of Edinburgh is prohibited

REV	DESCRIPTION			NAME	DATE
Designed by<br>D. Freitas & I. B. Butler<br>Date<br>31/08/2022	Draftsman<br>D. Freitas<br>Customer<br>F. Fusseis		Heat treatment / Surface treatment	Format<br>A4<br>Scale<br>1 : 1	
- ÷ XX<br>±0.1	XX ÷ XXX<br>±0.2	XXX ÷ XXXX<br>±0.5	OVER XXXX<br>±1	Material<br>Copper	Weight	Quantity
The University Of Edinburgh<br>School Of GeoSciences		Part code	Edition<br>00	Job code	
		Part name<br>METAL JACKET			Sheet<br>1 / 1

Graphite sleeve

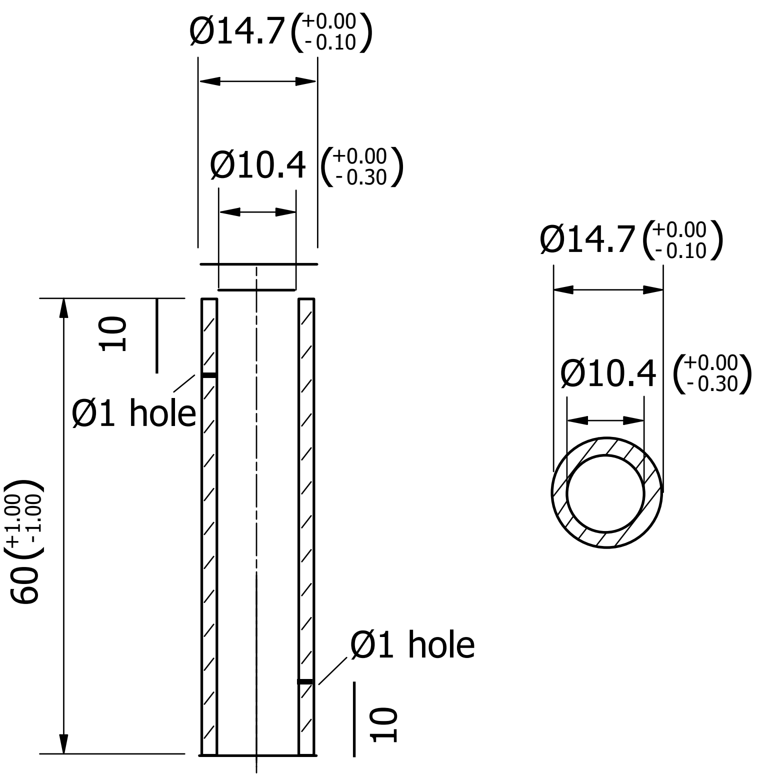

+2 Manual shavers/hand reamers to adjust ID perfectly to the Copper Jacket

Unless otherwise specified dimensions are in millimetres

REV	DESCRIPTION	NAME	DATE

Designed by<br>D. Freitas & I. B. Butler<br>Date<br>31/08/2022		Draftsman<br>D. Freitas<br>Customer<br>F. Fusseis		Heat treatment / Surface treatment	Format<br>A4<br>Scale<br>1 : 1	
- ÷ XX	XX ÷ XXX	XXX ÷ XXXX	OVER XXXX	Material<br>Graphite 6507	Weight	Quantity
±0.1	±0.2	±0.5	±1			

The University Of Edinburgh
School Of GeoSciences

Part code	Edition<br>00	Job code
Part name<br>GRAPHITE SLEEVE		Sheet<br>1 / 1

The information contained in this drawing is the sole property of The University Of Edinburgh. Any reproduction in part or whole without the written permission of The University Of Edinburgh is prohibited

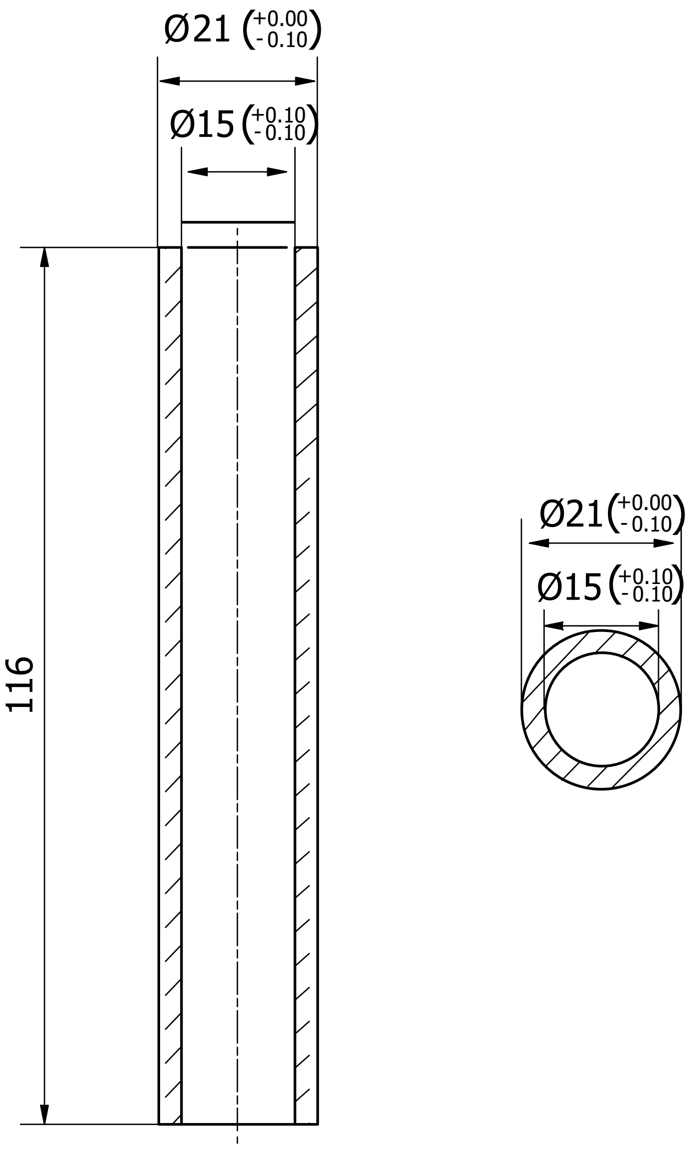

Unless otherwise specified dimensions are in millimetres

REV	DESCRIPTION	NAME	DATE

Designed by	Draftsman	Heat treatment / Surface treatment	Format	
D. Freitas & I. B. Butler	D. Freitas		A4	
Date	Customer		Scale	
31/08/2022	F. Fusseis		1 : 1	

- ÷ XX	XX ÷ XXX	XXX ÷ XXXX	OVER XXXX	Material	Weight	Quantity
±0.1	±0.2	±0.5	±1	PEEK		

The University Of Edinburgh
School Of GeoSciences

Part code	Edition	Job code
WKSP00230	00	

Part name	Sheet
THERMAL INSULATION BAFFLE	1 / 1

The information contained in this drawing is the sole property of The University Of Edinburgh. Any reproduction in part or whole without the written permission of The University Of Edinburgh is prohibited

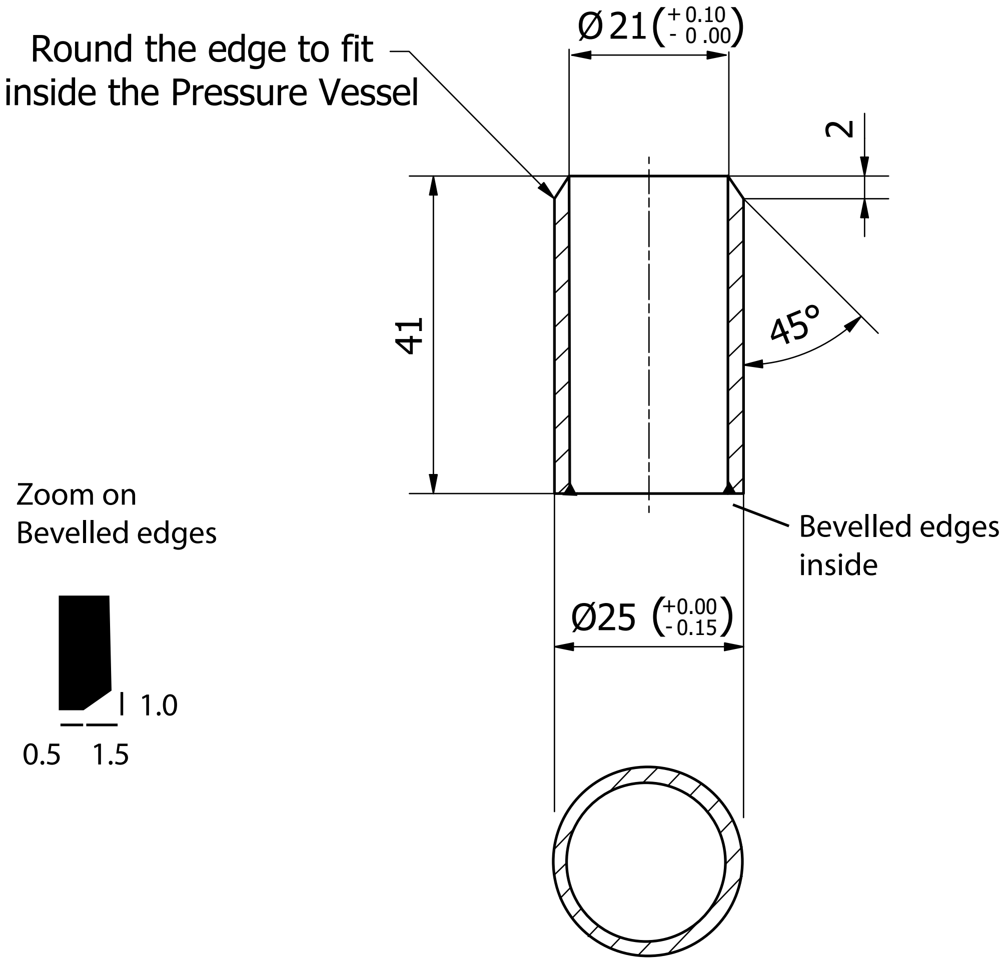

Unless otherwise specified dimensions are in millimetres

REV	DESCRIPTION	NAME	DATE

Designed by	Draftsman	Heat treatment / Surface treatment	Format
D. Freitas & I. B. Butler	D. Freitas		A4
Date: 31/08/2022	Customer: F. Fusseis		Scale: 1 : 1

- ÷ XX	XX ÷ XXX	XXX ÷ XXXX	OVER XXXX	Material	Weight	Quantity
±0.1	±0.2	±0.5	±1	PEEK	0.011 kg	

The University Of Edinburgh
School Of GeoSciences

Part code	Edition	Job code
WKSP00231	00	

Part name	Sheet
EXTERNAL BAFFLE	1 / 1

The information contained in this drawing is the sole property of The University Of Edinburgh. Any reproduction in part or whole without the written permission of The University Of Edinburgh is prohibited

Flow Chart
1) Taper 10 mm to 9.90 mm (1)
2) Cut the piston to lenght = 130 mm (1)
3) Gundrilling for 3.2 mm hole (external)
4) Spark erosion for 1 mm hole (external)
5) check where the 1 mm hole ends
and drill 2 mm hole to connect on the side (2)
6) Adjust the length of the piston to have the
2 mm hole at about 8.3 mm from top (2)
7) Taper on the top end of the piston to 9.5 mm
+ broken edges (2)
8) Radius on the 3.2 mm hole
+ unsharpenned edges (2)
9) Circumferencial grooves (3)

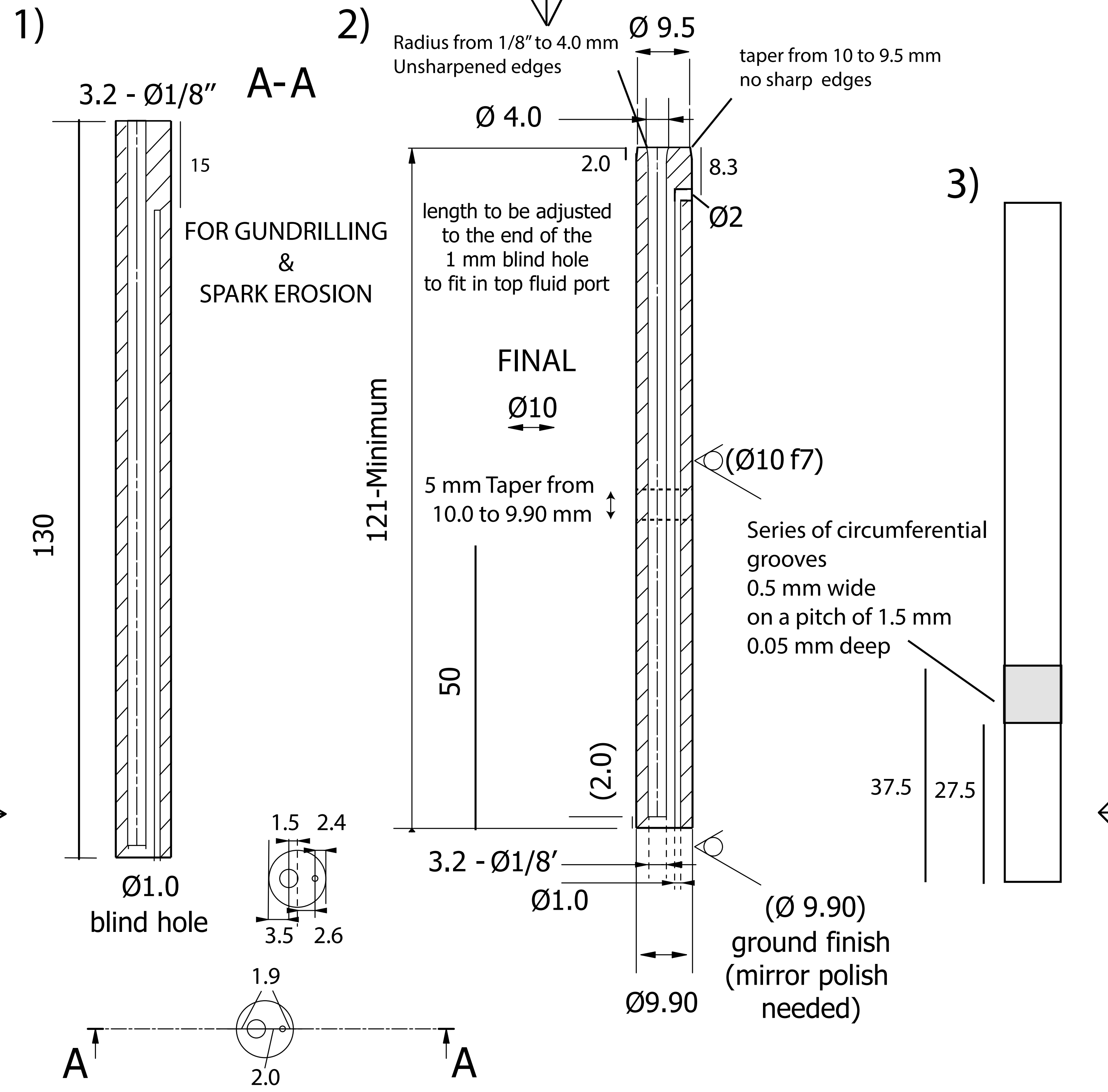

To be obtained from a Ø12 f7 centerless ground bar

Unless otherwise specified dimensions are in millimetres

REV	DESCRIPTION	NAME	DATE

Designed by D. Freitas & I. B. Butler	Draftsman D. Freitas	Heat treatment / Surface treatment	Format A4
Date 31/08/2022	Customer F. Fusseis		Scale 1: 1

- ÷ XX	XX ÷ XXX	XXX ÷ XXXX	OVER XXXX	Material	Weight	Quantity
±0.1	±0.2	±0.5	±1	Grade 5 Titanium	0.065 kg	

The University Of Edinburgh
School Of GeoSciences

Part code	Edition	Job code
WKSP00233	00	

Part name	Sheet
TOP PISTON	1 / 1

The information contained in this drawing is the sole property of The University Of Edinburgh. Any reproduction in part or whole without the written permission of The University Of Edinburgh is prohibited

Made to fit Top Platen
each top platen has its own retainer

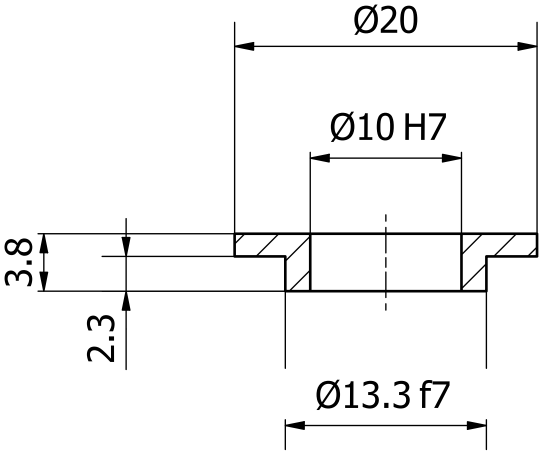

Unless otherwise specified dimensions are in millimetres

REV	DESCRIPTION			NAME	DATE

Designed by D. Freitas & I. B. Butler	Draftsman D. Freitas	Heat treatment / Surface treatment	Format A4	
Date 31/08/2022	Customer F. Fusseis		Scale 1 : 1	

- ÷ XX	XX ÷ XXX	XXX ÷ XXXX	OVER XXXX	Material	Weight	Quantity
±0.1	±0.2	±0.5	±1	Phosphor Bronze	0.002 kg	

The University Of Edinburgh
School Of GeoSciences

Part code	Edition	Job code
	00	

Part name	Sheet
SEAL RETAINER	1 / 1

The information contained in this drawing is the sole property of The University Of Edinburgh. Any reproduction in part or whole without the written permission of The University Of Edinburgh is prohibited

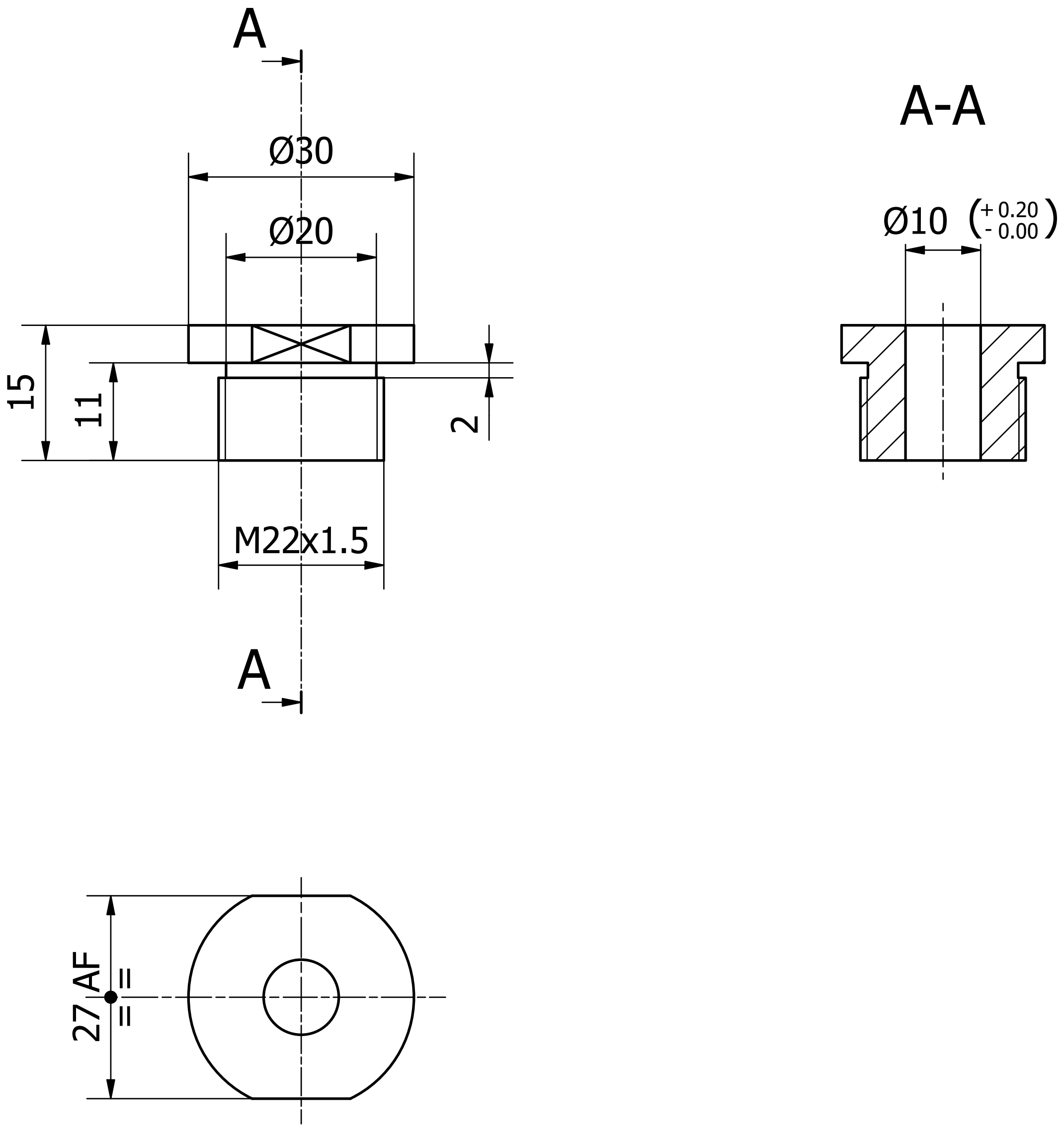

Unless otherwise specified dimensions are in millimetres

REV	DESCRIPTION	NAME	DATE

Designed by: D. Freitas & I. B. Butler
Date: 31/08/2022
Draftsman: D. Freitas
Customer: F. Fusseis
Heat treatment / Surface treatment
Format: A4
Scale: 1 : 1

- ÷ XX	XX ÷ XXX	XXX ÷ XXXX	OVER XXXX
±0.1	±0.2	±0.5	±1

Material: Grade 5 Titanium
Weight: 0.031 kg
Quantity

The University Of Edinburgh
School Of GeoSciences

Part code: WKSP00234
Edition: 00
Job code
Part name: Seal retainer nut
Sheet: 1 / 1

The information contained in this drawing is the sole property of The University Of Edinburgh. Any reproduction in part or whole without the written permission of The University Of Edinburgh is prohibited

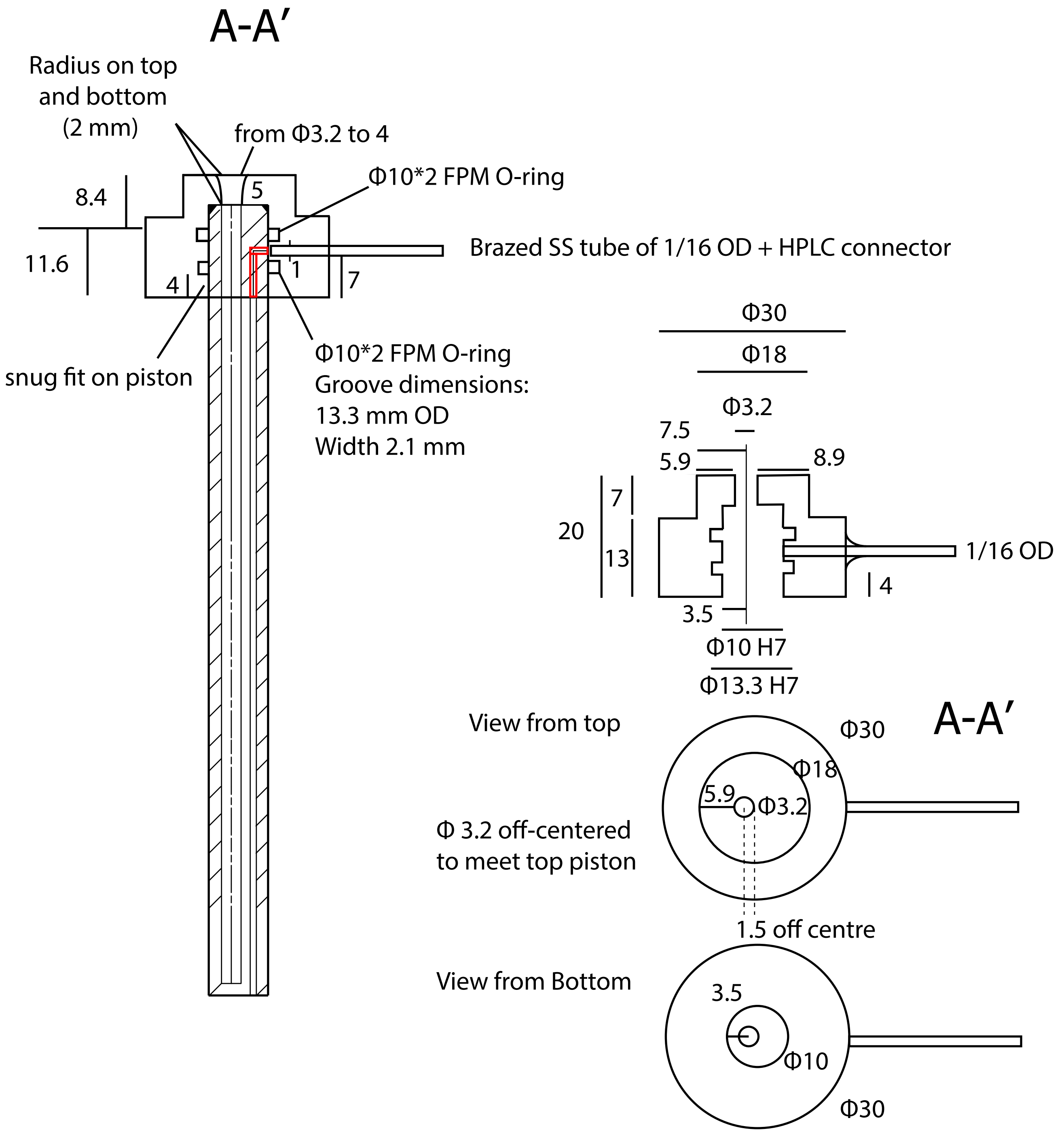

Unless otherwise specified dimensions are in millimetres

REV	DESCRIPTION	NAME	DATE

Designed by D. Freitas & I. B. Butler		Draftsman D. Freitas		Heat treatment / Surface treatment	Format A4	
Date 31/08/2022		Customer F. Fusseis			Scale 1: 1	
- ÷ XX	XX ÷ XXX	XXX ÷ XXXX	OVER XXXX	Material	Weight	Quantity
±0.1	±0.2	±0.5	±1	Stainless steel	0.065 kg	

The University Of Edinburgh
School Of GeoSciences

Part code	Edition 00	Job code
Part name: Top fluid port		Sheet 1 / 1

The information contained in this drawing is the sole property of The University Of Edinburgh. Any reproduction in part or whole without the written permission of The University Of Edinburgh is prohibited

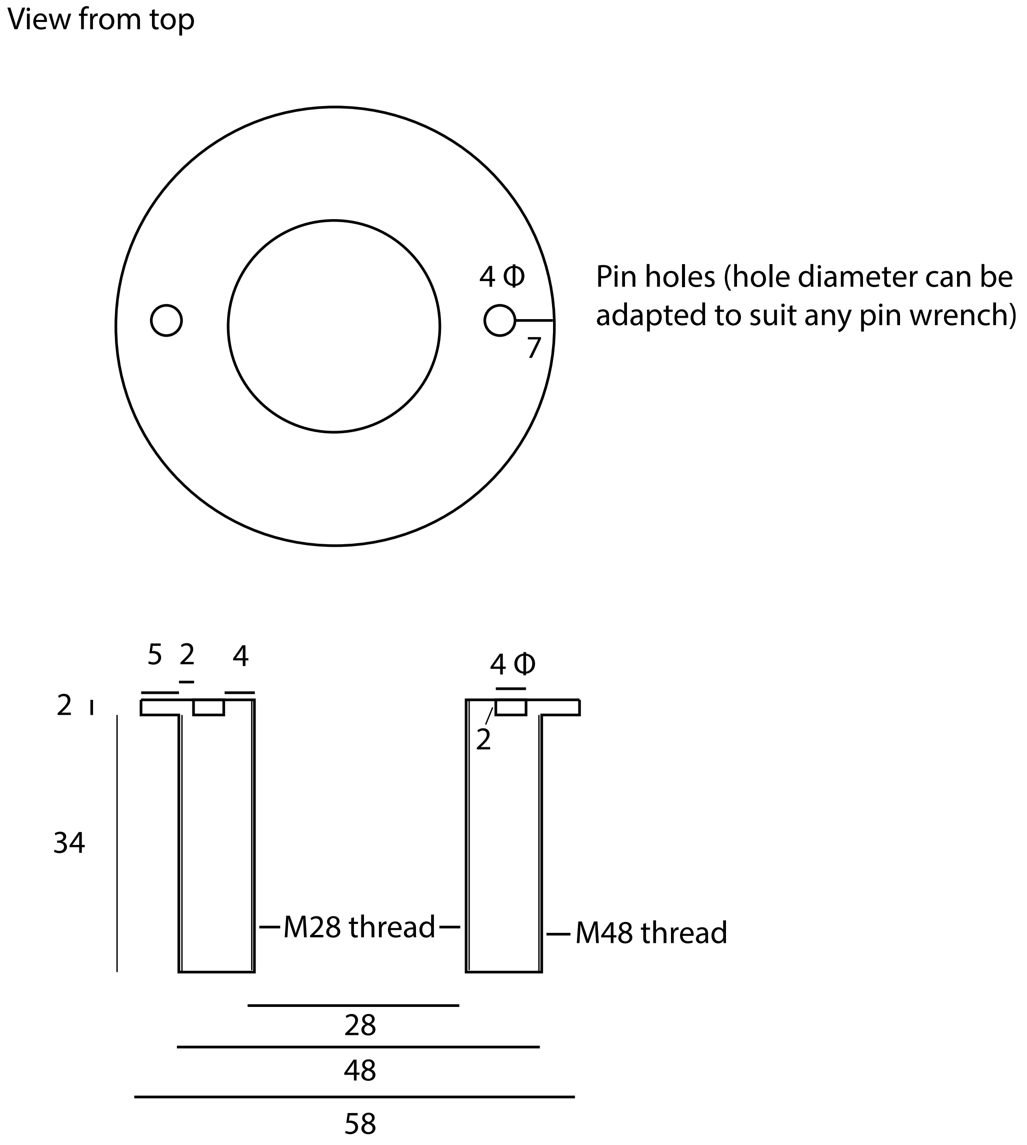

Unless otherwise specified dimensions are in millimetres

The information contained in this drawing is the sole property of The University Of Edinburgh. Any reproduction in part or whole without the written permission of The University Of Edinburgh is prohibited

REV	DESCRIPTION	NAME	DATE

Designed by<br>D. Freitas & I. B. Butler<br>Date<br>31/08/2022		Draftsman<br>D. Freitas<br>Customer<br>F. Fusseis		Heat treatment / Surface treatment	Format<br>A4<br>Scale<br>1: 1
- ÷ XX	XX ÷ XXX	XXX ÷ XXXX	OVER XXXX	Material	Weight	Quantity
±0.1	±0.2	±0.5	±1	Stainless steel		

The University Of Edinburgh
School Of GeoSciences

Part code	Edition	Job code
	00	

Part name	Sheet
Actuator adaptator	1 / 1

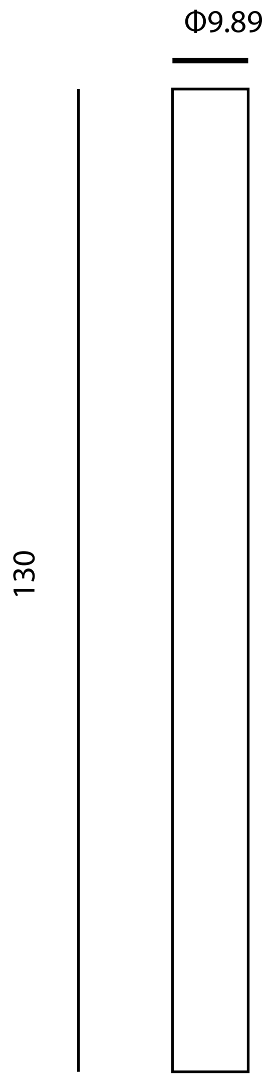

Slightly smaller than pistons
Mirror polished
Used to gage the jackets
& assemble

Unless otherwise specified dimensions are in millimetres

REV	DESCRIPTION	NAME	DATE

Designed by D. Freitas & I. B. Butler	Draftsman D. Freitas	Heat treatment / Surface treatment	Format A4
Date 31/08/2022	Customer F. Fusseis		Scale 1 : 1

- ÷ XX	XX ÷ XXX	XXX ÷ XXXX	OVER XXXX	Material	Weight	Quantity
±0.1	±0.2	±0.5	±1	Stainless Steel	kg	

The University Of Edinburgh
School Of GeoSciences

Part code	Edition	Job code
WKSP00235	00	

Part name: Piston Guide
Sheet 1 / 1

The information contained in this drawing is the sole property of The University Of Edinburgh. Any reproduction in part or whole without the written permission of The University Of Edinburgh is prohibited

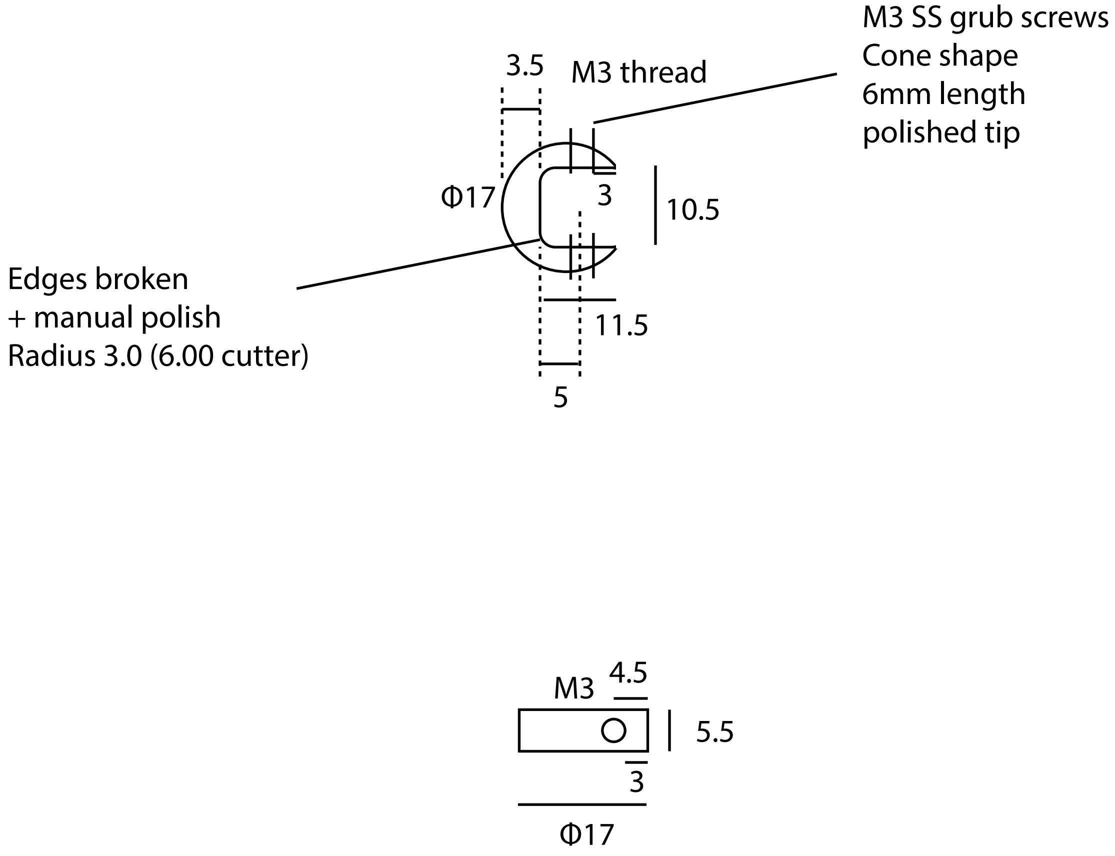

Unless otherwise specified dimensions are in millimetres

REV	DESCRIPTION	NAME	DATE

Designed by<br>D. Freitas & I. B. Butler<br>Date<br>31/08/2022	Draftsman<br>D. Freitas<br>Customer<br>F. Fusseis	Heat treatment / Surface treatment	Format<br>A4<br>Scale<br>1 : 1

- ÷ XX	XX ÷ XXX	XXX ÷ XXXX	OVER XXXX	Material	Weight	Quantity
±0.1	±0.2	±0.5	±1	Stainless Steel	0.002 kg	

The University Of Edinburgh
School Of GeoSciences

Part code	Edition	Job code
WKSP00235	00	

Part name	Sheet
Ring Installer	1 / 1

The information contained in this drawing is the sole property of The University Of Edinburgh. Any reproduction in part or whole without the written permission of The University Of Edinburgh is prohibited

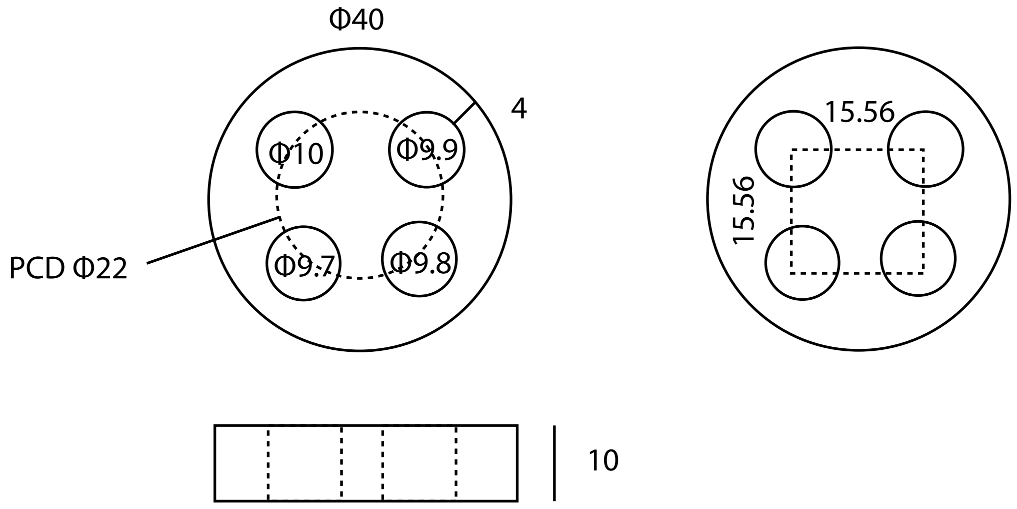

Unless otherwise specified dimensions are in millimetres

REV	DESCRIPTION	NAME	DATE
	Stainless steel		

Designed by<br>D. Freitas & I. B. Butler<br>Date<br>31/08/2022	Draftsman<br>D. Freitas<br>Customer<br>F. Fusseis	Weight<br>Quantity	Job code
		Format<br>A3	Scale<br>1 : 1
		Sheet<br>1/1	Edition

The University Of Edinburgh
School Of GeoSciences

Assembly name
Sample polishing guide
Assembly code

The information contained in this drawing is the sole property of The University Of Edinburgh. Any reproduction in part or whole withoutthe written permission of The University Of Edinburgh is prohibited

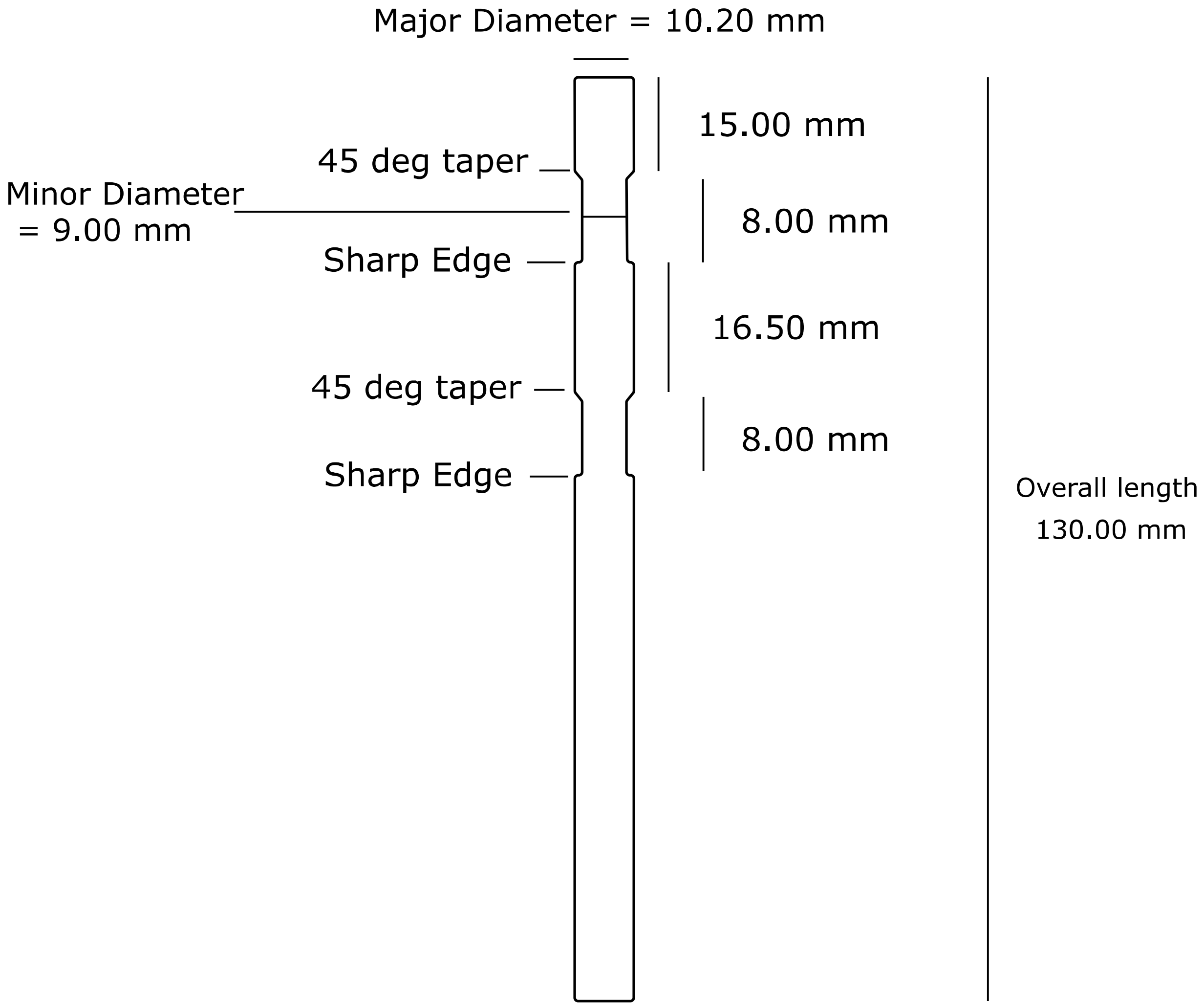

Not to scale

Unless otherwise specified dimensions are in millimetres

REV	DESCRIPTION	NAME	DATE

Designed by	Draftsman	Heat treatment / Surface treatment	Format	
D. Freitas & I. B. Butler	D. Freitas		A4	
Date	Customer		Scale	
27/07/2023	F. Fusseis		1 : 1	

- ÷ XX	XX ÷ XXX	XXX ÷ XXXX	OVER XXXX	Material	Weight	Quantity
±0.1	±0.2	±0.5	±1	Stainless steel		

The University Of Edinburgh
School Of GeoSciences

Part code	Edition	Job code
	00	

Part name	Sheet
GRAPHITE SHAVER	1 / 1

The information contained in this drawing is the sole property of The University Of Edinburgh. Any reproduction in part or whole without the written permission of The University Of Edinburgh is prohibited

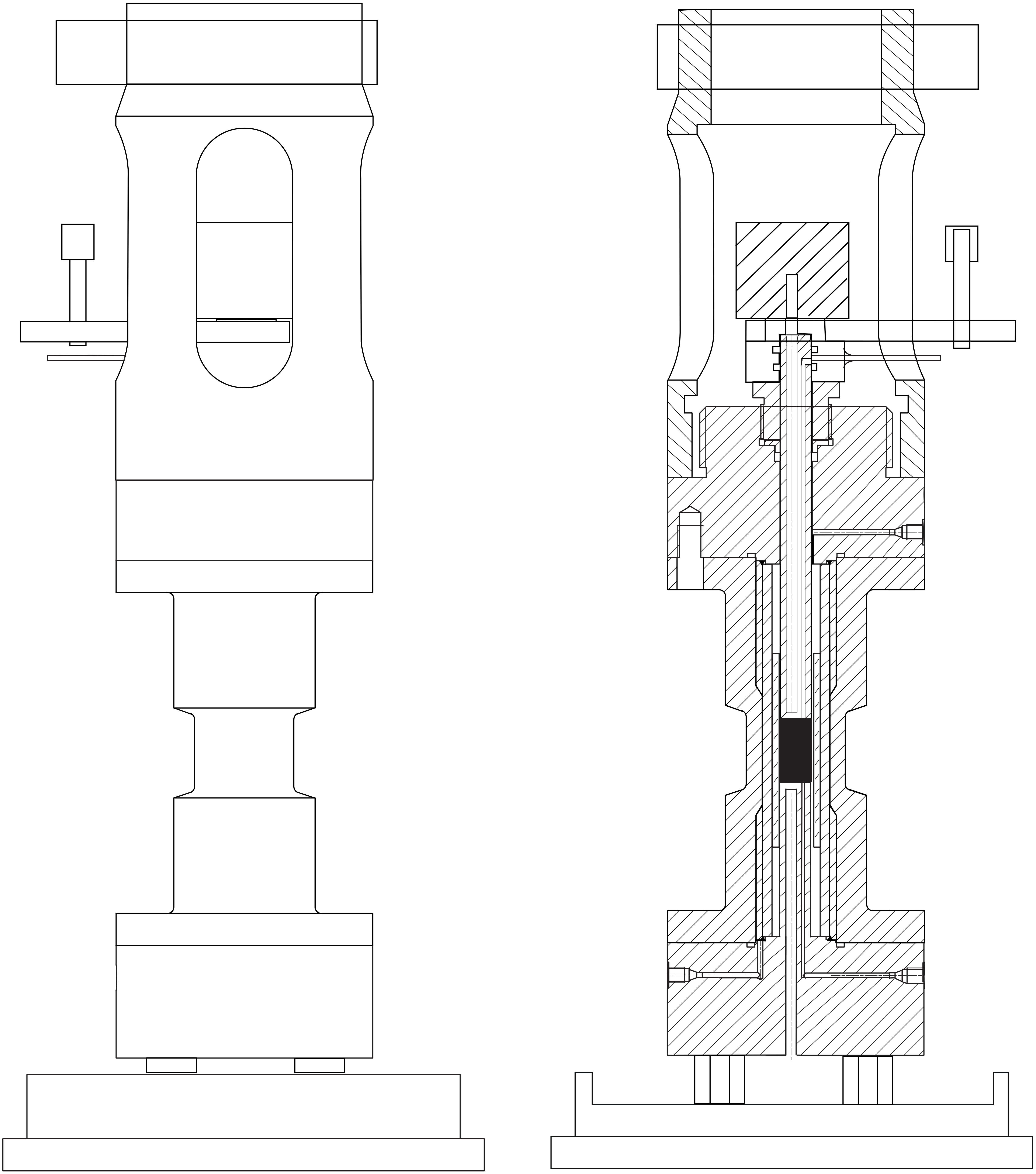

Supplement: Supplementary file 3 [file s-31-00150-sup3.pdf]
