# Supplementary material for: Heitt Mjölnir: a heated miniature triaxial apparatus for 4D synchrotron microtomography
Source: J Synchrotron Radiat. 2024 Jan 1;31(Pt 1):150–61. doi: 10.1107/S1600577523009876 (PMC10833432; doi:10.1107/S1600577523009876)
Supplement: Supplementary file 4 [file s-31-00150-sup4.zip › HM_3D_CAD_drawings/Actuator Piston Cap.pdf]

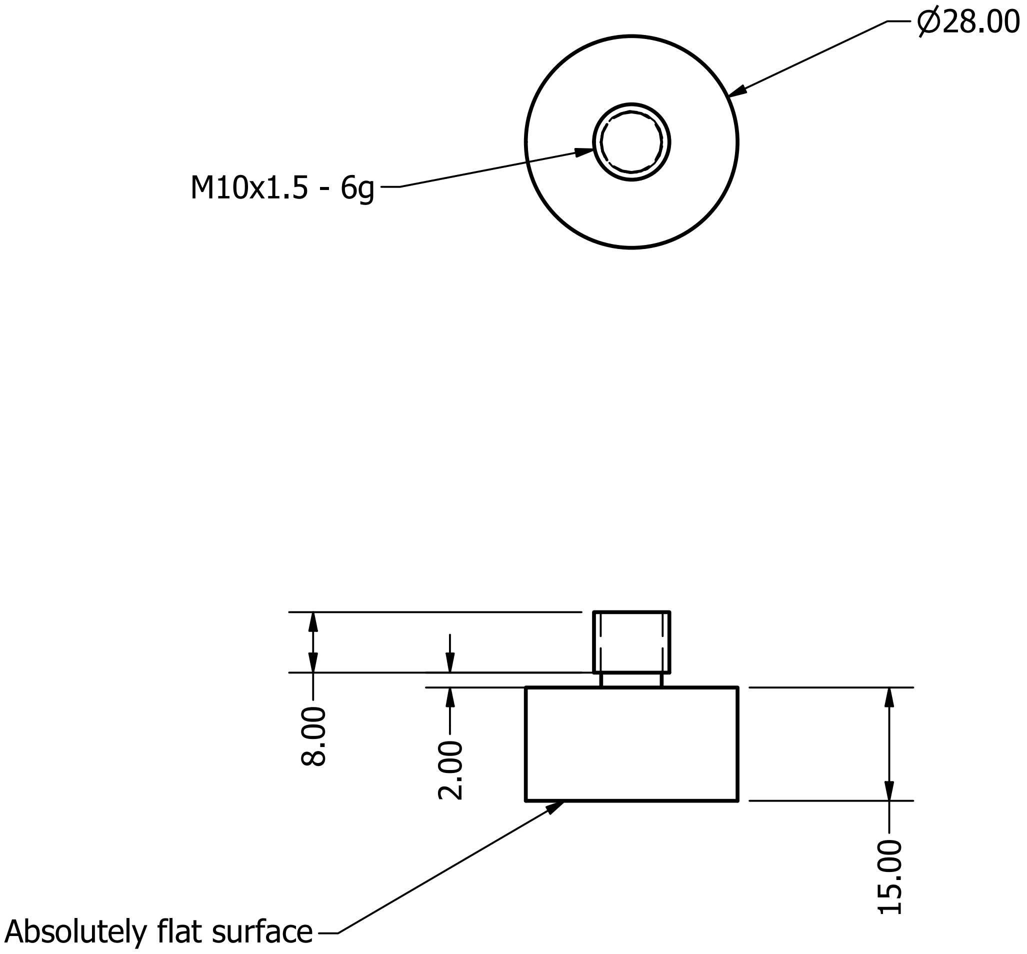

Unless otherwise specified dimensions are in millimeters

Description

Designed by Damien Freitas/Ian Butler<br>Date 21-09-2023		Draftsman Chris McCartney<br>Customer Damien Freitas		Heat Treatment/ Surface Treatment	Format A4<br>Scale 1:1	
- ÷ XX	XX ÷ XXX	XXX ÷ XXXX	OVER XXXX	Material<br>Stainless Steel	Weight	Quantity
±0.1	±0.2	±0.5	±1			

The University of Edinburgh
School of Geosciences

Part Code
Job Code
Part Name Actuator Piston Cap
Sheet

The information contained in this drawing is the sole property of The University Of Edinburgh. Any reproduction in part or whole without written permission of The University of Edinburgh is prohibited
