# Supplementary material for: Heitt Mjölnir: a heated miniature triaxial apparatus for 4D synchrotron microtomography
Source: J Synchrotron Radiat. 2024 Jan 1;31(Pt 1):150–61. doi: 10.1107/S1600577523009876 (PMC10833432; doi:10.1107/S1600577523009876)
Supplement: Supplementary file 4 [file s-31-00150-sup4.zip › HM_3D_CAD_drawings/Baseplate.pdf]

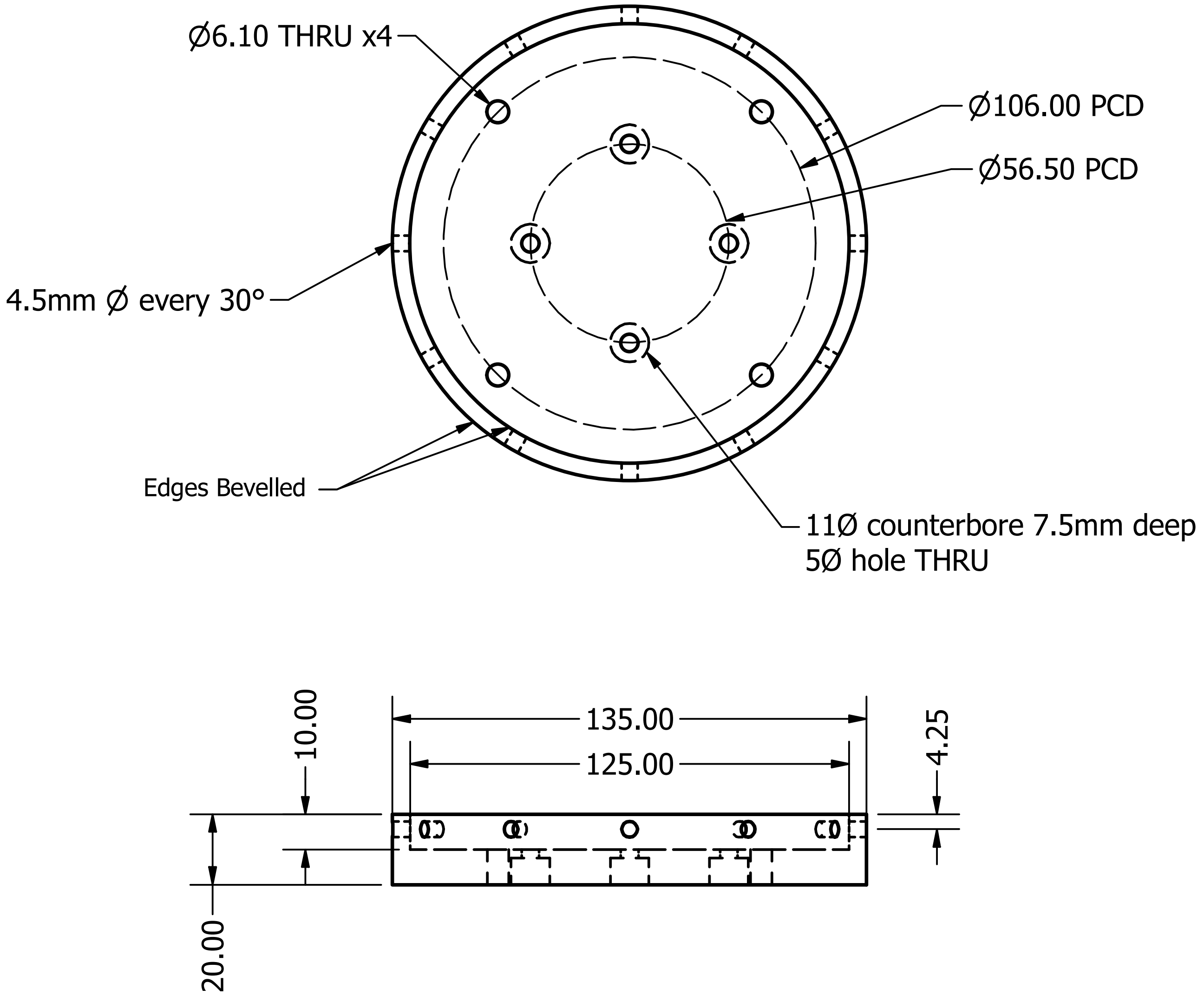

Unless otherwise specified dimensions are in millimeters

Description

Designed by<br>Damien Freitas/Ian Butler<br>Date<br>21-09-2023		Draftsman<br>Chris McCartney<br>Customer<br>Damien Freitas	Heat Treatment/<br>Surface Treatment	Format<br>A4<br>Scale<br>1:1	
- ÷ XX	XX ÷ XXX	XXX ÷ XXXX	OVER XXXX		
±0.1	±0.2	±0.5	±1		
			Material<br>Aluminium 6000 Series	Weight	Quantity
			Part Code		Job Code
			Part Name<br>Baseplate		Sheet

The University of Edinburgh
School of Geosciences

The information contained in this drawing is the sole property of The University Of Edinburgh. Any reproduction in part or whole without written permission of The University of Edinburgh is prohibited
