# Supplementary material for: Heitt Mjölnir: a heated miniature triaxial apparatus for 4D synchrotron microtomography
Source: J Synchrotron Radiat. 2024 Jan 1;31(Pt 1):150–61. doi: 10.1107/S1600577523009876 (PMC10833432; doi:10.1107/S1600577523009876)
Supplement: Supplementary file 4 [file s-31-00150-sup4.zip › HM_3D_CAD_drawings/Bottom Platen.pdf]

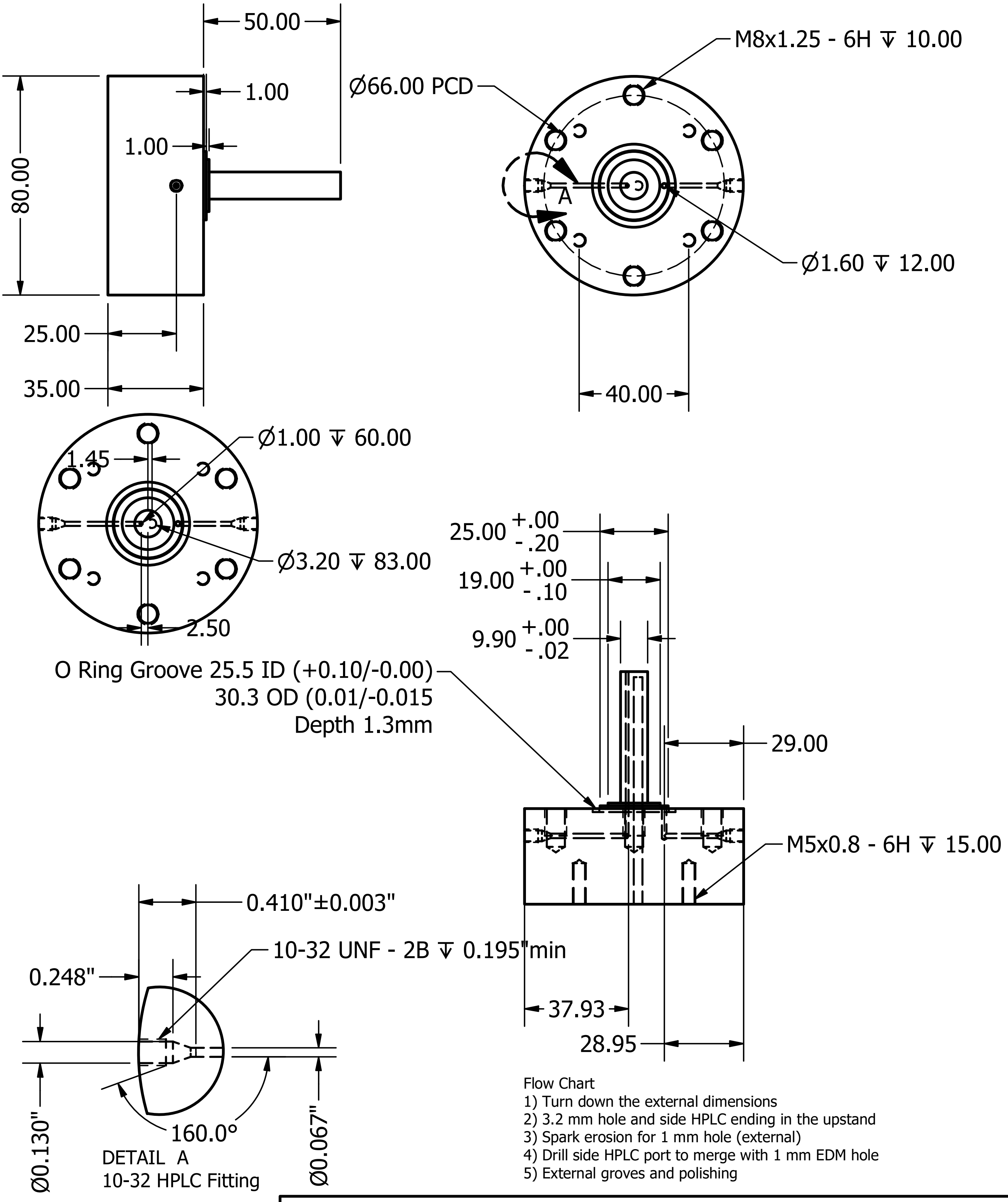

Flow Chart
1) Turn down the external dimensions
2) 3.2 mm hole and side HPLC ending in the upstand
3) Spark erosion for 1 mm hole (external)
4) Drill side HPLC port to merge with 1 mm EDM hole
5) External groves and polishing

Unless otherwise specified dimensions are in millimeters

The information contained in this drawing is the sole property of The University Of Edinburgh. Any reproduction in part or whole without written permission of The University of Edinburgh is prohibited

Description

Designed by Damien Freitas/Ian Butler<br>Date 21-09-2023		Draftsman Chris McCartney<br>Customer Damien Freitas		Heat Treatment/ Surface Treatment	Format A4<br>Scale 1:1	
- ÷ XX	XX ÷ XXX	XXX ÷ XXXX	OVER XXXX	Material<br>Grade 5 Titanium	Weight	Quantity
±0.1	±0.2	±0.5	±1			
**The University of Edinburgh**<br>School of Geosciences				Part Code		Job Code
				Part Name Bottom Piston/Platen		Sheet
