# Supplementary material for: Heitt Mjölnir: a heated miniature triaxial apparatus for 4D synchrotron microtomography
Source: J Synchrotron Radiat. 2024 Jan 1;31(Pt 1):150–61. doi: 10.1107/S1600577523009876 (PMC10833432; doi:10.1107/S1600577523009876)
Supplement: Supplementary file 4 [file s-31-00150-sup4.zip › HM_3D_CAD_drawings/LDVT Tell-Tale Brass Cap.pdf]

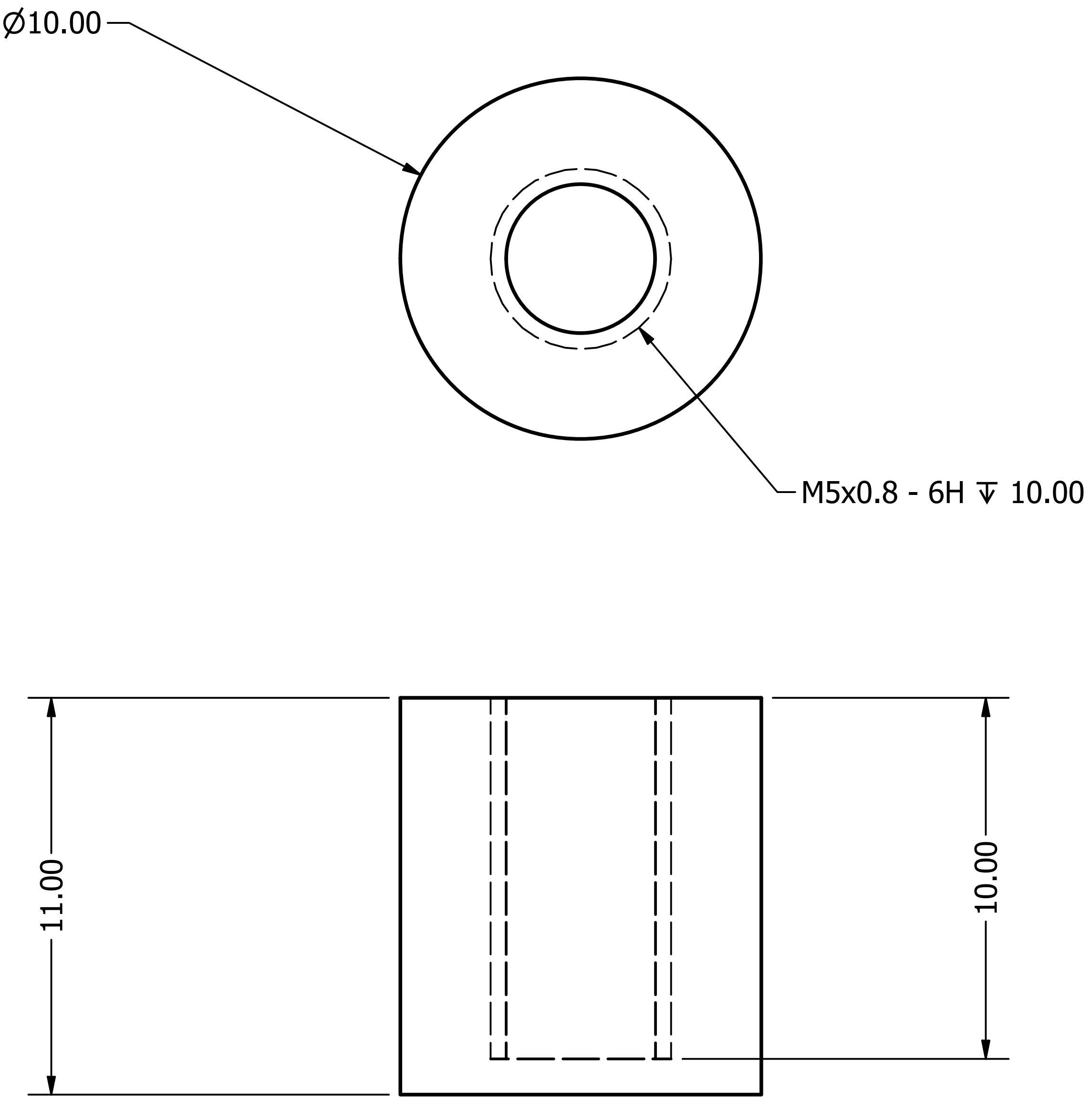

Unless otherwise specified dimensions are in millimeters

The information contained in this drawing is the sole property of The University Of Edinburgh. Any reproduction in part or whole without written permission of The University of Edinburgh is prohibited

Description

Designed by<br>Damien Freitas/Ian Butler<br>Date<br>21-09-2023	Draftsman<br>Chris McCartney<br>Customer<br>Damien Freitas	Heat Treatment/<br>Surface Treatment	Format<br>A4<br>Scale<br>1:1

- ÷ XX	XX ÷ XXX	XXX ÷ XXXX	OVER XXXX
±0.1	±0.2	±0.5	±1

Material	Weight	Quantity
Brass		

The University of Edinburgh
School of Geosciences

Part Code

Job Code

Part Name: LVDT Tell-Tale Brass Cap

Sheet
