# Supplementary material for: Heitt Mjölnir: a heated miniature triaxial apparatus for 4D synchrotron microtomography
Source: J Synchrotron Radiat. 2024 Jan 1;31(Pt 1):150–61. doi: 10.1107/S1600577523009876 (PMC10833432; doi:10.1107/S1600577523009876)
Supplement: Supplementary file 4 [file s-31-00150-sup4.zip › HM_3D_CAD_drawings/Metal Jacket.pdf]

(As coming from supplier the pistons OD must be adapter if Jacket's ID is different)

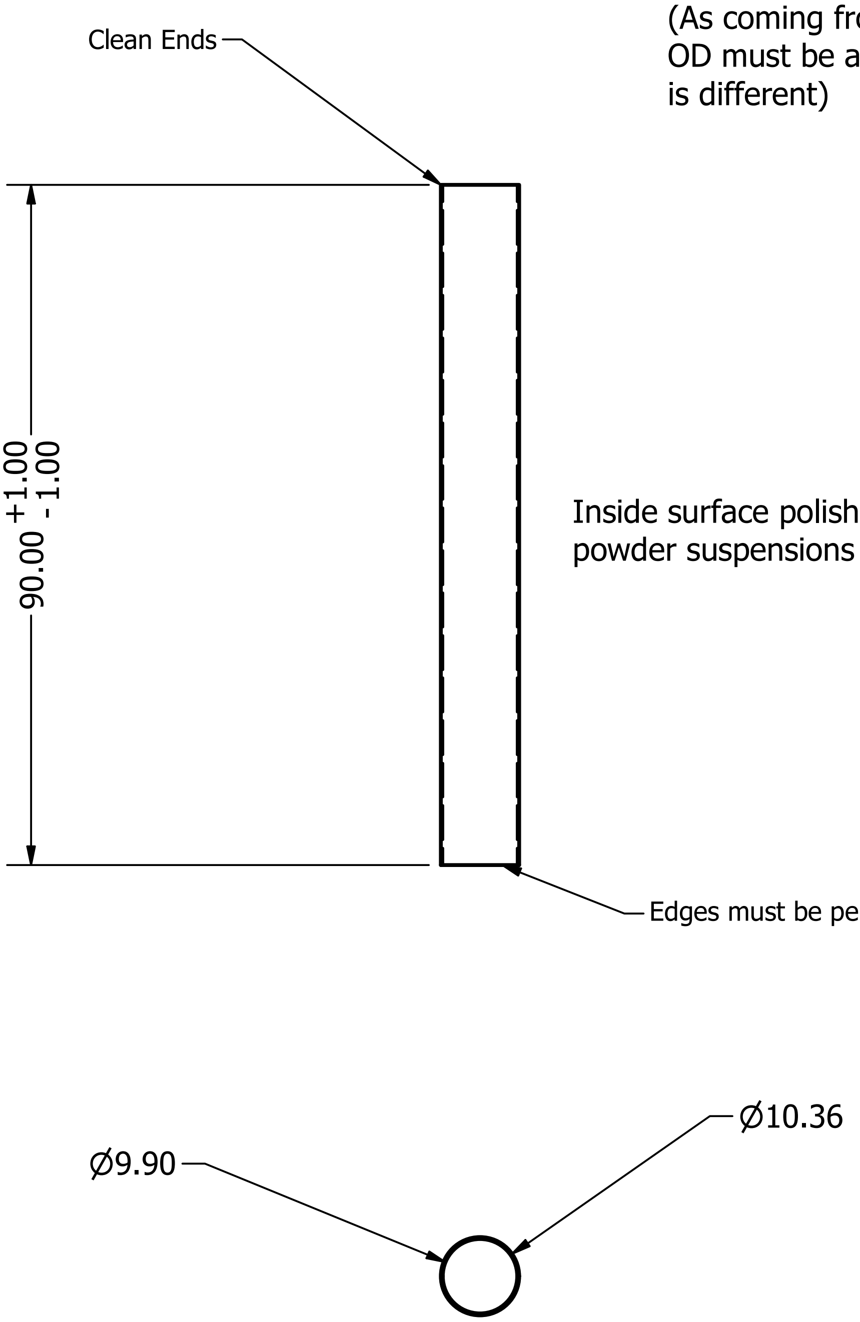

Inside surface polished with Diamond powder suspensions up to 1/4 microns

Obtained from Goodfellow Copper tubes
(Ref: CU00-TB-000145 Copper)

Unless otherwise specified dimensions are in millimeters

Description

Designed by Damien Freitas/Ian Butler<br>Date 21-09-2023		Draftsman Chris McCartney<br>Customer Damien Freitas		Heat Treatment/ Surface Treatment	Format A4<br>Scale 1:1
- ÷ XX	XX ÷ XXX	XXX ÷ XXXX	OVER XXXX	Material Copper	Weight	Quantity
±0.1	±0.2	±0.5	±1			

The University of Edinburgh
School of Geosciences

Part Code

Job Code

Part Name: Metal Jacket

Sheet

The information contained in this drawing is the sole property of The University Of Edinburgh. Any reproduction in part or whole without written permission of The University of Edinburgh is prohibited
