# Supplementary material for: Heitt Mjölnir: a heated miniature triaxial apparatus for 4D synchrotron microtomography
Source: J Synchrotron Radiat. 2024 Jan 1;31(Pt 1):150–61. doi: 10.1107/S1600577523009876 (PMC10833432; doi:10.1107/S1600577523009876)
Supplement: Supplementary file 4 [file s-31-00150-sup4.zip › HM_3D_CAD_drawings/Piston Guide.pdf]

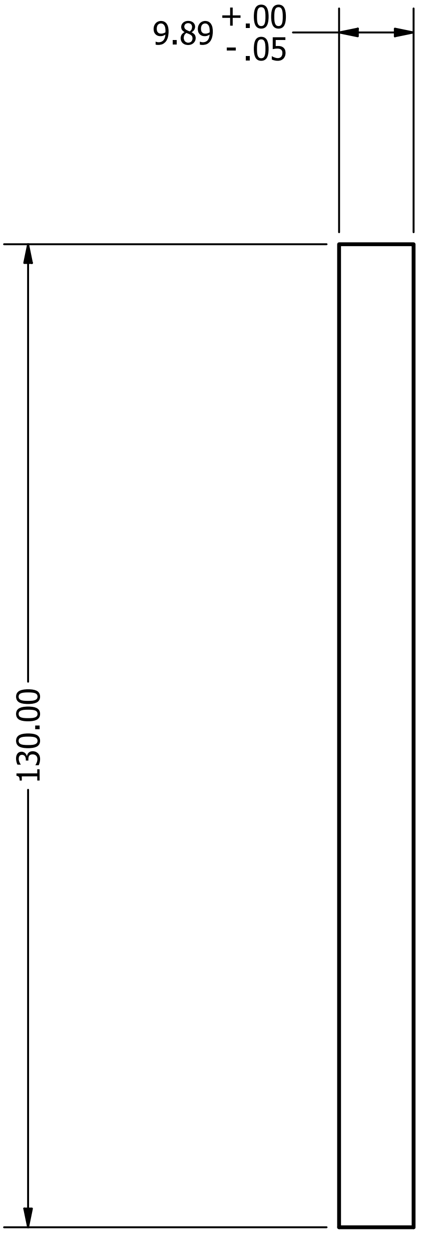

Slightly smaller than pistons,
Mirror polished,
Used to gauge the jackets
& assemble

Unless otherwise specified dimensions are in millimeters

The information contained in this drawing is the sole property of The University Of Edinburgh. Any reproduction in part or whole without written permission of The University of Edinburgh is prohibited

Description

Designed by Damien Freitas/Ian Butler<br>Date 21-09-2023	Draftsman Chris McCartney<br>Customer Damien Freitas	Heat Treatment/ Surface Treatment	Format A4<br>Scale 1:1

- ÷ XX	XX ÷ XXX	XXX ÷ XXXX	OVER XXXX
±0.1	±0.2	±0.5	±1

Material<br>Stainless Steel	Weight	Quantity

The University of Edinburgh
School of Geosciences

Part Code	Job Code
Part Name: Piston Guide	Sheet
