# Supplementary material for: Heitt Mjölnir: a heated miniature triaxial apparatus for 4D synchrotron microtomography
Source: J Synchrotron Radiat. 2024 Jan 1;31(Pt 1):150–61. doi: 10.1107/S1600577523009876 (PMC10833432; doi:10.1107/S1600577523009876)
Supplement: Supplementary file 4 [file s-31-00150-sup4.zip › HM_3D_CAD_drawings/Sample Polishing Guide.pdf]

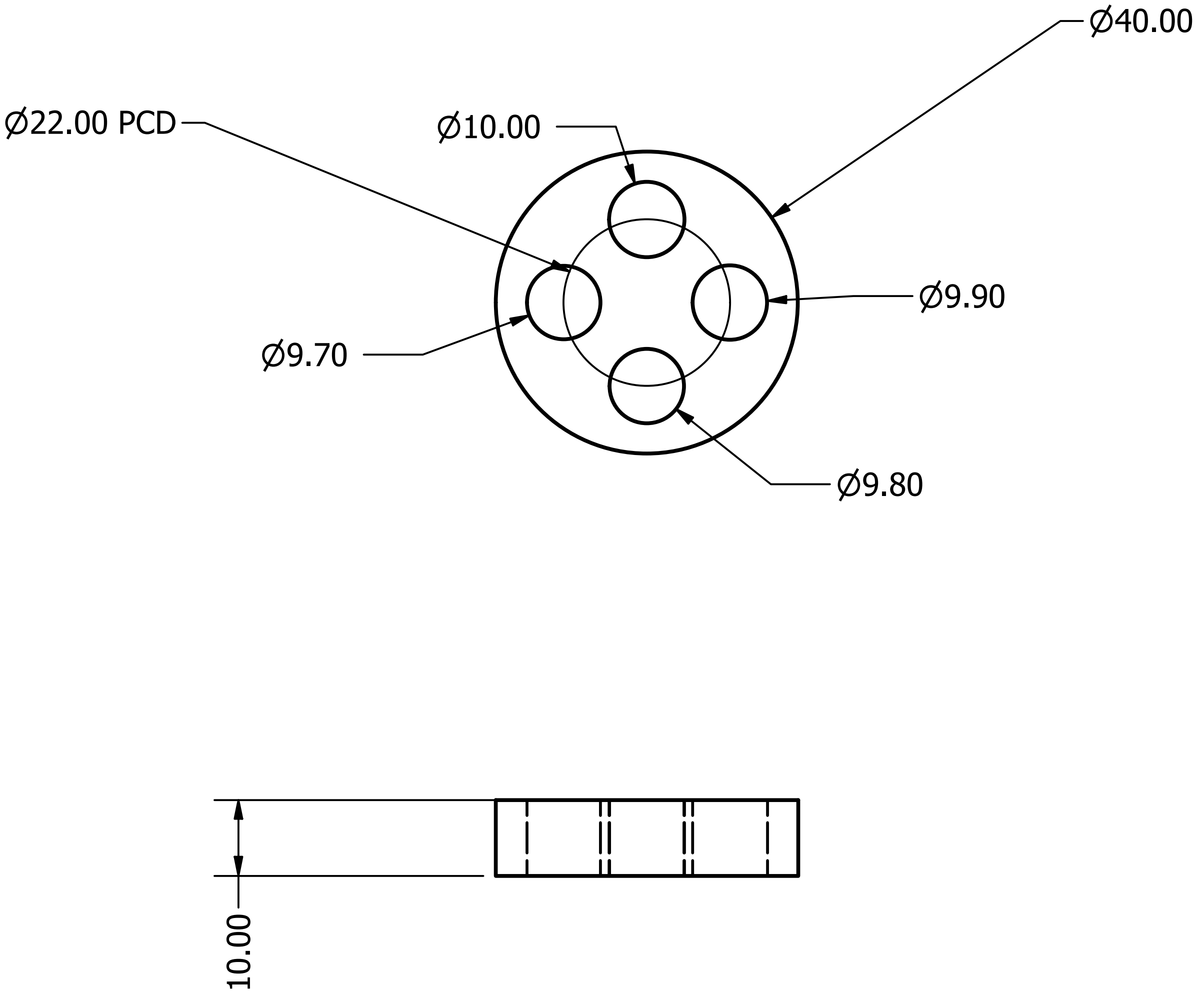

Unless otherwise specified dimensions are in millimeters

The information contained in this drawing is the sole property of The University Of Edinburgh. Any reproduction in part or whole without written permission of The University of Edinburgh is prohibited

Description

Designed by
Damien Freitas/Ian Butler
Date
21-09-2023

Draftsman
Chris McCartney
Customer
Damien Freitas

Heat Treatment/
Surface Treatment

Format
A4

Scale
1:1

- ÷ XX	XX ÷ XXX	XXX ÷ XXXX	OVER XXXX
±0.1	±0.2	±0.5	±1

Material
Stainless Steel

Weight

Quantity

The University of Edinburgh
School of Geosciences

Part Code

Job Code

Part Name
Sample Polishing Guide

Sheet
