# Supplementary material for: Heitt Mjölnir: a heated miniature triaxial apparatus for 4D synchrotron microtomography
Source: J Synchrotron Radiat. 2024 Jan 1;31(Pt 1):150–61. doi: 10.1107/S1600577523009876 (PMC10833432; doi:10.1107/S1600577523009876)
Supplement: Supplementary file 4 [file s-31-00150-sup4.zip › HM_3D_CAD_drawings/Seal Retainer.pdf]

Made to fit Top Platen
Each top platen has its own Retainer

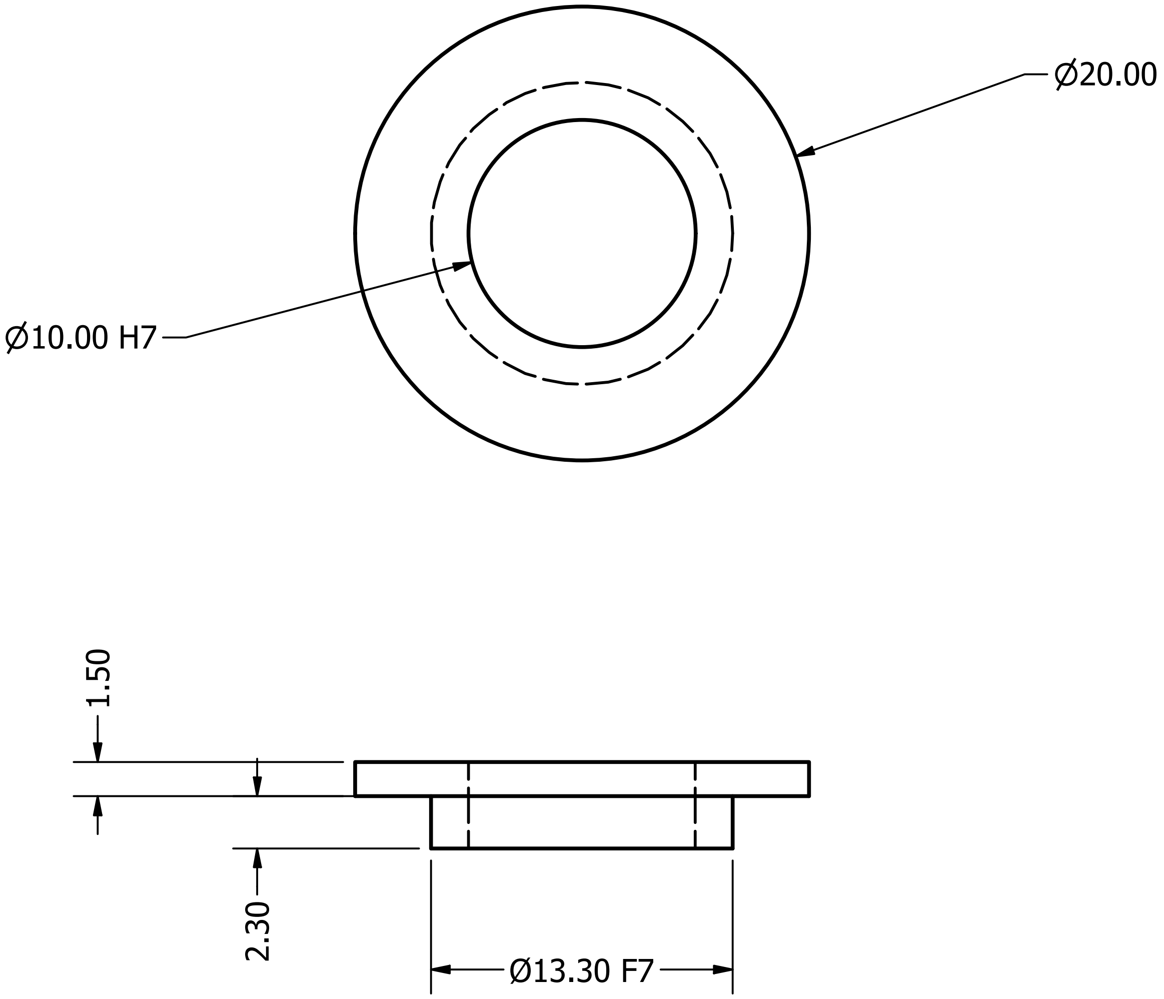

Unless otherwise specified dimensions are in millimeters

The information contained in this drawing is the sole property of The University Of Edinburgh. Any reproduction in part or whole without written permission of The University of Edinburgh is prohibited

Description

Designed by Damien Freitas/Ian Butler<br>Date 21-09-2023		Draftsman Chris McCartney<br>Customer Damien Freitas		Heat Treatment/ Surface Treatment	Format A4<br>Scale 1:1	
- ÷ XX	XX ÷ XXX	XXX ÷ XXXX	OVER XXXX	Material<br>Phosphor Bronze	Weight	Quantity
±0.1	±0.2	±0.5	±1			
**The University of Edinburgh**<br>School of Geosciences				Part Code		Job Code
				Part Name Seal Retainer		Sheet
