# Supplementary material for: Heitt Mjölnir: a heated miniature triaxial apparatus for 4D synchrotron microtomography
Source: J Synchrotron Radiat. 2024 Jan 1;31(Pt 1):150–61. doi: 10.1107/S1600577523009876 (PMC10833432; doi:10.1107/S1600577523009876)
Supplement: Supplementary file 4 [file s-31-00150-sup4.zip › HM_3D_CAD_drawings/Spacer.pdf]

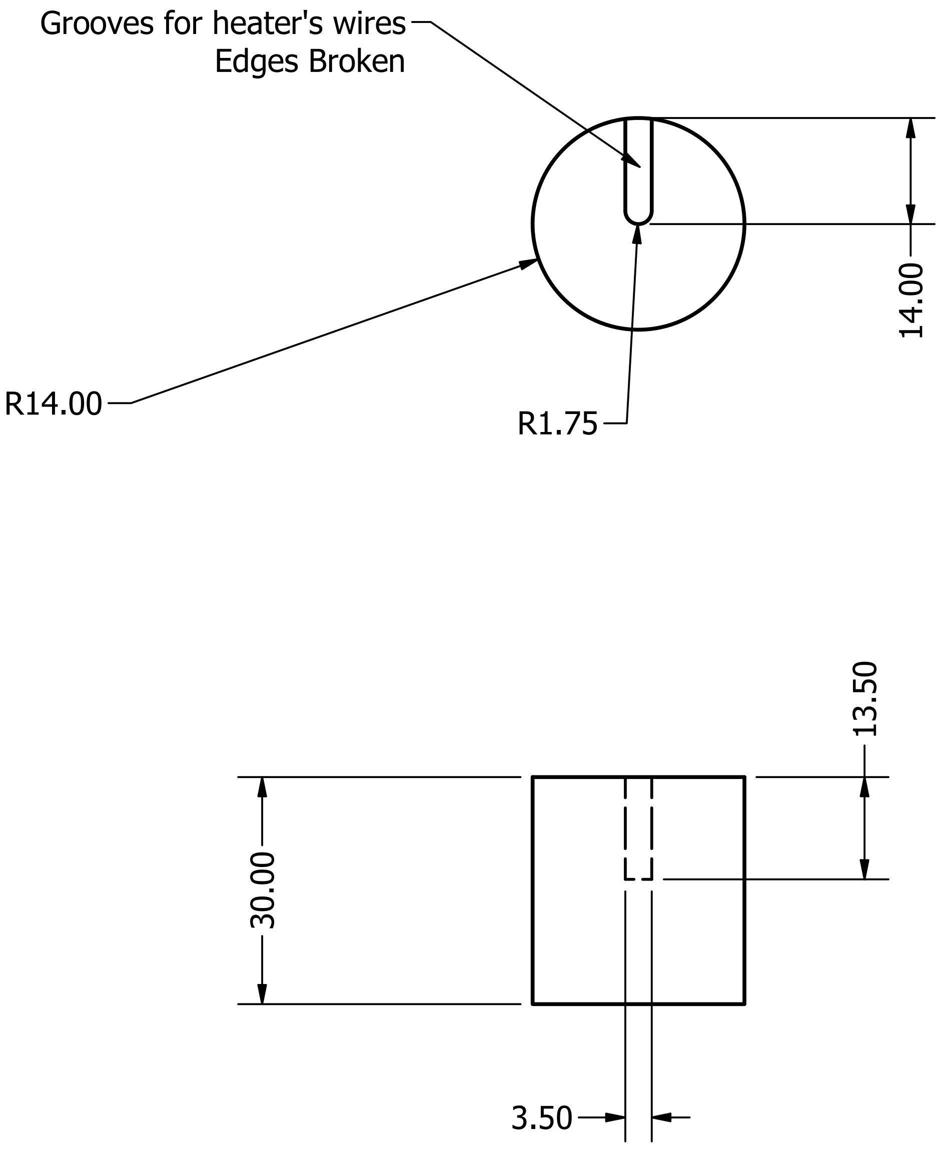


Unless otherwise specified dimensions are in millimeters

The information contained in this drawing is the sole property of The University Of Edinburgh. Any reproduction in part or whole without written permission of The University of Edinburgh is prohibited

## Description

| | | | | | | |
|---|---|---|---|---|---|---|
| Designed by<br>Damien Freitas/Ian Butler<br>Date<br>21-09-2023 | | Draftsman<br>Chris McCartney<br>Customer<br>Damien Freitas | | Heat Treatment/<br>Surface Treatment | Format<br>A4<br>Scale<br>1:1 | |
| - ÷ XX | XX ÷ XXX | XXX ÷ XXXX | OVER XXXX | Material<br>Stainless Steel | Weight | Quantity |
| ±0.1 | ±0.2 | ±0.5 | ±1 | | | |
| The University of Edinburgh<br>School of Geosciences | | | | Part Code | | Job Code |
| | | | | Part Name<br>Spacer | | Sheet |
