# Supplementary material for: Heitt Mjölnir: a heated miniature triaxial apparatus for 4D synchrotron microtomography
Source: J Synchrotron Radiat. 2024 Jan 1;31(Pt 1):150–61. doi: 10.1107/S1600577523009876 (PMC10833432; doi:10.1107/S1600577523009876)
Supplement: Supplementary file 4 [file s-31-00150-sup4.zip › HM_3D_CAD_drawings/Top Platen.pdf]

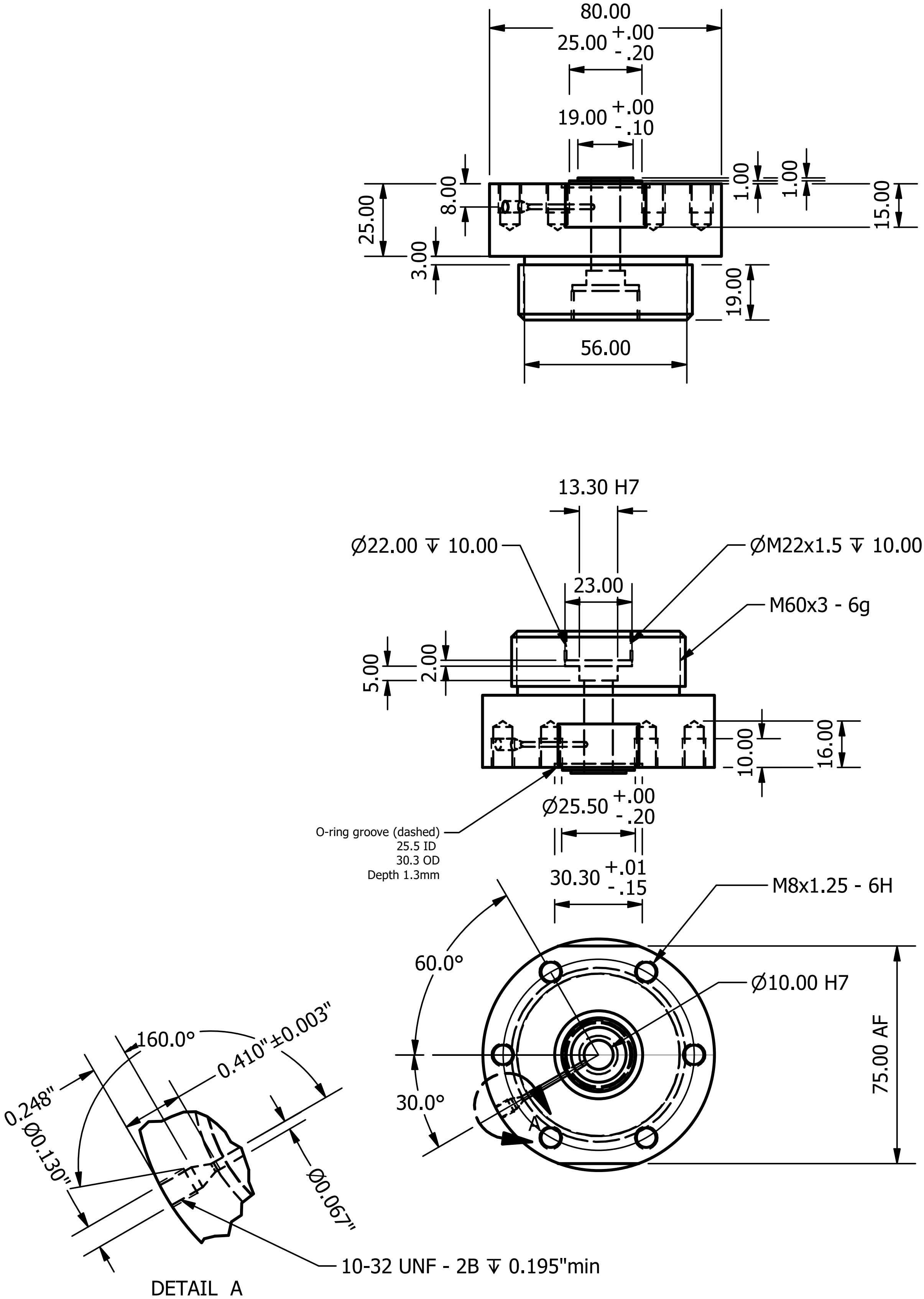

DETAIL A
10-32 HPLC Fitting

Unless otherwise specified dimensions are in millimeters

The information contained in this drawing is the sole property of The University Of Edinburgh. Any reproduction in part or whole without written permission of The University of Edinburgh is prohibited

Description

Designed by Damien Freitas/Ian Butler<br>Date 21-09-2023	Draftsman Chris McCartney<br>Customer Damien Freitas	Heat Treatment/ Surface Treatment	Format A4<br>Scale 1:1

- ÷ XX	XX ÷ XXX	XXX ÷ XXXX	OVER XXXX
±0.1	±0.2	±0.5	±1

Material: Grade 5 Titanium

Weight

Quantity

The University of Edinburgh
School of Geosciences

Part Code

Job Code

Part Name: Top Platen

Sheet
